# The validity and safety of multispectral light emitting diode (LED) treatment on grade 2 pressure ulcer: Double-blinded, randomized controlled clinical trial

**DOI:** 10.1371/journal.pone.0305616

**Published:** 2024-08-23

**Authors:** Nam Kyu Lim, Hyeyoon Goo, Sung-Ryeong Yoon, Jin Chul Ahn, Namgue Hong, Young Hoon Choi, Hyung Bin Bang, Sungyeon Kim, Yong Won Choi, Phil-Sang Chung

**Affiliations:** 1 Department of Plastic and Reconstructive Surgery, Dankook University Hospital, Cheonan, Chungnam, Republic of Korea; 2 Department of Medical Laser, Graduate School of Medicine, Dankook University, Cheonan, Chungnam, Republic of Korea; 3 Medical Laser Research Center, Dankook University, Cheonan, Chungnam, Republic of Korea; 4 Institute of Medical Science, Dankook University Hospital, Cheonan, Chungnam, Republic of Korea; 5 Linkoptics Inc., Gwangju, Cheonnam, Republic of Korea; 6 Beckman Laser Institute Korea, Dankook University College of Medicine, Cheonan, Chungnam, Republic of Korea; 7 Department of Otolaryngology-Head & Neck Surgery, Dankook University College of Medicine, Cheonan, Chungnam, Republic of Korea; Massachusetts General Hospital, UNITED STATES

## Abstract

**Purpose:**

The management of pressure ulcers (PUs) poses challenges due to their chronic nature and the lack of established conservative treatment methods. In this clinical trial, our objective was to examine the validity and safety of using a light-emitting diode device contained four wavelengths in the treatment of grade 2 sacral PUs.

**Method:**

A total of 38 patients were randomly assigned to two groups: sham device (Sham) and experimental device (LED) group. The treatment sessions were conducted over a period of four weeks, with a frequency of three times per week. The study was conducted in a double-blinded manner. The study assessed the primary validity by measuring wound size and re-epithelialization after 0 and 4 weeks. Secondary evaluations included epidermal regeneration, collagen density, and immunological markers. Safety was evaluated by monitoring adverse reactions throughout the trial.

**Result:**

The presence of eschar was found to have a significant impact on wound healing. Sham consisted of 15 wounds without eschar, while LED had nine. After treatment in without eschar situation, the post-treatment size of wounds in Sham was 13.80 ± 20.29%, while it was 3.52 ± 6.68% in LED. However, there was no significant difference (*p* = 0.070). And analysis of epidermal thickness showed a significant increase in LED (495.62 ± 327.09 μm) compared to Sham (195.36 ± 263.04 μm) (*p* < 0.0001).

**Conclusion:**

While LED treatment had a potential for wound reduction in PUs without eschar, we could not uncover evidence to support the efficacy of LED treatment in grade 2 PUs.

## Introduction

In the United States, pressure ulcers have become a pressing public health concern, impacting approximately 2.5 million individuals annually and tragically leading to around 60,000 deaths. These wounds pose a significant societal burden, with an annual cost reaching approximately $11.6 billion [1–3]. There are various ways to classify pressure ulcers, the most used method in recent years is the grade classification system published by the National Pressure Ulcer Advisory Panel [1]. Grade 2 ulcers result in skin damage, and if left untreated, they can escalate to grade 3 or higher, leading to tissue necrosis [1, 4]. Pressure ulcers are typical chronic wounds, and its recovery is not always achieved and requires a combination of factors such as reducing direct pressure, managing underlying medical conditions and nutrition, and continuous wound management until relief from sustained pressure is achieved [2, 4, 5]. Though many dressing materials have been developed, there is no established method for the conservative treatment of pressure ulcers, as each treatment has its limitations [1, 2, 6].

Numerous reports have suggested that photobiomodulation therapy (PBMT) uses low-level light to induce healing and promote oxidative regulation and growth factor production, aiding wound healing [7–11]. It is also known to have anti-inflammatory, analgesic, and antibacterial effects, the effectiveness of PBMT in treating pressure ulcers has been reported [11–13]. In most studies, the treatment was delivered at an energy density of either 1 J/cm^2^ or 4 J/cm^2^, with a frequency of three to five sessions per week over 4 to 6 weeks. And these therapies utilized wavelengths ranging from 650 to 820 nm [10, 11]. In PBMT, wavelength is an important parameter alongside energy density. Several reports suggested that the 630 – 660 nm range, known as the ‘RED’ region, was favorable for fibroblast proliferation and wound healing [10, 14, 15]. Additionally, wavelengths in the 810 – 850 nm range, referred to as infrared, have good penetration capabilities and can affect regeneration up to the deep dermal layer [15, 16]. Meanwhile, UV or blue wavelengths were reported to antimicrobial effects [17, 18]. We also performed preclinical test in 2019 using rats, and found that combination therapy with plasma sterilization and multiple wavelengths (592 and 630 nm) was significantly induced infected wound compared to the single treatment group **(S1 Fig)**. A simultaneous multi-wavelength combination therapy had distinct effects on cell proliferation, differentiation and signaling compared to single wavelengths, which have been reported in several literatures [19–21]. However, the use of plasma to induce sterilization is not suitable for clinical trials due to the potential risk of ozone generation in humans. The clinical trial device, BELLALUX Lite, was a follow-up model of the preclinical test model and had added a 460 nm BLUE wavelength, which can improve sterilization power. Therefore, the basis for the clinical trial has been established.

In this clinical trial, the device used was the "BELLALUX Lite," certified by the Worldwide System for Conformity Testing and Certification of Electrical Equipment (IECEE-CB) in July 2019. It is a medical stimulator that combines four light-emitting diode (LED) wavelengths: visible light (RED [630 nm], AMBER [595 nm]; BLUE [460 nm]) and near-infrared light (850 nm). This Grade 2 medical device has potential antibacterial (BLUE) and wound recovery (RED, near-infrared light) capabilities, particularly for chronic wounds such as pressure ulcers. We aimed to analyze the treatment process to determine the validity and safety of utilizing this device in the second-grade sacral pressure ulcers and assess chronic wound recovery.

## Method

The clinical trial was conducted for 24 months (October 28th, 2020 to October 26th, 2022) in accordance with institutional review board (IRB) approval (DKUH 2020-09-014) and with the Ministry of Food and Drug Safety in Republic of Korea (1104). The study protocol commenced with version 2.0 and underwent two revisions, culminating in version 4.0. Throughout this period, the inclusion criteria were modified from individuals aged 19 years or older to those aged 13 years or older, although no minors participated in the study. The protocol is available as supportive information. All participants provided written informed consent. We had recruited participants from January 11th, 2021 to August 24th, and monitored until the end of study period. This clinical trial was registered in Clinical Research Information Service (CRIS, cris.nih.go.kr), and the registered number is KCT0009002. The reason why time lag between the initial enrollment on January 11th, 2021, and the submission on December 16th, 2021, was primarily prolonged due to the process of revising protocols and obtaining approval from the IRB. The medical and sham device groups were randomly divided into control (Sham) and experimental (LED) groups. The random allocation method was as follows: a subject identification code was assigned when a person met the inclusion criteria and consented to participate. The assignment sequence of the experimental and control groups was randomized based on subject registration numbers. A sufficient number of random assignment numbers were generated, considering the size of the pre-specified block using the statistical software R, version 4.04 (R Foundation, Vienna, Austria). The study was designed a double-blinded manner, with participants (patients) blinded and researcher (NK Lim, et al.) blinded (sham vs. LED). A data analysis and assigning randomized registration number were conducted by a contract research organization (CRO) company. After preparation, a randomization table was managed separately by a third independent randomization management officer (CRO company). A blind test was released at the completion of the clinical trial; however, verifying it before the conclusion in the event of an emergency was permissible.

**Fig 1:**
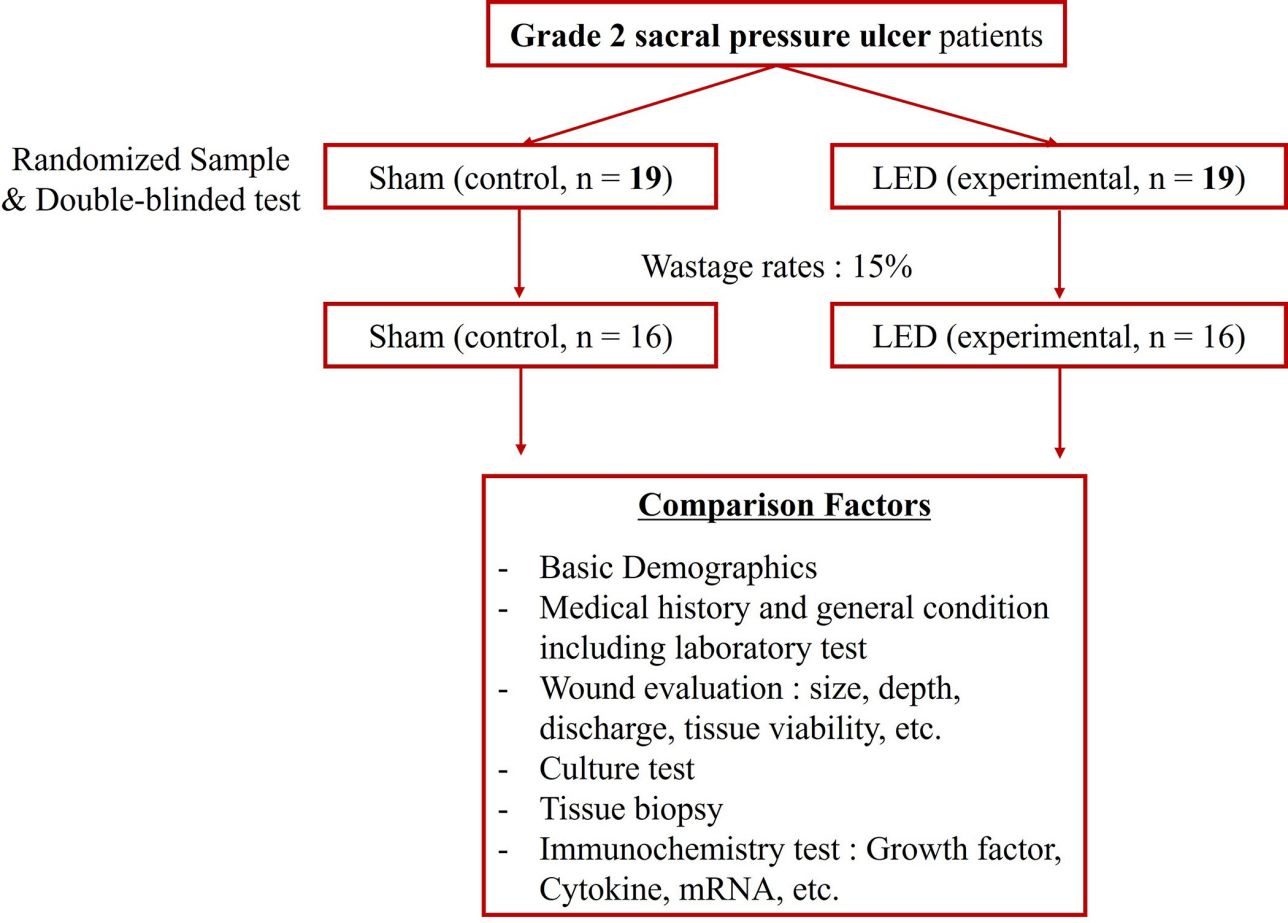
A study design that incorporates group classification.

Considering a 15% dropout rate, the target number of participants was 16 for each final evaluation subject group and 19 per control and experimental group, for 38 participants **(Fig 1)**. The rationale for these 16 patients was based on a systematic review published in 2020 [12]. Participants were sourced through in-person promotion and posters targeting individuals seeking pressure ulcer treatment at Dankook University Hospital, with all details meticulously documented in an electronic chart. The inclusion and exclusion criteria are presented in **Table 1**.

**Table 1:** Inclusion and exclusion criteria in this study.

Criteria	Number	Contents	*Note*
**Inclusion**	1	Individuals affected by the second grade of pressure ulcer (according to NPUAP guidelines)	
	2	those 13 years of age or older	
**Exclusion**	1	pregnant or lactating women	
	2	Patients with a history of sacral pressure ulcer surgery	
	3	Those with osteomyelitis	
	4	those isolated with resistant bacteria	
	5	those unable to remain in a decubitus position for more than 30 minutes	
	6	those who should continue to take immunosuppressants or steroids due to systemic diseases	but can be registered if the drug has a low effect on the immune system
	7	those who are sensitive to light (photosomiasis)	
	8	anyone else deemed by the director of research to be unable to perform clinical trials	

**Fig 2:**
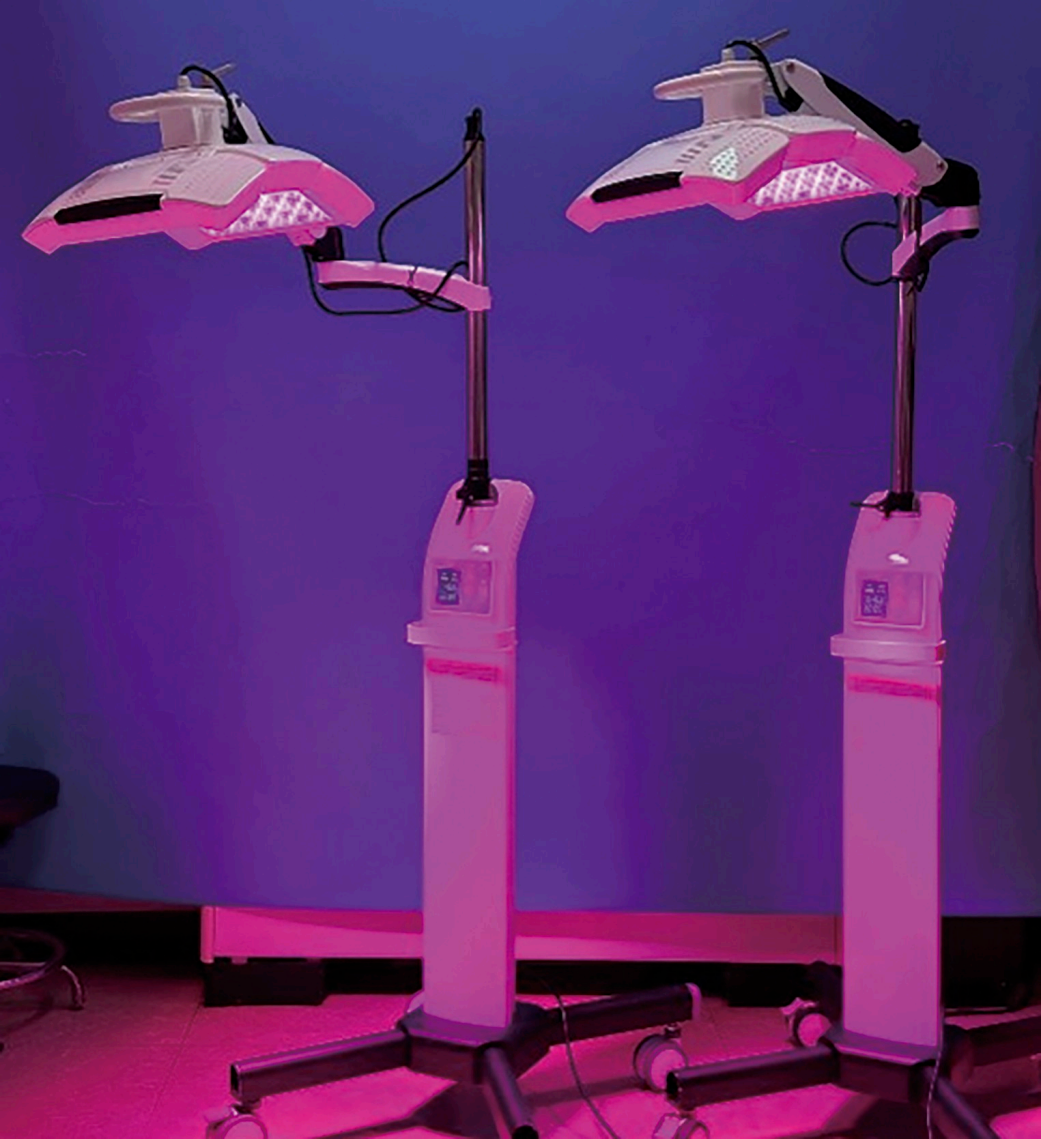
External appearance of both multispectral light emitting diode device (“BELLALUX Lite”) and sham device. The two devices are difficult to distinguish based solely on their appearance aspect.

After conducting the required examinations and testing in compliance with the clinical trial protocol, the participants were exposed to the simultaneous emitting four light wavelengths for 25 min at twelve times per a month. An experimental LED device was set at maximum output with total 90 mW/cm^2^ (**Table 2,** all four wavelengths with 5 stage). Thus, a total energy of 135 J/cm^2^ was applied over 25 minutes (1620 J/cm^2^ total for 12 sessions). On the other hand, a control device (Sham) was equipped with the same shape as that in the experiment **(Fig 2)**, and it implemented the same LED visually as the therapeutic device by using the minimum power (stage 1; total 12 mW/cm^2^ across 4 wavelengths). This translates to a total energy of 18 J/cm^2^ per session, and 216 J/cm^2^ in total over the entire trial period. A light exposure occurred 15 cm from the wound region.

**Table 2:** Power depending on a wavelength at a distance of 15 cm from the irradiation unit.

Wavelength	1 stage	2 stage	3 stage	4 stage	5 stage
**Blue (460 nm)**	0.80	2.20	3.60	5.10	8.40
**Amber (595 nm)**	2.00	4.50	7.00	9.00	12.00
**RED (630 nm)**	3.60	8.80	13.80	19.00	30.00
**NIR (850 nm)**	5.60	12.40	21.00	28.00	40.00

(mW/cm^2^)

All output power has error about 10%

The clinical trial included 15 visits over 210 days. Visit 1 (day 0) was for screening; visits 2 (day 1) through 13 (day 26) were for treatment; and visits 14 (day 29) and 15 (day 210) were for follow-up. After checking the criteria for subject selection during screening (visit 1), LED therapy was administered thrice weekly for 4 weeks between visits 2 and 13. The patient’s medical histories were meticulously researched and recorded during the screening visit using interviews and historical medical documents; this included a history of diabetes, hypertension, surgical history, and drug administration. In addition to blood tests, urine tests, and pelvic radiography, women of reproductive age also underwent pregnancy testing. Before treatment (visit 1) and after treatment (visit 14), bacterial culture tests and tissue biopsies were conducted exclusively on patients with additional consent. At each visit, a physical examination, including an assessment of the affected area, was conducted, including the size (length × width) and depth (superficial, moderate, and deep) of the pressure ulcer, presence of eschar, color, secretion style, odor, microvascular bleeding, and degree of granulation tissue formation. We also applied an antibacterial dressing with 1% silver sulfadiazine plus foam daily following the global guidelines for pressure ulcers published in 2019 to conduct clinical trials with a subject risk below the minimal threshold **(Table 3)** [1]. The validity and safety of the clinical trials utilizing these protocols were evaluated using the following criteria.

**Table 3:** Protocol of clinical trial.

Visit	Screening	Treatment period				Follow up period	
	Visit 1	Visit 2~4 (1st week)	Visit 5~7 (2nd week)	Visit 8~10 (3rd week)	Visit 11~13 (4th week)	Visit 14	Visit 15
Day (D)	D0	D1,D3,D5	D8,D10,D12	D15,D17,D19	D22,D24,D26	D29	D210
**Explanation of clinical trial**	**○**						
**Informed consent and generating screening numbering**	**○**						
**Checking the criteria for subject selection**	**○**						
**Randomization for grouping**	**○**						
**Demographic investigation**	**○**						
**Investigation of past medical history**	**○**						
**Pregnant test (reproductive age woman only)**	**○**						
**Physical examination and checking vital sign**	**○**	**○**	**○**	**○**	**○**	**○**	**○**
**Wound assessment and checking a Braden scale**	**○**	**○**	**○**	**○**	**○**	**○**	**○**
**Laboratory test 1 (Blood test, Urine test)**	**○**					**○**	
**Laboratory test 2 (Pelvic X-ray)**	**○**					**○**	
**Laboratory test 3 (Wound swab culture, tissue biopsy – only performed consent patient)**	**○**					**○**	
**Rechecking the criteria**		**○**					
**LED treatment (apply medical device)**		**○**	**○**	**○**	**○**		
**Investigation of adverse events**		**○**	**○**	**○**	**○**	**○**	

### (1) Validity assessment

#### A. The first validity assessment (wound size)

The primary validity evaluations were wound size and amount of re-epithelialization. The difference between the two groups was determined by calculating the wound size and degree of re-epithelialization in the control and experimental groups at 0 and 4 weeks. However, because the wound area was reduced during biopsy at visit 1, the initial reference was set at visit 2, just before LED treatment, to avoid bias. A Wilcoxon rank-sum test was conducted to assess the difference in wound area before and after the treatment, while the Mann–Whitney U test was employed to compare the control and experimental groups. In addition, repeated-measures analysis of variance was employed to compare the extent of recovery observed during the treatment period. Statistical analyses were performed using SPSS 23.0 (IBM Corporation, Armonk, NY, USA).

#### B. The second validity assessment (Tissue biopsy)

Secondary validity evaluation assessed the degree of epidermal regeneration, collagen density, and pro- (interleukin [IL]-6) and anti-inflammatory (IL-10) immunochemical indicators in biopsy tissue samples from the control and experimental groups after 0 and 4 weeks. The tissue was harvested until the subcutaneous level in two spots at the pressure ulcer margin using 4-mm punch biopsies, and repair was subsequently performed with a proline 4-0 thread. All samples were stained and subjected to a quantitative real-time polymerase chain reaction using standard methods **(Table 4)**. Data were analyzed using the GraphPad Prism software (GraphPad, San Diego, CA, USA). Significant differences between groups A and B were statistically analyzed using the Mann–Whitney test and was set at a *p-*value of < 0.05 as statistical significance.

**Table 4:** The primer sequences of quantitative real time polymerase chain reaction (qRT-PCR).

Gene name	Forward primer sequences	Reverse primer sequence
**GAPDH**	TTCAACGGCACAGTCAAGG	CTCAGCACCAGCATCACC
**IL-6**	CCCTGCAGCTGGAGAGTGTGG	TGTGCTCTGCTTGAGAGGTGCT
**IL-10**	CAGCAAAGGCCATTCCATCC	GCCTGGGGCATCACTTCTAC

GAPDH, Glyceraldehyde 3-phosphate dehydrogenase; IL, interleukin

### (2) Safety assessment

The clinical trial’s design aimed to assess safety by carefully evaluating and monitoring adverse reactions throughout the study. All adverse and unexpected reactions caused by the clinical trial medical devices were classified as adverse device effects to confirm causality with the clinical trial medical devices. The degree of relevance (definitely related, unrelated, probably related, unrelated, or unknown) was assessed.

## Results

### (1) Subjects’ demographics

Of the 40 subjects who expressed interest in participating, two dropped out of the screening, and 38 were ultimately enrolled in the study, with 19 in each of the two groups. The sex ratio (male: female) in Sham was 6:13, whereas it was 9:10 in LED (*p* = 0.508), and the mean age in Sham was 77.84 ± 10.89 years, whereas in LED, it was 60.47 ± 13.03 years (*p* = 0.000). We also assessed comorbidities in all patients at visit 1 according to the system organ class of the medical dictionary for regulator activities, a total of 75 (sham) and 84 (LED) comorbidities were observed, respectively (for example, hypertension: 15 in sham, 9 in LED; diabetes: 7 in sham, 9 in LED). Numerical information for each disease category is presented in **Table 5** and detailed disease information is listed in **S1 Table**.

**Table 5:** Numerical information for comorbidities in both groups.

System of care criteria	Sham		LED	
	N	%	N	%
	75	100	84	100
Cardiac disorders	4	5.33	2	2.38
Endocrine disorders	0	0.00	1	1.19
Gastrointestinal disorders	3	4.00	2	2.38
General disorders and administration site conditions	0	0.00	1	1.19
Hepatobiliary disorders	0	0.00	1	1.19
Infections and infestations	4	5.33	1	1.19
Injury, poisoning and procedural complications	14	18.67	17	20.24
Investigations	0	0.00	1	1.19
Metabolism and nutrition disorders	7	9.33	9	10.71
Musculoskeletal and connective tissue disorders	2	2.67	3	3.57
Neoplasms benign, malignant, and unspecified	1	1.33	4	4.76
Nervous system disorders	6	8.00	8	9.52
Psychiatric disorders	1	1.33	5	5.95
Renal and urinary disorders	1	1.33	2	2.38
Reproductive system and breast disorders	1	1.33	0	0.00
Respiratory, thoracic, and mediastinal disorders	0	0.00	1	1.19
Surgical and medical procedures	16	21.33	17	20.24
Vascular disorders	15	20.00	9	10.71

Some participants had multiple pressure ulcers in the sacral region, which increased the overall number of wounds in the sham and LED to 25 and 26, respectively. Seven participants in Sham and nine in LED consenting to additional biopsy.

During the 4-week treatment period (visits 2–13), there were six dropouts in Sham and five in LED, including five withdrawals, four with legal isolates (coronavirus disease 19 and vancomycin-resistant Enterococcus), and two patients whose health deteriorated to the point where they were unable to continue.

**Fig 3:**
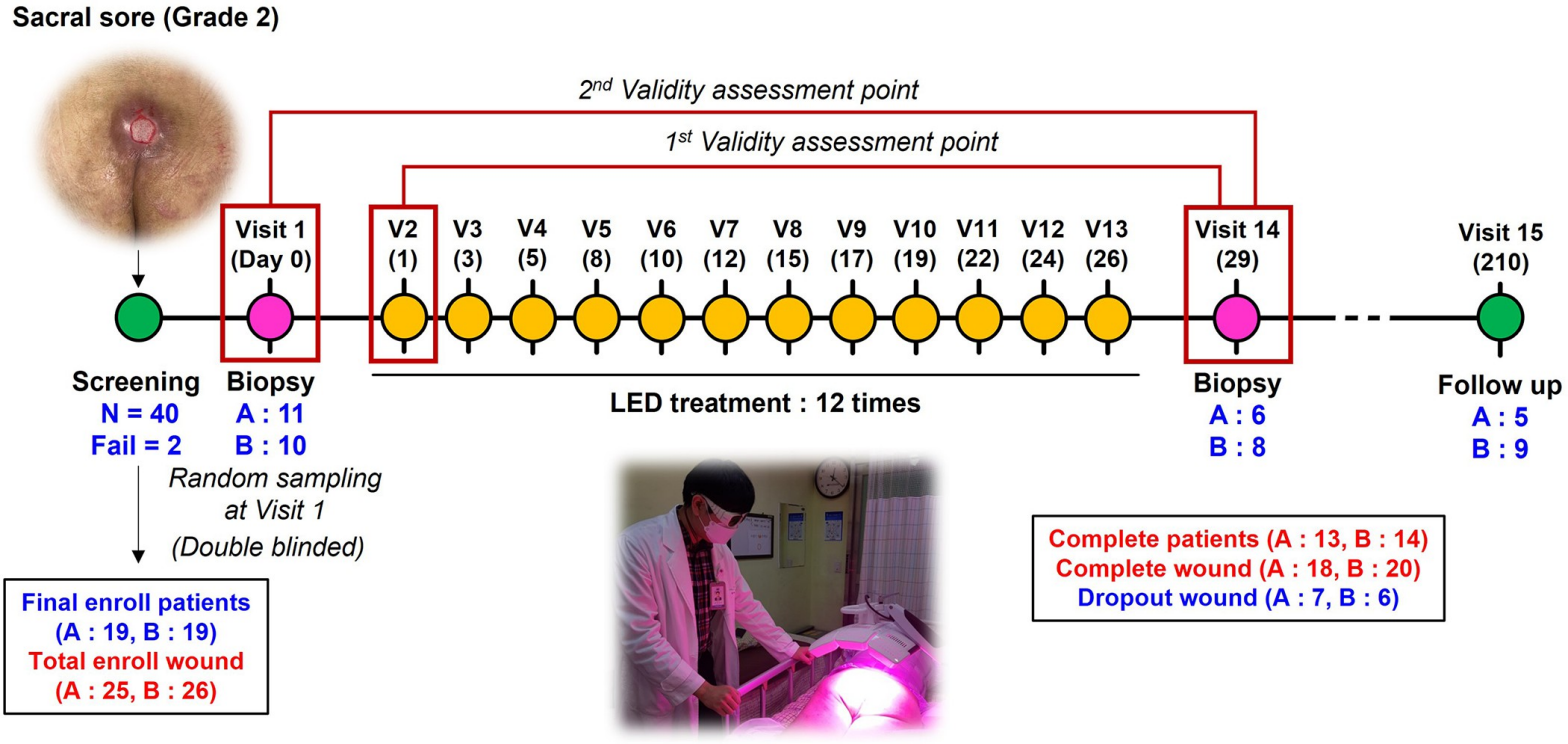
Scheme of clinical trial protocol. V, visit; D, day; N, numbers; A, Sham; B, LED; LED, light emitting diode.

The number of patients who completed the protocol treatment and were eligible for the final evaluation was 13 (three males, 10 females) in Sham and 14 (eight males, six females) in LED, with no statistically significant difference between the two groups (*p* = 0.120). Patients with 18 and 20 wounds in control and experimental groups, respectively, were clinically evaluated for primary validity. Six patients from Sham and eight from LED underwent a post-treatment biopsy for secondary validity. **(Table 6, Fig 3)**.

**Table 6:** Demographics of subjects.

Timeline			Sham	LED	*p* value	*Note*
Visit 1 (screening)	Total enrolled patients	Number	19 (M : 6, F : 13)	19 (M : 9, F : 10)	0.508	Chi-square test
		Age (Mean ± SD, years) [Min ~ Max]	77.84 ± 10.89 [46 ~ 90]	60.47 ± 13.13 [35 ~ 88]	**< 0.001**	Independent t-test
		Height (Mean ± SD, cm) [Min ~ Max]	158.63 ± 7.17 [147 ~ 175]	162.79 ± 9.94 [150 ~ 180]	0.148	Independent t-test
		Weight (Mean ± SD, kg) [Min ~ Max]	59.03 ± 11.11 [43.0 ~ 85.0]	62.57 ± 14.81 [40.0 ~ 97.8]	0.409	Independent t-test
		Co-morbidity disease cases	75	84		Specific disease list in *supplement 1*
	Total enrolled wound	Number	25	26		
	Initial biopsy	Number	11	10		
	Wound culture (Positive)	Number	7	9		Total numbers : Sham 18, LED 19
Visit 2 ~ Visit 13	Drop out patients	Number	6	5		1. Withdrawal of consent : 5 patients
						2. Legal communicable disease (COVID-19, VRE) : 4 patients
						3. Deterioration of health : 2 patients
Visit 14	Complete patients	Number	13 (M : 3, F : 10)	14 (M : 8, F : 6)	0.120	Chi-square test
		Age (Mean ± SD, years) [Min ~ Max]	78.08 ± 8.12 [61 ~ 90]	59.21 ± 11.73 [35 ~ 81]	**< 0.001**	Independent t-test
		Height (Mean ± SD, cm) [Min ~ Max]	158.46 ± 6.57 [150 ~ 175]	164.86 ± 9.49 [154 ~ 180]	0.054	Independent t-test
		Weight (Mean ± SD, kg) [Min ~ Max]	58.73 ± 13.00 [43.0 ~ 85.0]	64.73 ± 16.03 [40.0 ~ 97.8]	0.298	Independent t-test
	**Complete wound**	**Number**	**18**	**20**		**1st validity assessment indicator**
	**Final biopsy**	**Number**	**6**	**8**		**2nd validity assessment indicator**
	Wound culture (Positive)	Number	4	9		Total numbers : Sham 18, LED 19
Visit 15	210 days follow up patients	Number	5	9		Death during V14 ~ V15 : 2 patients

M, male; F, female; V, visit; SD, standard deviation; COVID, coronavirus; VRE, vancomycin resistant enterococcus

Sham, Control group; LED, Experimental group

The medical device group and the sham device group were divided randomly as the control (Sham) and experiment (LED) groups, respectively. The clinical trial was designed with 15 visits over 210 days. A visit 1 (day 0) is for screening, visit 2 (day 1) through 13 (day 26) are for treatment, and visit 14 (day 29) and 15 (day 210) are for follow-up. After checking the criteria for subject selection during screening (visit 1), LED therapy was administered three times per week for four weeks between visit 2 and 13. Of the total 40 subjects who expressed interest in participating, 2 subjects dropped out of the screening, and 38 subjects were ultimately enrolled in the study, 19 in each of the two groups. Some participants had multiple pressure ulcers in the sacral region, which raised the overall number of wounds in the control (Sham) and experimental (LED) groups to 25 and 26, respectively. During the 4-week treatment period (visit 2 through 13), there were 6 dropouts in the Sham and 5 in the LED, including 5 withdrawals, 4 with legal isolates (COVID-19, vancomycin resistant enterococcus), and 2 patients whose health deteriorated to the point where they were unable to continue. Finally, patients with 18 wounds in the Sham and 20 wounds in the LED were evaluated clinically for the primary validity (wound size), and 6 in Sham and 8 in LED for the secondary validity (tissue biopsy). Six months following treatment, long-term monitoring was conducted; two deaths occurred between visit 14 and 15, and the number of patients in long-term observation was five in the Sham and nine in the LED.

### (2) The first validity assessment (Wound size)

#### A. Total wound

At visit 2, the average size (length × width) of the 18 lesions in Sham was 5.02 ± 6.25 cm^2^, while the average size of the 20 lesions in LED was 11.43 ± 15.12 cm^2^. After 12 LED treatments (visit 14), the size of Sham was 0.88 ± 1.22 cm^2,^ and LED was 3.78 ± 7.54 cm^2^. The mean difference ‘LED – Sham’ was 2.90 cm^2^ at visit 14, and there was no significant difference between the two groups (*p* = 0.061). When the initial size of both groups was set to 100%, the post-treatment size was 12.85 ± 18.74% for Sham and 34.46 ± 20.84% for LED. In both groups, there was a significant difference in wound size before and after treatment (Sham, *p* < 0.001; LED, *p* < 0.001) **(Table 7)**.

**Table 7:** Wound size between two groups.

	Total			Without eschar			With eschar		
	Sham (n = 18)	LED (n = 20)		Sham (n = 15)	LED (n = 9)		Sham (n = 3)	LED (n = 11)	
	Mean ± SD (cm^2^)	Mean ± SD (cm^2^)	*p* value	Mean ± SD (cm^2^)	Mean ± SD (cm^2^)	*p* value	Mean ± SD (cm^2^)	Mean ± SD (cm^2^)	*p* value
			0.061			0.070			0.148
V1	5.81 ± 6.10	11.76 ± 15.13		6.19 ± 6.54	11.89 ± 13.36		3.91 ± 3.24	11.65 ± 17.09	
V2	5.02 ± 6.25	11.43 ± 15.12		5.24 ± 6.75	11.51 ± 13.46		3.91 ± 3.24	11.35 ± 17.01	
V3	4.25 ± 5.47	8.35 ± 14.02		4.37 ± 5.91	4.45 ± 4.56		3.65 ± 3.08	11.55 ± 18.21	
V4	3.39 ± 3.66	8.11 ± 13.54		3.45 ± 3.88	4.83 ± 6.07		3.10 ± 2.82	10.80 ± 17.36	
V5	3.08 ± 3.17	7.33 ± 12.94		3.12 ± 3.35	3.61 ± 5.42		2.85 ± 2.62	10.37 ± 16.49	
V6	2.87 ± 3.05	6.64 ± 12.33		2.98 ± 3.23	2.71 ± 5.38		2.33 ± 2.38	9.86 ± 15.51	
V7	2.58 ± 2.77	6.42 ± 12.27		2.61 ± 2.91	2.45 ± 4.76		2.46 ± 2.38	9.66 ± 15.56	
V8	2.21 ± 2.80	5.55 ± 10.07		2.24 ± 2.96	2.05 ± 4.30		2.09 ± 2.32	8.41 ± 12.56	
V9	1.93 ± 2.72	5.06 ± 9.11		2.07 ± 2.95	1.82 ± 4.07		1.23 ± 1.12	7.72 ± 11.29	
V10	1.63 ± 2.41	5.04 ± 9.89		1.75 ± 2.62	1.38 ± 2.90		1.02 ± 0.95	8.03 ± 12.54	
V11	1.35 ± 2.24	4.67 ± 9.57		1.49 ± 2.43	1.02 ± 2.30		0.67 ± 0.68	7.65 ± 12.16	
V12	1.14 ± 1.77	4.48 ± 9.56		1.26 ± 1.92	0.83 ± 1.87		0.53 ± 0.56	7.46 ± 12.21	
V13	0.92 ± 1.29	3.92 ± 7.61		1.01 ± 1.39	0.66 ± 1.45		0.48 ± 0.49	6.59 ± 9.53	
V14	0.88 ± 1.22	3.78 ± 7.54		0.96 ± 1.31	0.56 ± 1.45		0.48 ± 0.49	6.51 ± 9.46	
…									
V15	0.00 ± 0.00	0.14 ± 0.32		0.00 ± 0.00	0.00 ± 0.00		0.00 ± 0.00	0.25 ± 0.40	
*p* value	< 0.001	< 0.001		< 0.001	< 0.001		0.109	0.003	

Sham, Control group; LED, Experimental group

n, numbers; SD, standard deviation; V, visit

All statistical analysis was conducted using repeat measurement ANOVA

#### B. Regression analysis of variables that contribute to wound healing

As treatment significantly decreased the wound size in both groups, regression analysis was conducted to determine the variables that significantly impacted wound healing. Age, body mass index, Braden Scale, serum protein, serum albumin, hemoglobin, localized infection, the presence or absence of LED therapy, and eschar were included as factors according to previous literature [22–25]. Among these, only the presence of eschar had a statistically significant effect on wound healing **(Table 8)**.

**Table 8:** Regression analysis of variables that contribute to wound healing.

Variables	B	Standard error	β	t	*p* value
(Constant)	-143.292	74.212		-1.931	0.065
Age	0.818	0.608	0.332	1.346	0.191
BMI	-0.432	1.060	-0.071	-0.408	0.687
Braden Scale	0.489	0.382	0.283	1.280	0.213
Protein	8.717	9.106	0.203	0.957	0.348
Albumin	-3.326	11.879	-0.061	-0.280	0.782
Hemoglobin	-0.392	2.644	-0.026	-0.148	0.883
Localized infection	14.496	10.670	0.235	1.359	0.187
LED treatment	19.763	17.364	0.322	1.138	0.266
Eschar	34.706	11.433	0.547	3.036	**0.006**

BMI, body mass index; LED, light emitting diode

In usual, eschar indicates a more advanced grade 2 pressure ulcer [2, 26, 27]. This study divided the depth into three levels, with the eschar visible only at the deepest level. Consequently, superficial to moderate levels can be classified as wounds without eschars, whereas deep levels can be categorized as wounds with eschars. The wounds were evaluated based on the presence or absence of eschar additionally. Eschar was significantly more prevalent in LED than in Sham (*p* = 0.014) **(Table 9)**.

**Table 9:** Comparison analysis of eschar formation between groups.

	Sham		LED		*p* value
	N	%	N	%	
Yes	3	16.7	11	55.0	**0.014** ^*^
No	15	83.3	9	45.0	

Sham, Control group; LED, Experimental group

N, numbers

All statistical analysis was conducted using chi-square test.

#### C. Without eschar

**Fig 4:**
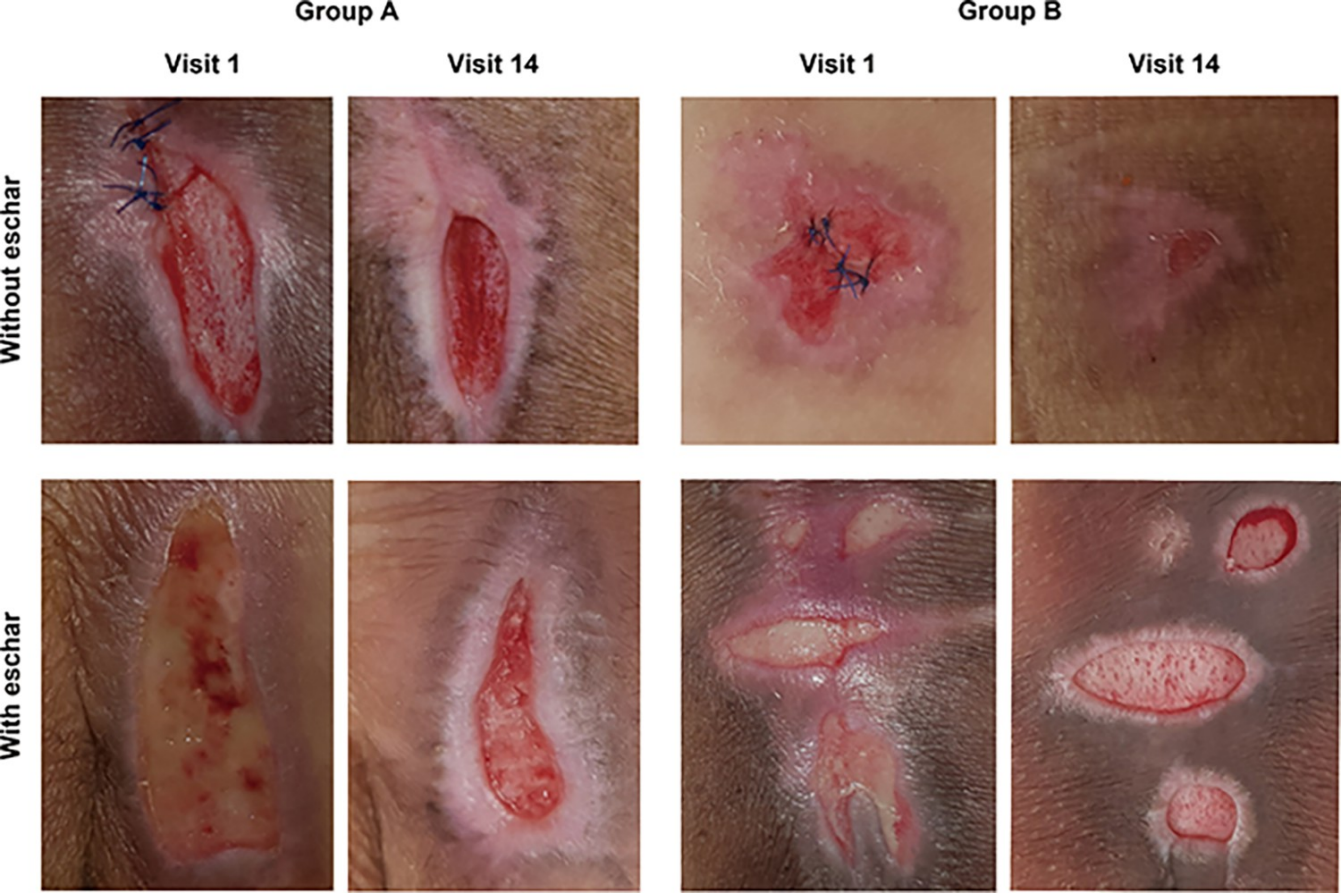
Clinical photographs between two groups. Clinical photographs between two groups according to with or without eschar. In the absence of eschar, it can be seen that LED represented a tendency of re-epithelialization and wound size reduction compared to Sham. All eschar in this investigation was white and slightly advanced from within the slough.

Sham had 15 wounds without eschar, whereas LED had nine wounds. At screening (visit 2), the average size (length × width) of the control wounds (Sham) was 5.24 ± 6.75 cm^2^, while the average size of the experimental wounds (LED) was 11.51 ± 13.46 cm^2^. Following 12 LED treatments (visit 14), Sham measured 0.96 ± 1.31 cm^2,^ while LED measured 0.56 ± 1.45 cm^2^. When the initial size of both groups was set to 100%, the post-treatment size of Sham was 13.80 ± 20.29%, and that of LED was 3.52 ± 6.68%. The change in wound size from pre-to post-treatment was significant in both groups (Sham, *p* < 0.001; LED, *p* < 0.001), but there was no significant difference between the two groups (*p* = 0.070) **(Table 7, Fig 4 and S2 Fig)**.

#### D. With eschar

There were 11 experimental wounds (LED) and three control wounds (Sham) at the deepest level. At screening (visit 2), the mean size (length × width) of the wounds in Sham was 3.91 ± 3.24 cm^2^, but the mean size in LED was 11.35 ± 17.01 cm^2^. Following 12 LED treatments (visit 14), Sham measured 0.48 ± 0.49 cm^2^, while LED measured 6.51 ± 9.46 cm^2^. LED demonstrated a significant difference in the wound size before and after treatment (*p* = 0.003), whereas Sham did not (*p* = 0.109); this may be due to Sham’s small number of wounds, which made obtaining statistically significant results challenging. In addition, there was no statistically significant difference in the treatment outcomes between the two groups (*p* = 0.148) **(Table 7, Fig 4)**.

### (3) The second validity assessment (Tissue biopsy)

**Fig 5:**
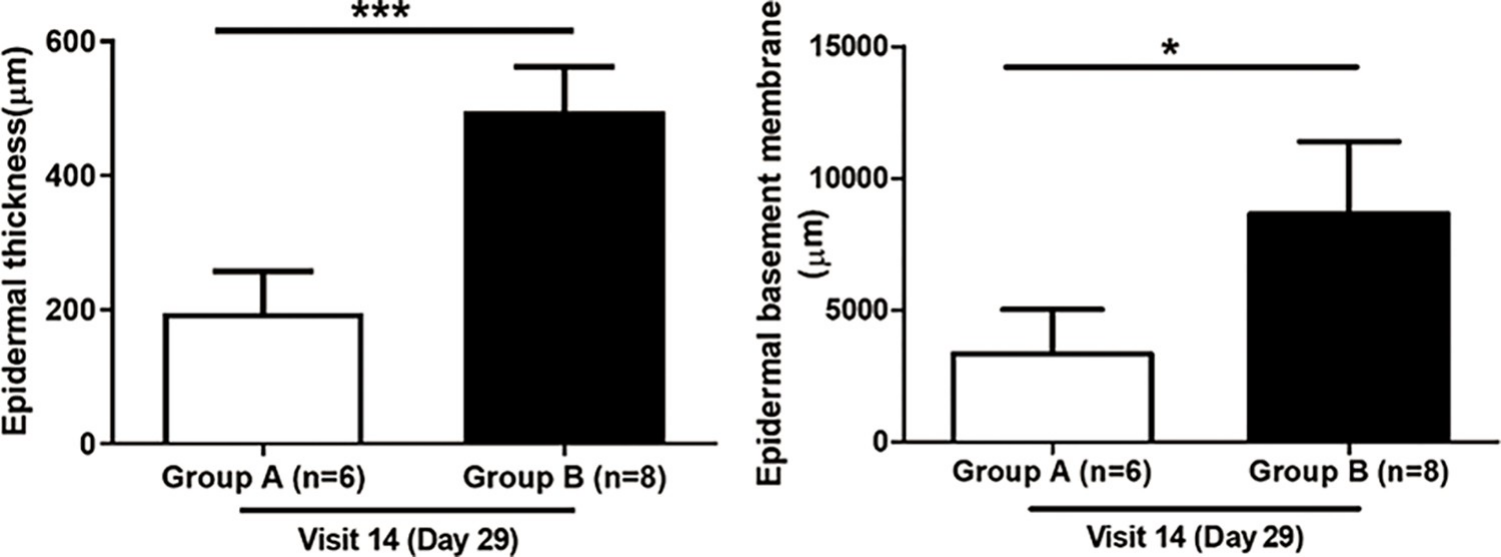
Analysis of epidermal regeneration using Hematoxylin and Eosin (H&E) staining. Scale bar : 500 μm. n, numbers. * : *p* < 0.05; *** : *p* < 0.001. **(Left)** The LED (495.62 ± 327.09 μm) was significantly increased the epidermal thickness compared to Sham (195.36 ± 263.04 μm) (*p* < 0.001). **(Right)** It was found that the basement membrane significantly increased in LED (8651.48 ± 7753.17 μm) compared to Sham (3338.14 ± 4132.74 μm) (*p* < 0.05).

In the analysis of epidermal thickness, LED (495.62 ± 327.09 μm) had significantly increased the epidermal thickness compared to Sham (195.36 ± 263.04 μm) (*p* < 0.001). Hematoxylin and eosin staining was also used to measure the basement membrane lengthening; it was found that the basement membrane significantly increased in LED (8651.48 ± 7753.17 μm) compared to Sham (3338.14 ± 4132.74 μm) (*p* < 0.05) **(Fig 5 and S3 Fig)**.

**Fig 6:**
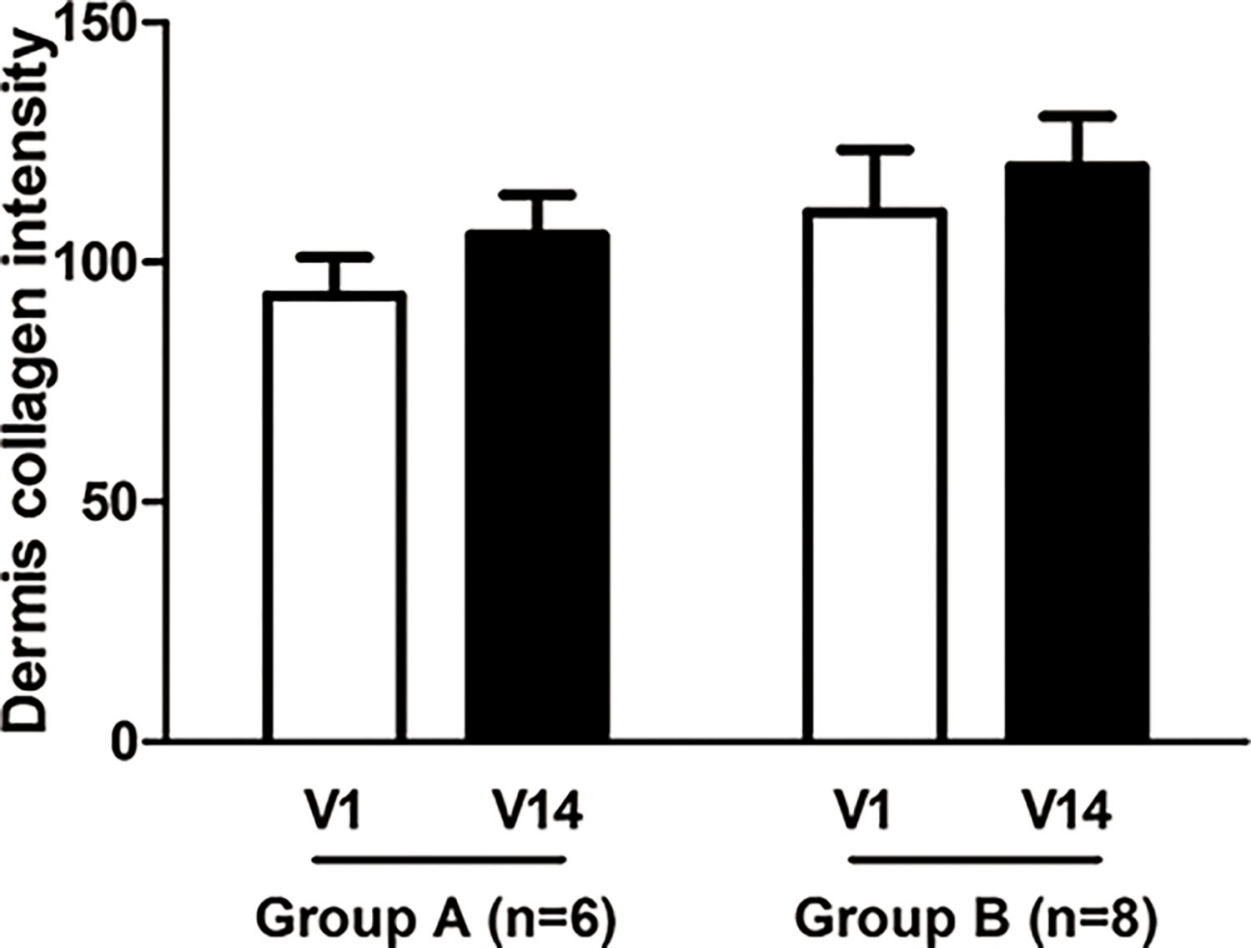
Analysis of collagen regeneration using Masson’s trichrome (MT) staining. Scale bar : 500 μm. V, visit; n, numbers. Though it was confirmed that the dermis collagen intensity was gradually increased in both Sham (visit 1; 93.07 ± 19.54, visit 14; 105.69 ± 20.49) and LED (visit 1; 110.45 ± 36.73, visit 14; 119.88 ± 29.79), there was no significant difference between the two groups (*p* = 0.112).

Though it was confirmed that the dermis collagen intensity was gradually increased in both Sham (visit 1; 93.07 ± 19.54, visit 14; 105.69 ± 20.49) and LED (visit 1; 110.45 ± 36.73, visit 14; 119.88 ± 29.79), there was no significant difference between the two groups (*p* = 0.112) **(Fig 6 and S4 Fig)**.

**Fig 7:**
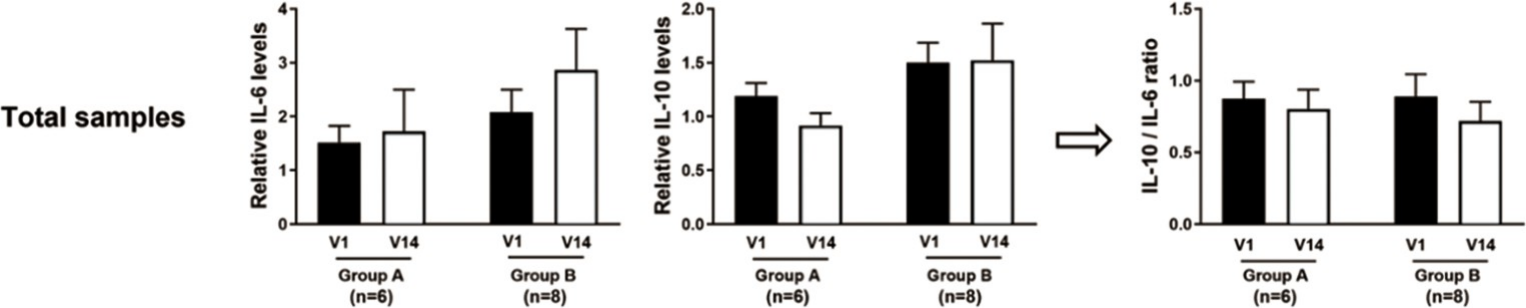
Relative mRNA expression of pro- and anti-inflammatory cytokines using quantitative real time polymerase chain reaction (qRT-PCR). V, visit; n, numbers; IL, interleukin. IL-6 slightly increased in both groups of total samples at visit 14 compared to visit 1, and IL-10 decreased in Sham while steady in LED. The IL-10 / IL-6 ratio was decreased in visit 14 compared to visit 1 in both groups, but all data was statistically insignificant.

In the quantitative real-time polymerase chain reaction, IL-6 showed a slight increase in both groups of total samples at visit 14 compared to visit 1. Meanwhile, in Sham, IL-10 decreased, while it remained stable in LED. Although the IL-10 / IL-6 ratio decreased at visit 14 compared to visit 1 in both groups, the difference was not statistically significant **(Fig 7 and S5 Fig)**.

**Fig 8:**
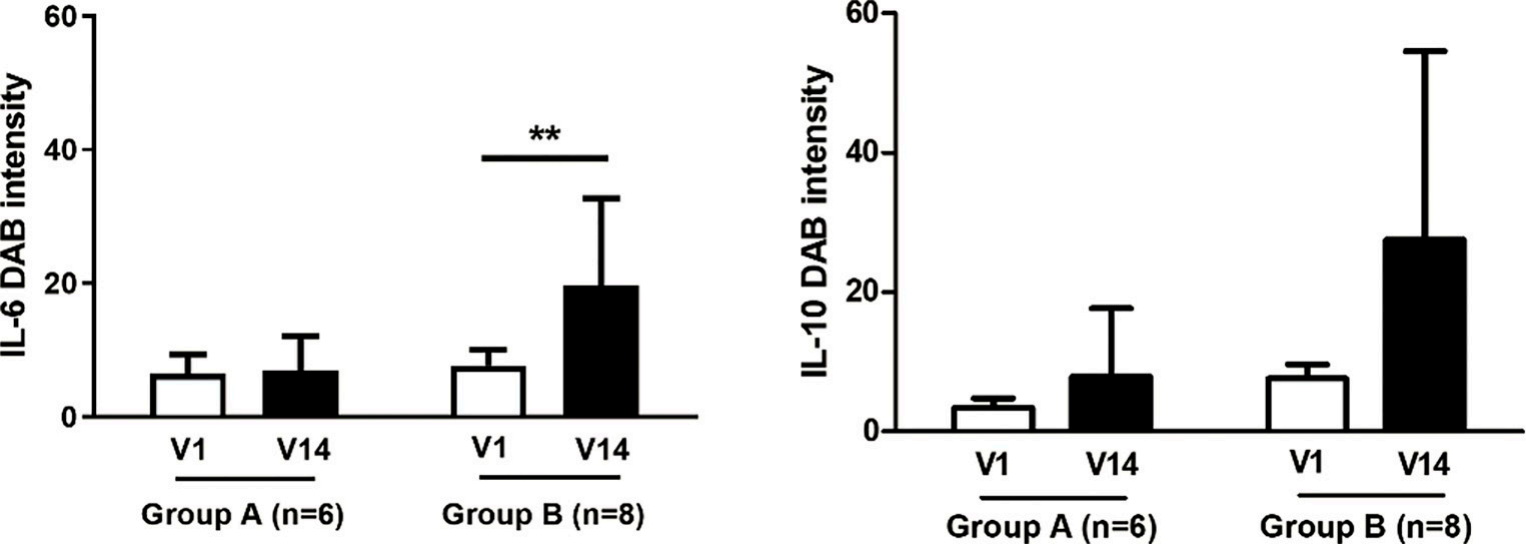
Protein expression of pro- and anti-inflammatory cytokines using immunohistochemistry. Scale bar : 500 μm; ** : *p* < 0.01. V, visit; n, number; IL, interleukin; DAB, diaminobenzidine. Protein-level analysis revealed an upward trend following the treatment of both IL-6 (Sham, visit 1; 6.43 ± 7.06, visit 14; 6.9 ± 5.20 vs. LED, visit 1; 9.32 ± 6.59, visit 14; 20.57 ± 13.10) and IL-10 (Sham, visit 1; 3.36 ± 3.45, visit 14; 7.87 ± 9.79 vs. LED, visit 1; 7.61 ± 5.84, visit 14; 30.52 ± 27.05). Particularly, there was a significant difference between the level of IL-6 elevated before and after treatment in LED (*p* < 0.01).

In immunohistochemistry, similarly to the ribonucleic acid-level findings, the protein-level analysis revealed an upward trend following the treatment of both IL-86 (Sham, visit 1; 6.43 ± 7.06, visit 14; 6.9 ± 5.20 vs. LED, visit 1; 9.32 ± 6.59, visit 14; 20.57 ± 13.10) and IL-10 (Sham, visit 1; 3.36 ± 3.45, visit 14; 7.87 ± 9.79 vs. LED, visit 1; 7.61 ± 5.84, visit 14; 30.52 ± 27.05). In particular, there was a significant difference in IL-6 levels before and after treatment in LED (*p* < 0.01) **(****Fig** 8 **and S6 Fig)**.

### (4) Safety assessment (Adverse reaction)

Throughout the 4-week treatment phase, 17 adverse events were documented among 15 participants—seven in the control group and ten in the experimental group. Notably, one participant faced severe adverse effects, prompting the study withdrawal. Among nine participants with moderate adverse events, five participants had legitimate isolation infections such as vancomycin-resistant enterococcus and COVID-19. A single adverse reaction was closely tied to the medical device. In total, 39.5% (15/38) of participants experienced adverse events; only 2.6% (1/38) of these were device-related, affirming its safety **(Table 10)**.

**Table 10:** Adverse events during clinical trial.

System of care criteria	Disease	Patient number (Group)	Severity	Relationship of medical device	Subsequent action
Blood and lymphatic system disorders	Pancytopenia	19 (LED)	Severe	No	Dropout
Cardiac disorders	Tachycardia	33 (Sham)	Moderate	No	Continue
General disorders and administration site conditions	Concomitant disease progression	19 (LED)	Severe	No	Dropout
	Fever	13 (LED), 25 (LED)	Mild	No	Continue
Infections and infestations	Corona virus infection	28 (LED)	Moderate	No	Dropout
	Bacteremia (Vancomycin resistant enterococcus)	10 (Sham), 12 (Sham), 18 (Sham)	Moderate	No	Dropout
Musculoskeletal and connective tissue disorders	Leg pain	5 (Sham)	Mild	No	Continue
Respiratory, thoracic, and mediastinal disorders	Pleural effusion	8 (LED)	Moderate	No	Continue
Skin and subcutaneous tissue disorders	Pruritus	31 (Sham)	Mild	No	Continue
	Redness	25 (LED)	Mild	No	Continue
Surgical and medical procedures	Percutaneous endoscopic gastrostomy	21 (Sham), 26 (LED)	Moderate	No	Continue
	Paracentesis abdomen	36 (LED)	Moderate	No	Continue
Miscellaneous	Pain	2 (LED)	Mild	**High**	Resume after one interruption (Visit 3)

Sham, Control group; LED, Experimental group

## Discussion

Diverse methodologies have been explored for the management of pressure ulcers due to their intricate underlying physiological mechanisms [1, 28, 29]. Notably, photobiomodulation therapy, including the use of LED, has garnered significant attention, particularly for its potential in promoting wound healing [7, 14, 30, 31]. Our investigation revealed that our device employing multispectral wavelengths exhibited a favorable impact on both epidermal thickness and the basement membrane, aligning with findings from prior research [32–35]. This treatment was also recognized for its capacity to augment collagen generation within the skin while concurrently suppressing the synthesis of matrix metalloproteinase [36]. Nevertheless, our study did not uncover any statistically significant distinction when compared to sham devices. This lack of significance can be attributed partly to variations in standardized parameters and a lack of consensus on the optimal wavelength and intensity settings among different studies. Furthermore, the degree of light transmission with and without eschar formation can vary, contributing to variability in results.

Eschar formation is a common feature of pressure ulcers. This phenomenon is a natural response to the body’s attempt to protect the underlying tissues from further damage and infection. Under a microscope, eschar typically consists of proteins, lipids, nucleic acids, and other cellular components denatured and coagulated by heat, pressure, or other factors. The presence of eschar can impede the healing of a pressure ulcer because it creates a barrier that prevents the growth of new tissue and hinders the delivery of oxygen and nutrients to the affected area [20, 37, 38]. In our study, the existence of an eschar, which signals a deeper lesion in the case of a second-degree pressure ulcer, restricted the penetration of LED light and lowered its efficiency by half; this is a shortcoming of a prospective, double-blind trial, and we suspect that the effect of LED therapy would have been more clearly defined if the patients had been selected and allocated.

The balance between the pro- and anti-inflammatory cytokines plays an important role in cell regeneration [39, 40]. Macrophage polarization is also an indicator of proliferation [14, 41]. In our study, when evaluating the ratio of IL-6 to IL-10 as an indicator of macrophage polarization, we did not observe consistent changes in the levels of these cytokines. This finding differs from the results reported in a study on the effectiveness of oral ulcers conducted by Wagner VP et al. [42]. The primary reason for the variability in the effectiveness of LEDs in treating pressure ulcers is the chronic nature of the wounds, which involves an ongoing inflammatory response. In chronic wounds, pressure ulcers are influenced by numerous factors that can affect LED treatment outcomes [8, 10]. These variables include underlying medical conditions, an individual’s immune response, and the overall wound-healing process [11, 28, 29]. All these factors contribute to the complexity and variability observed in the effectiveness of LED therapy for pressure ulcers.

**Fig 9:**
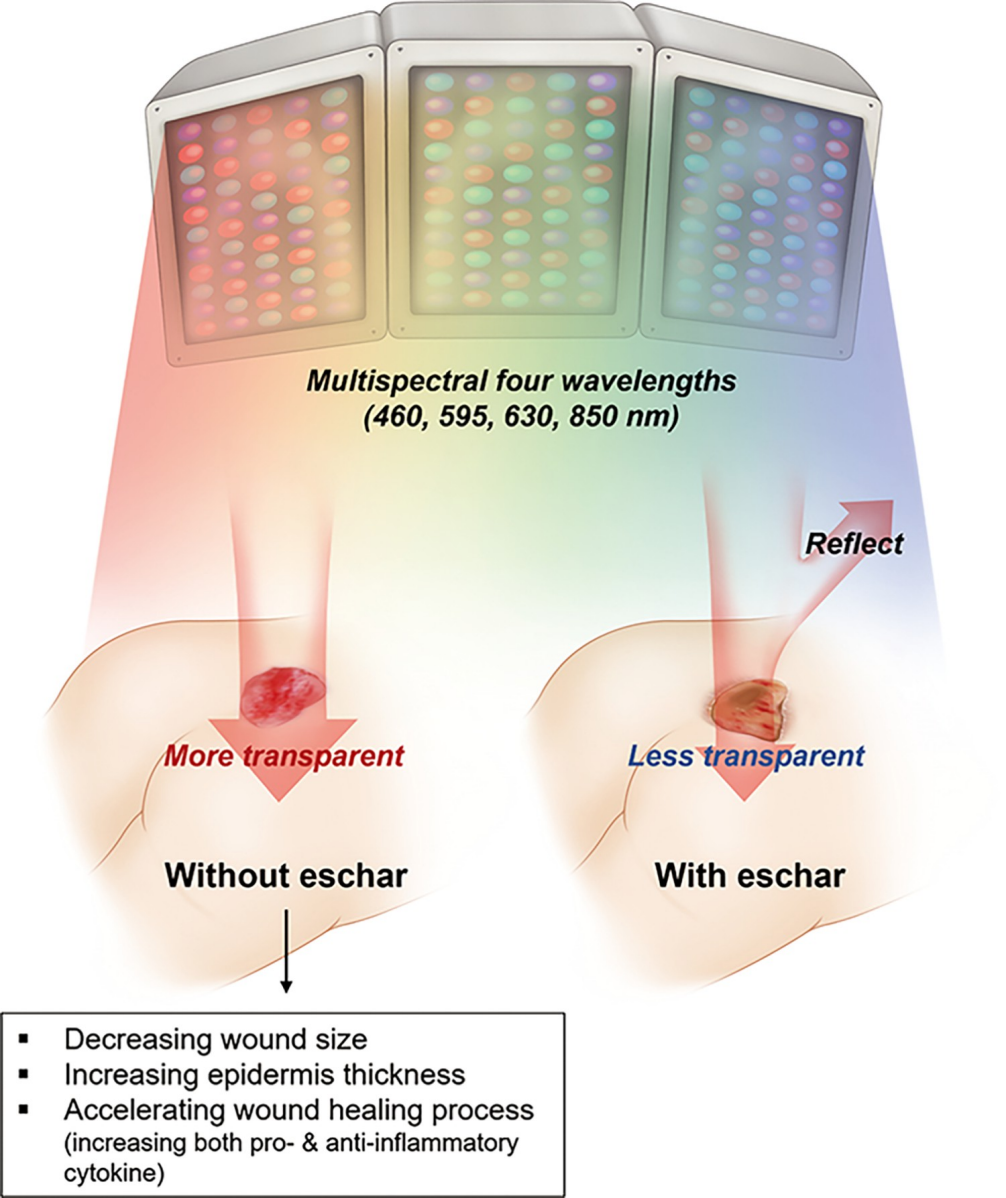
Scheme of hypothesis of multispectral light emitting diode treatment on low-grade pressure ulcer.

This prospective study has several limitations. First, using biopsies to study the effectiveness of LED treatment for pressure ulcers was that they only provide a limited, localized view of the wound. Biopsies involve sampling a small portion of the tissue, which may not fully represent the entire pressure ulcer. Clinical observations, on the other hand, provide a broader perspective by considering the overall response to LED treatment, including factors such as wound healing progress and symptom relief. Therefore, although biopsies were valuable for providing detailed cellular and molecular information, they have limitations in assessing the impact of LED therapy on the presence or absence of eschar in pressure ulcers. Second, to minimize bias in variables affecting pressure ulcers, a double-blinded prospective study had been designated. However, during the clinical trial, dropout occurred, and controlling for these variables arbitrarily was impossible because of prospective study. Consequently, differences in variables such as age, wound size, and the ratio of eschar occurrence arose between the two groups. Therefore, it is crucial to consider these limitations and analyze the results from different perspectives. Third, for the sham device, we developed a new parameter to implement the device visually identical to the therapeutic device by utilizing the minimum power required to emit LED light. While this energy did not induce changes in ambient temperature or warming of the tissue, it cannot be entirely ruled out that the energy generation itself may have some impact on the tissue. Finally, this study serves as an exploratory investigation and provides the groundwork for future confirmatory clinical trials. In our study, there was no evidence found to suggest that LED treatment was beneficial for grade 2 pressure ulcers. However, when the analysis was stratified by the presence or absence of eschar, a trend was observed suggesting that LED therapy might be beneficial in cases where eschar was absent. Based on these findings and the characteristics of the eschar, it is plausible that the eschar can increase the reflection of the LEDs and decrease the transmittance, thereby halving the therapeutic effect, and it can be assumed that the contribution of the LED treatment effect would be greater in the absence of eschar **(****Fig** 9**)**. However, additional research with a larger sample size should be done to acquire more robust and conclusive evidence regarding the effectiveness of LED treatment for pressure ulcers. The scenarios for power generation are presented in **S2 Table**.

## Conclusion

In summary, our investigation did not uncover supportive evidence for the efficacy of multispectral LED treatment in grade 2 pressure ulcers, with the presence of eschar formation emerging as a pivotal factor influencing recovery. Consequently, we advocate for a clinical trial involving a larger cohort of participants with grade 2 pressure ulcers without eschar to substantiate the efficacy of LED treatment.

## Supporting information

S1 Table Comorbidity diseases of patients. (DOCX)

S2 Table Calculation of sample size for confirmatory clinical trial. (DOCX)

S1 Fig The results of preclinical study using LED device. (A) A scheme of experimental design and four groups (B) Photographs of four groups according to time-serial change (C) A graph comparing wound sizes across the four groups. Among the four groups, the combined therapy (plasma + multispectral LED) was found to be most effective for infectious wound healing.
(JPG)

S2 Fig Wound area assessment graph between two groups. n, numbers; ** : *p* < 0.01; *** : *p* < 0.001. Graph of wound area assessment between two groups. At screening (visit 2), the average size (length × width) of the 18 lesions in the Sham was 5.02 ± 6.25 cm^2^, while the average size of the 20 lesions in the LED was 11.43 ± 15.12 cm^2^. After 12 LED treatments (visit 14), the size of the Sham was 0.88 ± 1.22 cm^2^ and the LED was 3.78 ± 7.54 cm^2^. When the initial size of both groups was set to 100%, the post-treatment size was 12.85 ± 18.74% for the Sham and 34.46 ± 20.84% for the LED. In both groups, there was a significant difference between the wound size before and after treatment (Sham, *p* < 0.001, and LED, *p* < 0.001), but there was no significant difference between the two groups (*p* = 0.061). The Sham had 15 wounds without eschar, while the LED had nine. At screening (visit 2), the average size (length × width) of the control wounds (Sham) was 5.24 ± 6.75 cm^2^, while the average size of the experimental wounds (LED) was 11.51 ± 13.46 cm^2^. Following 12 LED treatments (visit 14), the Sham measured 0.96 ± 1.31 cm^2^ while the LED measured 0.56 ± 1.45 cm^2^. When the initial size of both groups was set to 100%, the post-treatment size of the Sham was 13.80 ± 20.29% and that of the LED was 3.52 ± 6.68%. The change in wound size from pre-treatment to post-treatment was significant in both groups (Sham, *p* < 0.001, and LED, *p* = 0.001), but there was no significant difference between the two groups (*p* = 0.070). There were 11 experimental wounds (LED) and 3 control wounds (Sham) in deepest level. At screening (visit 2), the mean size (length × width) of the wounds in the Sham was 3.91 ± 3.24 cm^2^, but the mean size of the wounds in the LED was 11.35 ± 17.01 cm^2^. Following 12 LED treatments (visit 14), the Sham measured 0.48 ± 0.49 cm^2^, while the LED measured 6.51 ± 9.46 cm^2^. When the initial size of both groups was set to 100%, the post-treatment sizes of the Sham and B were 8.12 ± 8.06% and 59.77 ± 28.18%, respectively. The LED demonstrated a significant difference between the change in wound size before and after treatment (*p* = 0.003), whereas the Sham did not (*p* = 0.109). This may be due to the small number of wounds in the Sham, which made obtaining statistically significant results challenging. In addition, there was no statistically significant difference in treatment outcomes between the two groups (*p* = 0.148).
(TIF)

S3 Fig Microscope images of epidermal regeneration using Hematoxylin and Eosin (H&E) staining between two groups. **(A)** The thickness of the epidermis was calculated as the mean of the shortest, longest, and middle sites. **(B)** Microscopic images of basement membrane lengthening (red line).
(TIF)

S4 Fig Microscopic images of collagen deposition with blue color in MT staining between two groups. Quantification of the collagen fibers was counted with the software of Image J as red density.
(TIF)

S5 Fig Relative mRNA expression of pro- and anti-inflammatory cytokines using Quantitative real time polymerase chain reaction (qRT-PCR) according to with and without eschar. V, visit; n, numbers; IL, interleukin. **(A)** Without eschar. **(B)** With eschar. Interestingly, both IL-6 and IL-10 tended to increase after treatment in the classification without eschar, where LED treatment effects were deemed to be relatively high.
(TIF)

S6 Fig Protein expression of pro- and anti-inflammatory cytokines using immunohistochemistry. Stained sections were viewed with a microscope as brown color, and the processed images were analyzed for using Image J which converted to the red density.
(TIF)

S1 File CONSORT checklist. (DOC)

S2 File Institutional review board approval certification. (PDF)

S3 File Institutional review board approval protocol 1 (Korean). (PDF)

S4 File Institutional review board approval protocol 1 (English). (PDF)

S5 File Institutional review board approval protocol 2 (Korean). (PDF)

S6 File Institutional review board approval protocol 2 (English). (PDF)

S7 File Institutional review board initial notice (Korean). (PDF)

S8 File Institutional review board initial notice (English). (PDF)

S9 File Institutional review board interim notice (Korean). (PDF)

S10 File Institutional review board interim notice (English). (PDF)

S11 File Institutional review board exit notice (Korean). (PDF)

S12 File Institutional review board exit notice (English). (PDF)

S13 File Korea Ministry of Food and Drug Safety approval certification 1 (Korean). (PDF)

S14 File Korea Ministry of Food and Drug Safety approval certification 1 (English). (PDF)

S15 File Korea Ministry of Food and Drug Safety approval certification 2 (Korean). (PDF)

S16 File Korea Ministry of Food and Drug Safety approval certification 2 (English). (PDF)
