# Supplementary material for: The validity and safety of multispectral light emitting diode (LED) treatment on grade 2 pressure ulcer: Double-blinded, randomized controlled clinical trial
Source: PLoS One. 2024 Aug 23;19(8):e0305616. doi: 10.1371/journal.pone.0305616 (PMC11343461; doi:10.1371/journal.pone.0305616)
Supplement: S1 Table — (DOCX) [file pone.0305616.s001.docx]

**Supplement 1. Comorbidity diseases of patients**

| System of care criteria | Sham | LED |
| --- | --- | --- |
| Cardiac disorder |  |  |
|  | Atrial fibrillation | Acute myocardial infarction |
|  | Cardiac valve disease | Coronary artery disease |
|  | Angina pectoris |  |
| Endocrine disorder |  |  |
|  |  | Hypothyroidism |
| Gastrointestinal disorder |  |  |
|  | Dysphagia | Hemoperitoneum |
|  | Pancreatic necrosis | Duodenal ulcer perforation |
|  | Ileus |  |
| General disorders and administration site conditions |  |  |
|  |  | Stent embolization |
| Hepatobiliary disorders |  |  |
|  |  | Cirrhosis alcoholic |
| Infections and infestations |  |  |
|  | Tuberculosis | Infective spondylitis |
|  | Peritonitis |  |
|  | Empyema |  |
|  | Intervertebral discitis |  |
| Injury, poisoning and procedural complications |  |  |
|  | Toxicity to various agents | Femur fracture |
|  | Femur fracture | Mallet finger |
|  | Vascular pseudoaneurysm | Clavicle fracture |
|  | Lower limb fracture | Lumbar vertebral fracture |
|  | Traumatic liver injury | Spinal cord injury cervical |
|  | Pancreatic injury | Subdural hemorrhage |
|  | Abdominal injury | Cervical vertebral fracture |
|  | Spinal cord injury | Thoracic vertebral fracture |
|  | Pelvic fracture | Fibula fracture |
|  | Post laminectomy syndrome | Tibia fracture |
|  |  | Epidural hemorrhage |
|  |  | Brain contusion |
|  |  | Skull fractured base |
|  |  | Pneumocephalus |
| Investigations |  | Unusual laboratory result |
| Metabolism and nutrition disorders |  |  |
|  | Diabetes mellitus | Diabetes mellitus |
|  | Dyslipidemia |  |
| Musculoskeletal and connective tissue disorders |  |  |
|  | Osteoporosis | Thoracic spinal stenosis |
|  | Spondyloarthropathy | Arthralgia |
|  |  | Neuropathic arthropathy |
| Neoplasms benign, malignant, and unspecified |  |  |
|  | Plasma cell myeloma | Renal cancer |
|  |  | Neurogenic tumor |
|  |  | Breast cancer |
| Nervous system disorders |  |  |
|  | Dementia | Subarachnoid hemorrhage |
|  | Cerebrovascular accident | Cerebral hemorrhage |
|  | Parkinson’s disease | Dementia |
|  | Cervical cord compression | Hepatic encephalopathy |
|  | Cerebral infarction | Spinal epidural hemorrhage |
| Psychiatric disorders |  |  |
|  | Major depression | Affective disorder |
|  |  | Mental disorder |
|  |  | Alcoholism |
|  |  | Bipolar disorder |
|  |  | Schizophrenia |
| Renal and urinary disorders |  |  |
|  | Chronic kidney disease | Acute kidney injury |
|  |  | End stage renal disease |
| Reproductive system and breast disorders |  |  |
|  | Benign prostatic hyperplasia |  |
| Respiratory, thoracic, and mediastinal disorders |  |  |
|  |  | Pneumonia aspiration |
| Surgical and medical procedures |  |  |
|  | Hip arthroplasty | Closed fracture manipulation |
|  | External fixation of fracture | Arthrodesis |
|  | Vertebroplasty | Open reduction of fracture |
|  | Dialysis | Spinal laminectomy |
|  | Abscess drainage | Spinal decompression |
|  | Gastrostomy | Intervertebral disc operation |
|  | Debridement | Hip arthroplasty |
|  | Skin graft | Percutaneous coronary intervention |
|  | Hemodialysis | Thyroidectomy |
|  | Spinal corpectomy | Ventricular drainage |
|  | Leg amputation | Omentoplasty |
| Vascular disorders |  |  |
|  | Hypertension | Hypertension |
