# Supplementary material for: The validity and safety of multispectral light emitting diode (LED) treatment on grade 2 pressure ulcer: Double-blinded, randomized controlled clinical trial
Source: PLoS One. 2024 Aug 23;19(8):e0305616. doi: 10.1371/journal.pone.0305616 (PMC11343461; doi:10.1371/journal.pone.0305616)
Supplement: S2 Table — (DOCX) [file pone.0305616.s002.docx]

**Supplement 2. Calculation of sample size for confirmatory clinical trial**

|  | **Reference factor** | **The number of groups** | **Total numbers** | **Target power** | **Actual power** | **Alpha** |
| --- | --- | --- | --- | --- | --- | --- |
| Scenario 1 | Wound size (cm^2^) in total | 2 | 254 | 0.90 | 0.90165 | 0.050 |
| Scenario 2 | The rate of wound size (%) in total | 2 | 160 | 0.90 | 0.90300 | 0.050 |
| Scenario 3 | Wound size (cm^2^) in without eschar | 2 | 102 | 0.90 | 0.90399 | 0.050 |
| Scenario 4 | The rate of wound size (%) in without eschar | 2 | 312 | 0.90 | 0.90146 | 0.050 |
