# Supplementary figures and images for: The validity and safety of multispectral light emitting diode (LED) treatment on grade 2 pressure ulcer: Double-blinded, randomized controlled clinical trial
Source: PLoS One. 2024 Aug 23;19(8):e0305616. doi: 10.1371/journal.pone.0305616 (PMC11343461; doi:10.1371/journal.pone.0305616)

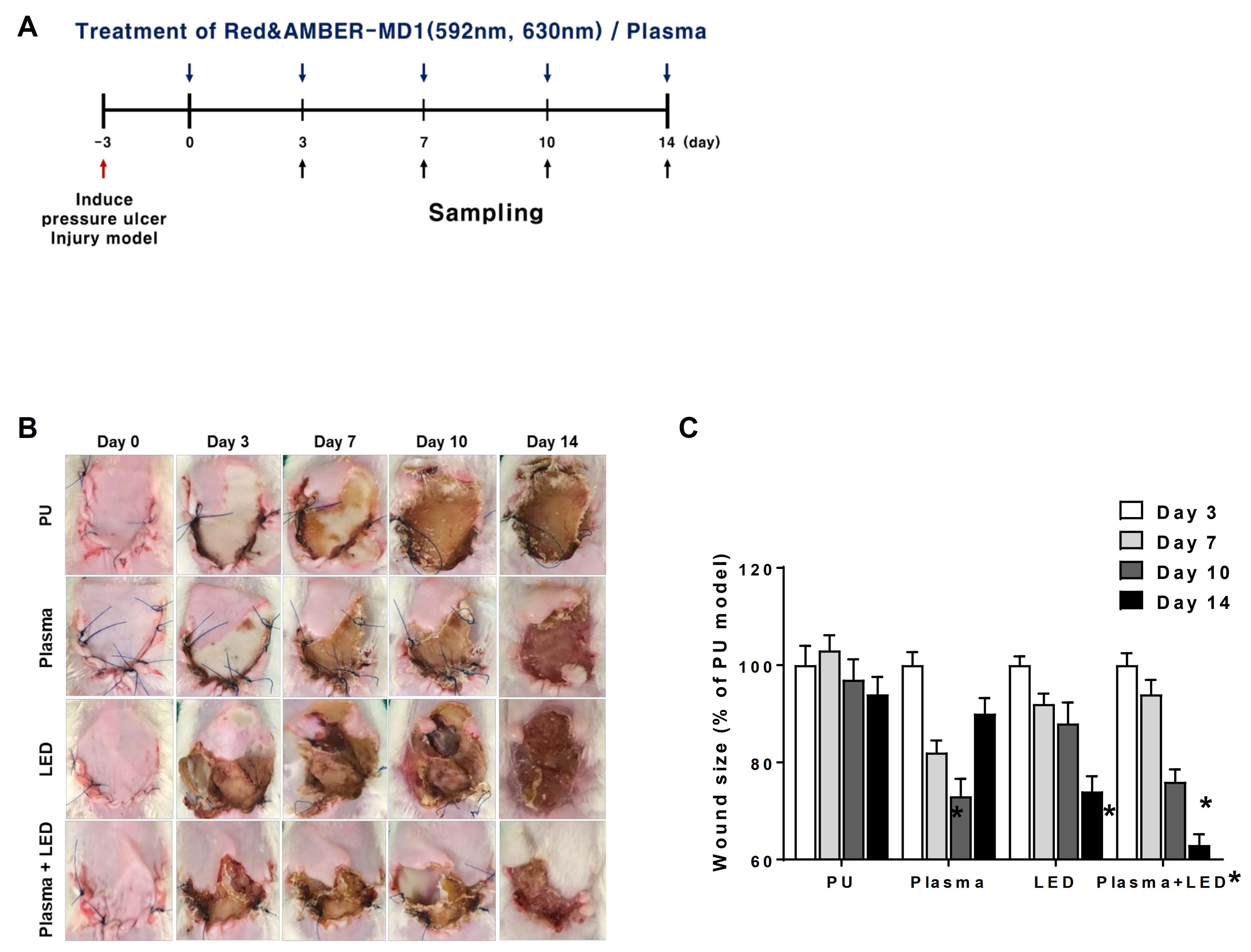

Supplement: S1 Fig — (A) A scheme of experimental design and four groups (B) Photographs of four groups according to time-serial change (C) A graph comparing wound sizes across the four groups. Among the four groups, the combined therapy (plasma + multispectral LED) was found to be most effective for infectious wound healing. (JPG) [file pone.0305616.s003.jpg]
