# Supplementary material for: The validity and safety of multispectral light emitting diode (LED) treatment on grade 2 pressure ulcer: Double-blinded, randomized controlled clinical trial
Source: PLoS One. 2024 Aug 23;19(8):e0305616. doi: 10.1371/journal.pone.0305616 (PMC11343461; doi:10.1371/journal.pone.0305616)
Supplement: S2 File — (PDF) [file pone.0305616.s010.pdf]

DANKOOK UNIVERSITY HOSPITAL

단국대학교병원

# DANKOOK UNIVERSITY HOSPITAL INSTITUTIONAL REVIEW BOARD

TEL: 82-41-550-7491
FAX: 82-41-550-7499

Dankook Univ. Hospital 201, Manghyang-ro,,
Dongsam-gu, Cheonan-si Chungcheongnam-do,
31116, Korea

# Certificate of Approval

Board Action Date : Sep 16, 2020
Study No. : Oct 28, 2020
Estimated Study Period : Oct 28, 2020 ~ Oct 26, 2022
IRB No. : DKUH 2020-09-014

The followings were approved :

Investigator : Nam Kyu Lim, MD.

Protocol Number : Ver. 2.0 ~ Ver. 4.0

Research Project Title :

The validity and safety of multispectral light emitting diode (LED) treatment on grade 2 pressure ulcer: double-blinded, randomized controlled clinical trial

Approved document list

- Protocol
- Case Report Form
- Declaration of Research Ethics
- Informed Consent Form

*This research have obtained an DKUH IRB-approved waiver of informed consent, since this research involves no greater than minimal risk to participants, and the waiver will not adversely affect the rights and welfare of the participants.
*All conditions of approval previously established by DKUH IRB for this research project continue to apply.

If you have any questions, contact DKUH IRB at 82-41-550-7491

This is to certify that the information contained herein is true and correct as reflected in the records of the DKUH Institutional Review Board(DKUH IRB)

WE CERTIFY THAT DKUH IRB IS IN FULL COMPLIANCE WITH GOOD CLINICAL PRACTICES AS DEFINED UNDER THE MINISTRY OF FOOD AND DRUG SAFETY(MFDS) REGULATIONS AND THE INTERNATIONAL CONFERENCE ON HARMONISATION(ICH) GUIDELINES.

Chairperson : [signature]　　　　Date : 2023.10.19.

ALL DKUH IRB APPROVED INVESTIGATORS MUST COMPLY WITH THE FOLLOWING :

1. Conduct the research as required by the protocol.
2. Use only the Consent Form bearing the DKUH IRB " APPROVED" stamp.
3. Provide non-Korean speaking subjects with a certified translation of the sapproved Consent Form in the subject's first language. The translated version must be approved by the DKUH IRB.
4. Obtain pre-approval from the DKUH IRB of any changes in the research activity (except when necessary to protect human subjects; immediately report to the DKUH IRB any such emergency changes for the protection of human subjects).
5. Report to the DKUH IRB the death, hospitalization, or serious illness of any study subject.
6. Promptly report to the DKUH IRB any new information that may adversely affect the safety of the subjects or the conduct of the trial.
7. Provide reports to the DKUH IRB concerning the progress of the research, when requested.
8. Obtain pre-approval of study advertisements from the DKUH IRB before use.
9. Conduct the informed consent process without coercion or undue influence, and provide the potential subject sufficient opportunity to consider whether or not to participate.

Korea FDA regulations require that the DKUH IRB conduct review of approved research. You will receive Continuing Review Report forms from the DKUH IRB. These reports must be returned even though your study may not have started.

DISTRIBUTION OF COPIES:

SPONSOR:

CRO:

OTHER:

INSTITUTION:

This is to certify that the information contained herein is true and correct as reflected in the records of the DKUH Institutional Review Board. **We certify that DKUH IRB is in full compliance with Good Clinical Practice as defined under the Korea Food and Drug Administration(KFDA) regulations and the International Conferenceon Harmonisation(ICH) guidelines.**
