# Supplementary material for: The validity and safety of multispectral light emitting diode (LED) treatment on grade 2 pressure ulcer: Double-blinded, randomized controlled clinical trial
Source: PLoS One. 2024 Aug 23;19(8):e0305616. doi: 10.1371/journal.pone.0305616 (PMC11343461; doi:10.1371/journal.pone.0305616)
Supplement: S3 File — (PDF) [file pone.0305616.s011.pdf]

안전한 식의약, 건강한 국민, 행복한 사회

# 식 품 의 약 품 안 전 처

수신자 ㈜링크옵틱스, 대표:최용원 귀하 (우61009 광주광역시 북구 첨단벤처로 16번길 3)

(경유)

제목 의료기기 임상시험계획 승인[㈜링크옵틱스-2등급의료용조합자극기(MD-032M)]

---

1. 귀사에서 우리 처에 신청(접수번호: 20200076830, 2020.06.25)하신 임상시험계획 승인 "2등급의료용조합자극기(MD-032M)"와 관련됩니다.

2. 위 건을 검토한 결과, 「의료기기법 시행규칙」 제20조제2항에 따른 의료기기 임상시험계획이 타당한 것으로 판단되어, 같은 법 시행규칙 제20조제4항의 규정에 따라 붙임과 같이 승인하오니, 「의료기기법」 제10조 및 같은 법 시행규칙 제24조제1항(별표2의 시설과 제조 및 품질관리체계의 기준에 적합한 의료기기 사용 포함)과 별표3「의료기기 임상시험 관리기준」,같은 법 시행규칙 제42조 및 제43조제2항을 준수하여 임상시험을 실시하시기 바랍니다.

3. 아울러, 동 임상시험은 별표3「의료기기 임상시험 관리기준」제7호마목1)에 따라 식약처장 및 해당 임상시험기관 심사위원회에서 승인한 임상시험계획서를 준수하여 실시하시기 바라며, 시행규칙 제24조제2항의 규정에 따라 임상시험실시상황을 별지 제25호 서식(의료기기임상시험실시상황보고서)으로 매년 2월말까지, 임상시험을 종료한 때에는 별지 제26호 서식(의료기기임상시험종료보고)으로 종료 후 20일 이내에 우리처(의료기기정책과)에 보고하여야 합니다.

4. 또한, 유전자 검사를 하고자 하는 경우에는「생명윤리 및 안전에 관한 법률」제49조에 따라 보건복지부령이 정하는 시설 및 인력 등을 갖추고 보건복지부 장관에게 신고한 검사기관에 의뢰하시기 바라며, 동 법령의 관련 규정을 준수하시기 바랍니다.

5. 끝으로, 의료기기 임상시험계획(변경포함)승인과 동 제품의 허가여부는 별개로 향후 동 제품의 허가 신청 시 의료기기 법규에 따라 허가여부를 재검토하게 됨을 알려드립니다.

붙임 : 의료기기 임상시험계획 승인서 1부(온라인수령). 끝.

# 식 품 의 약 품 안 전 처 장

연구위원 **홍정훈** 주무관 **이희성** 의료기기 기준 · 정보화팀장 전결 07.23 **성홍모**

시행 의료기기정책과-MFDS-4754 (2020.07.23) 접수 20200076830 (2020.06.25)

우 28159 충청북도 청주시 흥덕구 오송읍 오송생명2로 187 식품의약품안전처 의료기기기준정보화팀 /
전화 043-719-5667 전송 043-719-5650 / jaspers74@korea.kr /
