# Supplementary material for: The validity and safety of multispectral light emitting diode (LED) treatment on grade 2 pressure ulcer: Double-blinded, randomized controlled clinical trial
Source: PLoS One. 2024 Aug 23;19(8):e0305616. doi: 10.1371/journal.pone.0305616 (PMC11343461; doi:10.1371/journal.pone.0305616)
Supplement: S4 File — (PDF) [file pone.0305616.s012.pdf]

Food and Drug Administration

식품의약품안전처

Recipient Link Optics, CEO: Yongwon Choi You (3, 16th Avenue, Buk-gu, Gwangju, Korea, 61009) 첨단벤처로 16번길 3)

(via)

Titles Medical Devices Clinical trial plan Approval [Link Optics, Inc. Class 2 Medical Combination Stimulator (MD-032M)]

1. This is related to the protocol approval "Class 2 Medical Combination Stimulator (MD-032M)" that your company applied to us (application number: 20200076830, June 25, 2020).

2. After reviewing the above case, we have determined that the medical device clinical trial plan pursuant to Article 20(2) of the Enforcement Rules of the Medical Device Act is valid, and we approve it as attached in accordance with the provisions of Article 20(4) of the Enforcement Rules of the same Act. Please conduct the clinical trial in accordance with Article 10 of the Medical Device Act and Article 24(1) of the Enforcement Rules of the same Act (including the use of medical devices that meet the standards of facilities and manufacturing and quality management systems in Appendix 2), Appendix 3 "Medical Device Clinical Trial Management Standards," and Articles 42 and 43(2) of the Enforcement Rules of the same Act.

3. In addition, the clinical trial shall be conducted in accordance with the clinical trial protocol approved by the head of the Ministry of Food and Drug Safety and the relevant clinical trial site review committee in accordance with Article 7, item 1) of Appendix 3 of the 「Medical Device Clinical Trial Management Standards」, and the clinical trial status shall be reported to the Ministry (Medical Device Policy Division) by the end of February each year in the form of Appendix 25 (Medical Device Clinical Trial Status Report) and within 20 days after the end of the clinical trial in the form of Appendix 26 (Medical Device Clinical Trial Termination Report) in accordance with the provisions of Article 24, paragraph 2 of the Enforcement Rules.

4. In addition, if you wish to have a genetic test, please refer to a laboratory that has been notified to the Minister of Health and Welfare in accordance with Article 49 of the Act on Bioethics and Safety, and has the facilities and personnel specified by the Ministry of Health and Welfare Ordinance, and comply with the relevant provisions of the Act.

5. Finally, we would like to inform you that the approval of a medical device clinical trial plan (including changes) is separate from the approval of the product, and the approval of the product will be reviewed in accordance with the Medical Device Act when the product is applied for approval in the future.

Attachment : 1 copy of the medical device protocol approval form (available online). End.

Expression品 of 약 품 處n before chapte r

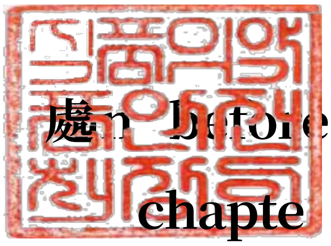

Research Fellow **Jung Hoon Hong**

Hee Sung Lee

Head of Medical Device Standards and Informatics 全決07.23 **Scarlet t**

Medical Device Organization Enforcement (2020.07.23) Submissions 20200076830 (June 25, 2020)

-MFDS-4754

Woo 28159 187, Osong-saeng 2-ro, Osong-eup, Heungdeok-gu, Cheongju-si, Chungcheongbuk-do, Korea /
Tel: +82-43-719-5667 Send to 043-719-5650/ jaspers74@korea.kr /
fax.
