# Supplementary material for: The validity and safety of multispectral light emitting diode (LED) treatment on grade 2 pressure ulcer: Double-blinded, randomized controlled clinical trial
Source: PLoS One. 2024 Aug 23;19(8):e0305616. doi: 10.1371/journal.pone.0305616 (PMC11343461; doi:10.1371/journal.pone.0305616)
Supplement: S5 File — (PDF) [file pone.0305616.s013.pdf]

시점확인필
진본
2020-07-23
15:18:53
KST
정부시점확인센터

제 1104 호

의료기기 임상시험계획승인서

신 청 인<br>(대표자)	성 명	최용원	생년월일	1968년 07월 16일
	주 소	광주광역시 북구 첨단벤처로16번길 3 본동 1층		
제조(수입)<br>업 소	명칭(상호)	(주)링크옵틱스	업허가번호	제 4345 호
	소 재 지	광주광역시 북구 첨단벤처로16번길 3		
제 조 원<br>(수입 또는 제조공정 전부 위탁의 경우)	명칭(상호)		제 조 국	
	소 재 지			
임 상 시 험<br>개 요	명칭(제품명, 품목명, 모델명)	2등급의료용조합자극기	분류번호(등급)	A16270.01(2)
	임상시험계획 승인번호	제 1104 호		
	임상시험의 제목	경도 욕창 이환 환자를 대상으로, 의료용 광선 조사기 BELLALUX Lite의 창상 회복에 대한 안전성 및 유효성을 평가하기 위해, 단일기관, 이중 눈가림, 무작위배정, 평행설계(sham기기 대조)의 전향적 탐색 임상시험		

「의료기기법」 제 10조 및 같은 법 시행규칙 제20조제4항에 따라 위와 같이 임상시험계획을 승인합니다.

※ 붙임 : 의료기기 임상시험계획승인신청서 1부.

2020 년 07 월 23 일

식 품 의 약 품 안 전 처 장 (인)

식품의약품안전처장인

※ 본 증명서는 인터넷으로 발급되었으며, 홈페이지(emed.mfds.go.kr)의 발급문서진위확인 메뉴를 통해 위변조 여부를 확인할 수 있습니다.
또한, 문서하단의 바코드로도 진위확인(스캐너용 문서확인프로그램)을 하실 수 있습니다.

(뒤 쪽)

변경 및 처분 사항 등	
년 월 일	내 용
2020-07-23	최초승인

모델명(형명)	
일련번호	형 명
1	MD-032M

포장단위	
일련번호	포장단위
1	set

식품의약품안전처

※ 본 증명서는 인터넷으로 발급되었으며, 홈페이지(emed.mfds.go.kr)의 발급문서진위확인 메뉴를 통해 위변조 여부를 확인할 수 있습니다.
또한, 문서하단의 바코드로도 진위확인(스캐너용 문서확인프로그램)을 하실 수 있습니다.
