# Supplementary material for: The validity and safety of multispectral light emitting diode (LED) treatment on grade 2 pressure ulcer: Double-blinded, randomized controlled clinical trial
Source: PLoS One. 2024 Aug 23;19(8):e0305616. doi: 10.1371/journal.pone.0305616 (PMC11343461; doi:10.1371/journal.pone.0305616)
Supplement: S6 File — (PDF) [file pone.0305616.s014.pdf]

No. 1104

# Medical Device IND

| | | | | |
|---|---|---|---|---|
| Applicant (as a representative) | Name | Yongwon Choi | Date of birth | July 16, 1968 |
| | Location | 3, 16beon-gil, Cheonan Venture-ro, Buk-gu, Gwangju, Korea | | |
| Manufacturing (importing) establishments | Name (business name) | Link Optics, Inc. | License number | No. 4345 |
| | Location | 3, 16beon-gil, Cheoncheon Venture-ro, Buk-gu, Gwangju, Korea | | |
| Country of manufacture (if importing or outsourcing the entire manufacturing process) | Name (business name) | | Country of Manufacture | |
| | Location | | | |
| Clinical trials overview | Name (product name, item name, model name) | Class 2 Medical Combination Stimulator | Classification number (rating) | A16270.01(2) |
| | Protocol approval number | No. 1104 | | |
| | Title of the study | A single-center, double-blind, randomized, parallel-group, prospective exploratory trial to evaluate the safety and efficacy of the medical light irradiator BELLALUX Lite on wound healing in patients with mild pressure ulcer co-morbidity (sham device control) | | |
| "Pursuant to Article 10 of the Medical Device Act and Article 20, Paragraph 4 of the Enforcement Rules of the same Act, we approve the clinical trial plan as above. | | | | |

| |
|---|
| * Attachment: 1 copy of the medical device clinical trial plan approval application.<br><br>July 23, 2020<br><br>**Ministry of Food and Drug Safety** |

(back)

| Changes and dispositions, etc. | |
|---|---|
| Year Month Day | Contents |
| July 23, 2020 | Initial Authorization |

| Model Name (Type Name) | |
|---|---|
| Serial Number | Type name |
| 1 | MD-032M |

| Packaging Units | |
|---|---|
| Serial Number | Packaging Units |
| 1 | Set |
