# Supplementary material for: The validity and safety of multispectral light emitting diode (LED) treatment on grade 2 pressure ulcer: Double-blinded, randomized controlled clinical trial
Source: PLoS One. 2024 Aug 23;19(8):e0305616. doi: 10.1371/journal.pone.0305616 (PMC11343461; doi:10.1371/journal.pone.0305616)
Supplement: S8 File — (PDF) [file pone.0305616.s016.pdf]

DeepL
Subscribe to DeepL Pro to translate larger documents.
Visit www.DeepL.com/pro for more information.

Notices

The document retention period for this assignment is 3 years.						
Receive	Referral (support) organizations	Department of Health and Human Services				
	Principal Investigator	Namkyu Lim, Plastic Surgery				
IRB File No.		DKUH 2020-09-014-002	Review Content	Corrective Action Plan	Notification Date	October 28, 2020
Project name	Korean	A single-center, double-blind, randomized, parallel-group, prospective exploratory clinical trial to evaluate the safety and efficacy of the medical light irradiator BELLALUX Lite on wound healing in patients with mild pressure ulcer co-morbidity.				
	English					
Study Code			Study Nick Name			

Research Category1	☐ Medications ☐ Biologics ☐ Cellular Therapeutics ☐ Nutraceuticals
	☐ Medical Procedures ■ Medical Devices (◯ Class 1 ◉ Tier 2 ◯ Tier 3 ◯ Level 4)
	☐ Not applicable
Research Category2	■ Human Subjects Research ☐ Human remains (specimen) research ☐ Medical Records Research
	☐ Genetic Resources ☐ Gene Therapy
	☐ Embryology ☐ Somatic cell cloning embryo research ☐ Stem Cell Line Research

This form was issued electronically (PDF file).
Electronic forms without a barcode cannot be verified for authenticity with the verification-only viewer and will not be marked as authentic.

	☐ Other ( )						
Research Category3	◉ Prospective studies		○ Retrospective studies		○ Prospective & Retrospective Parallel Studies		
Research Category 4	☐ Interventional Studies		☐ Surveys		☐ Data analysis and research		
	■ Observational studies (		☐ cross-sectional study		■ Patient-controlled studies ☐ Cohort Studies)		
	☐ Other ( )						
Research Category 5	☐ Studies not involving human subjects Non-clinical study (in vitro. in vivo preclinical study)						
Common name				Product name			
Total Subject Cases	All	38 people	Domestic	38 people	Headquarters	38 people	
Study approval period	October 28, 2020 - October 27, 2021						
Referral sources	Organization Name	Department of Health and Human Services	Representative (position)		First Name		
List of Submission Documents	(Attachment) Research Plan [2.0] [07／Oct／20]<br>(Attachment) Informed Consent Form [2.0]<br>[07／Oct／20] (Attachment) Report 1 [] [1]<br>(attached) report 2 [] []<br>(Attachment) Human Derivatives<br>Research Consent Form [1.0] []<br>(Attachment) Change Contingency<br>Table [2.0] [05／Oct／20].<br>(Attachment) Application for Corrective Review [2.0] [05／Oct／20]						
Related Rationale	Evaluation Date	October 28, 2020					
When to report	Until August 27, 2021		Remar				

This form was issued electronically (PDF file).
Electronic forms without a barcode cannot be verified for authenticity with the verification-only viewer and will not be marked as authentic.

interim		ks	
Results	◉ Authorization	◯ Corrective Approval	
Results	Approve the research protocol.		

*All researchers approved by this committee must comply with the following.
1. Prior to the approval of the protocol and any amendments to the protocol, human subjects are prohibited from participating in the study and the study must be conducted in accordance with the approved protocol.
2. Ensure that the consent process is free from coercion or undue influence, is fully informed, and that potential test subjects are given an adequate opportunity to decide whether to participate in the research.
3. Any changes to the study must be approved in advance by the committee, except for those necessary to protect human subjects. Emergency changes made to protect human subjects must also be reported to the committee immediately.
.
4. Promptly report to the committee any changes that require the study to be conducted differently from the original protocol because of the need to eliminate an immediate risk to patients, any changes that may increase the risk to patients or materially affect the conduct of the study, any unanticipated serious adverse drug reactions, or any new information that may adversely affect the safety of patients or the conduct of the study.
5. An approved informed consent form (one with the DKUHIRB stamped on it) must be used. For patients whose native language is not Korean, a certified translation of the approved consent form into the patient's native language will be used, and this translation must be approved by the committee.
6. You must use a human subjects recruitment announcement that has been approved by the committee.
7. The approval of the committee may not exceed one year, and if the researcher intends to continue the research for more than one year, he/she must submit an interim report on the progress of the research in accordance with the interim report cycle required by the committee.
8. If the outcome of the review is not approval❹, a response must be submitted within six months of the review date.
9. You can appeal if the committee rejects your research, but you can't appeal twice in a row for the same thing.
10. At the end of the study, a termination and results report must be submitted, and all studies must comply with the relevant domestic and international laws and regulations, such as the Good Clinical Practice for Pharmaceuticals and Medical Devices (CGMP), the Act on Bioethics and Safety, the Helsinki Declaration, and the ICH-GCP Guidelines.

This form was issued electronically (PDF file).
Electronic forms without a barcode cannot be verified for authenticity with the verification-only viewer and will not be marked as authentic.

Dankook University Hospital Institutional Review

This form was issued electronically (PDF file).
Electronic forms without a barcode cannot be verified for authenticity with the verification-only viewer and will not be marked as authentic.
