# Supplementary material for: The validity and safety of multispectral light emitting diode (LED) treatment on grade 2 pressure ulcer: Double-blinded, randomized controlled clinical trial
Source: PLoS One. 2024 Aug 23;19(8):e0305616. doi: 10.1371/journal.pone.0305616 (PMC11343461; doi:10.1371/journal.pone.0305616)
Supplement: S9 File — (PDF) [file pone.0305616.s017.pdf]

통지서

※ 본 과제의 문서보존기간은 3 년입니다.					
수신	의뢰(지원)기관	보건복지부			
	연구책임자	성형외과 임남규			
IRB File No.	DKUH 2020-09-014-013	심사내용	중간보고서	통지일자	2021.09.24
연구과제명	국문	경도 욕창 이환 환자를 대상으로, 의료용 광선 조사기 'BELLALUX Lite'의 창상 회복에 대한 안전성 및 유효성을 평가하기 위해, 단일기관, 이중 눈가림, 무작위배정, 평행설계(sham기기 대조)의 전향적 탐색 임상시험			
	영문				
임상시험코드		Study Nick Name			

연구분류1	☐ 약물 ☐ 생물학적 제재 ☐ 세포치료제 ☐ 건강기능식품
	☐ 의료시술 ■ 의료기기 (○ 1등급 ● 2등급 ○ 3등급 ○ 4등급)
	☐ 해당사항없음
연구분류2	■ 인간대상연구 ☐ 인체유래물(검체)연구 ☐ 의무기록연구
	☐ 유전자연구 ☐ 유전자치료
	☐ 배아연구 ☐ 체세포복제배아연구 ☐ 줄기세포주연구
	☐ 기타 ( )
연구분류3	● 전향적 연구 ○ 후향적 연구 ○ 전항적 & 후향적 병행연구
연구분류 4	☐ 중재연구 ☐ 설문조사 ☐ 자료분석 및 분석연구
	■ 관찰연구 (☐ 단면조사연구 ■ 환자대조군연구 ☐ 코호트 연구)
	☐ 기타 ( )
연구분류 5	☐ 인간을 대상으로 하지 않는 연구 Non-clinical study (in vitro. in vivo preclinical study)

일반명			상품명			
전체피험자증례수	전체	38 명	국내	38 명	본원	38 명
연구승인기간	2021.10.28 ~ 2022.10.27					

본 서식은 전자서식(PDF 파일)으로 발급되었습니다.

바코드가 입력되지 않은 전자서식은 확인용 전용뷰어로 진본 여부를 확인할 수 없으며, 진본 여부가 표시되지 않습니다.

지원의뢰기관	기관명	보건복지부	대표(직위)		성명	
제출서류목록	(첨부) [DKHUPS01_PU] AE LIST_210830 [NA] [30 / Sept / 21]<br>(첨부) [DKHUPS01_PU] 대상자리스트_210830 [NA] [30 / Sept / 21]<br>(첨부) [DKHUPS01_PU] 변경현황 요약표_210830 [NA] [30 / Sept / 21]<br>(첨부) [DKHUPS01_PU] 심사비 입금 확인서_210830 [NA] [30 / Sept / 21]<br>(첨부) [DKHUPS01_PU] 지속심사신청서_210830 [NA] [30 / Sept / 21]<br>(첨부) [DKHUPS01_PU] 동의서 서명본 Sub1~19 [NA] [30 / Sept / 21]					
관련근거	대면회의	2021.09.15				
중간보고시기	2022년 08월 27일까지	비고				
심사결과	◉ 승인 ○ 시정승인 ○ 보완 ○ 반려					
심사결과	*중간보고서<br>1. 2020.10.28. 본원대상자 38명으로 승인된 의료기기(2등급) 탐색 임상시험.<br>2. 현재까지 19명 스크리닝되어 등록 후, 6명 중도 탈락(동의철회(4명), 이상반응(2명)), 11명 연구 완료, 2명 진행 중.<br>3. SAE(중대한 이상반응) 없음.<br>4. 제출서류<br>-중간보고서<br>-대상자 리스트<br>-동의서 사본 20부. (Version 업데이트로 인해 1부 재동의 포함)<br>-변경현황 요약표<br>-AE(이상반응) 리스트					

*본 위원회에서 승인된 모든 연구자들은 다음의 사항을 준수하여야 합니다.
1. 연구계획서 및 변경계획서의 승인 이전에 연구대상자의 해당임상연구 참여가 금지되며, 승인받은 계획서에 따라 연구를 수행하야 합니다.
2. 강제 혹은 부당한 영향이 없는 상태에서 충분한 설명에 근거한 동의 과정을 수행할 것이며, 잠재적인 시험 대상자에게 연구 참여여부를 결정할 수 있도록 충분히 기회를 제공하여야 합니다.
3. 연구진행 시 시험대상자를 보호하기 위해 불가피한 경우를 제외하고 어떠한 변경이든 위원회의 사전 승인을 받고 수행하여야 합니다. 시험대상자들의 보호를 위해 취해진 응급 상황에서의 변경도 즉각 위원회에 보고하여야 합니다.
4. 시험대상자에게 발생한 즉각적 위험 요소의 제거가 필요하여 원 계획서와 다르게 연구를 실시하여야 하는 경우, 시험대상자에게 발생하는 위험요소를 증가 시키거나 연구의 실시에 중대한 영향을 미칠 수 있는 변경사항, 예상하지 못한 중대한 이상약물반응에 관한 사항, 시험대상자의 안전성이나 임상시험의 실시에 부정적인 영향을 미칠 수 있는 새로운 정보에 관한 사항은 위원회에 신속히 보고하여야 합니다.
5. 승인 받은 시험대상자 동의서（DKUHIRB가 천공 날인된 동의서）를 사용하여야 합니다. 모국어가 한국어가 아닌 시험대상자들에게는 승인된 동의서를 시험대상자의 모국어로 인증된 번역본을 사용할 것이며, 이러한 동의서 번역본은 반드시 위원회의 승인을 받아야 합니다.

본 서식은 전자서식(PDF 파일)으로 발급되었습니다.
바코드가 입력되지 않은 전자서식은 확인용 전용뷰어로 진본 여부를 확인할 수 없으며, 진본 여부가 표시되지 않습니다.

6. 위원회의 승인을 받은 시험대상자 모집공고문을 사용해야 합니다.
7. 위원회의 승인은 1년을 초과할 수 없으며 1년 이상연구를 지속하고자 하는 경우에는 반드시 중간보고를 하여야 하며, 위원회에서 요구한 중간보고 주기에 따라 연구 진행과 관련된 중간보고서를 제출하여야 합니다.
8. 심의결과가 승인이 아닐경우에는 답변서를 제출하여야 하며, 심의일로부터 6개월 이내에 이루어져야 합니다.
9. 위원회가 연구를 반려하는 경우 이의신청을 할 수 있으며, 같은 사항에 대하여 2번 연속으로 이의 신청은 할 수 없습니다.
10. 연구 종료 연구 종료시에는 종료 및 결과보고서를 제출해야 하며 모든 연구의 진행은 의약품임의약품임상시험관리기준 / 의료기기임상시험실시기준（KGCP）, 생명윤리 및 안전에 관한 법률 및 헬싱키 선언, ICH-GCP가이드라인 등 국내외 관련법규를 준수하여야 합니다.

단국대학교병원 Institutional Review Board

본 서식은 전자서식(PDF 파일)으로 발급되었습니다.

바코드가 입력되지 않은 전자서식은 확인용 전용뷰어로 진본 여부를 확인할 수 없으며, 진본 여부가 표시되지 않습니다.
