# Supplementary material for: The validity and safety of multispectral light emitting diode (LED) treatment on grade 2 pressure ulcer: Double-blinded, randomized controlled clinical trial
Source: PLoS One. 2024 Aug 23;19(8):e0305616. doi: 10.1371/journal.pone.0305616 (PMC11343461; doi:10.1371/journal.pone.0305616)
Supplement: S12 File — (PDF) [file pone.0305616.s020.pdf]

Subscribe to DeepL Pro to translate larger documents.
Visit www.DeepL.com/pro for more information.

Notices

The document retention period for this assignment is 3 years.						
Receives	Referral (support) organizations	Department of Health and Human Services				
	Principal Investigator	Namkyu Lim, Plastic Surgery				
IRB File No.		DKUH 2020-09-014-019	Review Content	Exit Report	Notification Date	October 28, 2022
Project name	Korean	A single-center, double-blind, randomized, parallel-group, prospective exploratory clinical trial to evaluate the safety and efficacy of the medical light irradiator BELLALUX Lite on wound healing in patients with mild pressure ulcer co-morbidity.				
	English					
Study Code			Study Nick Name			

Research Category1	☐ Medications ☐ Biologics ☐ Cellular Therapeutics ☐ Nutraceuticals
	☐ Medical Procedures ■ Medical Devices (◯ Class 1 ◉ Tier 2 ◯ Tier 3 ◯ Level 4)
	☐ Not applicable
Research Category2	■ Human Subjects Research ☐ Human remains (specimen) research ☐ Medical Records Research
	☐ Genetic Resources ☐ Gene Therapy
	☐ Embryology ☐ Somatic cell cloning embryo research ☐ Stem Cell Line Research

---

This form was issued electronically (PDF file).
Electronic forms without a barcode cannot be verified for authenticity with the verification-only viewer and will not be marked as authentic.

	☐ Other ( )					
Research Category3	◉ Prospective studies	○ Retrospective studies	○ Prospective & Retrospective Parallel Studies			
Research Category 4	☐ Interventional Studies	☐ Surveys	☐ Data analysis and research			
	■ Observational studies (	☐ cross-sectional study	■ Patient-controlled studies ☐ Cohort Studies)			
	☐ Other ( )					
Research Category 5	☐ Studies not involving human subjects Non-clinical study (in vitro. in vivo preclinical study)					
Common name		Product name				
Total Subject Cases	All	38 people	Domestic	38 people	Headquarters	38 people
Study approval period	October 28, 2021 - October 27, 2022					
Referral sources	Organization Name	Department of Health and Human Services	Representative (position)		First Name	
List of Submission Documents	(Attachment) [DKHUPS01_PU] Destination list (end) [] []<br>(Attachment) [DKHUPS01_PU] Termination Report [] []<br>(Attachment) [DKHUPS01_PU] Clinical Study Checklist (Exit) [] [] (Attachment) [DKHUPS01_PU] Signed Consent Form [] []					
Related Rationale	Evaluation Date	October 26, 2022				
When to report interim		Remarks				
Results	◉ Authoriz	○ Corrective Approval				

This form was issued electronically (PDF file).
Electronic forms without a barcode cannot be verified for authenticity with the verification-only viewer and will not be marked as authentic.

	ation
Results	*Exit Report<br>1. About research projects<br>-2020.10.28. Exploratory medical device (class 2) trial approved with 38 primary subjects.<br>2. Enrollment Status<br>-Home institution assignments: 38<br>-Screening: 40 people<br>-Failed screening: 2 people<br>-Registrations: 38<br>-Dropouts: 11<br>-Study completed: 27 people<br>3. Submissions<br>-Exit Report<br>-Audiences list<br>-Clinical research checklist<br>-a copy of the consent form

*All researchers approved by this committee must comply with the following.

1. Prior to approval of the protocol and amendment, the subject is prohibited from participating in the study.
2. Conducting a study in accordance with the approved protocol is prohibited. Conducting a study that differs from the original protocol prior to approval of the amended protocol is prohibited.
3. Ensure that the consent process is free from coercion or undue influence, is fully informed, and that potential subjects are given an adequate opportunity to decide whether to participate in the research.
4. Any changes to the study must be approved in advance by the committee, except for those necessary to protect human subjects. Emergency changes made to protect human subjects must also be reported to the committee immediately.
5. circumstances that require the study to be conducted differently from the original protocol due to the need to eliminate an immediate risk factor to subjects❹; changes that may increase the risk to subjects or have a significant impact on the conduct of the study; unanticipated adverse drug reactions; new information that may adversely affect the safety of subjects or the conduct of the study.

This form was issued electronically (PDF file).
Electronic forms without a barcode cannot be verified for authenticity with the verification-only viewer and will not be marked as authentic.

Any information regarding fortunes shall be reported promptly to the Committee.

6. An approved human subjects consent form (consent form stamped by DKUH IRB/EC) must be used. For subjects whose native language is not Korean, a certified translation of the approved consent form into the subjects' native language will be used, and the translation of the consent form must be approved by the committee.
7. You must use a human subjects protocol that has been approved by your committee.
8. If you wish to continue your research for more than one year, you must apply for a continuation review and submit a continuation review application related to your research progress in accordance with the continuation review cycle required by the committee.

9. If the outcome of the review is not approval❹, a response must be submitted within six months of the review date.
10. You can appeal any of the committee's findings, but you can't appeal the same issue twice in a row.
11. At the end of the study, you will need to submit an exit and results report.
12. All studies must comply with relevant domestic and international laws and regulations, including the Good Clinical Practice for Pharmaceuticals / Good Clinical Practice for Medical Devices (KGCP), the Act on Bioethics and Safety, the Helsinki Declaration, and the ICH-GCP Guidelines.
13. Approved research may be subject to internal and external inspections. Researchers are expected to cooperate with internal inspectors, external monitors and inspectors, and regulatory agency inspectors when they request access to research-related documents (including electronic documents).
14. Reports on the progress of the study shall be submitted to the Committee when requested by the Committee.

Dankook University Hospital Institutional Review [illegible]

This form was issued electronically (PDF file).
Electronic forms without a barcode cannot be verified for authenticity with the verification-only viewer and will not be marked as authentic.
