# Supplementary material for: The validity and safety of multispectral light emitting diode (LED) treatment on grade 2 pressure ulcer: Double-blinded, randomized controlled clinical trial
Source: PLoS One. 2024 Aug 23;19(8):e0305616. doi: 10.1371/journal.pone.0305616 (PMC11343461; doi:10.1371/journal.pone.0305616)
Supplement: S13 File — (PDF) [file pone.0305616.s021.pdf]

# 연구 계획서(인간 대상 연구용)

ver 2.0

| 연구제목 |
|---|
| 경도 욕창 이환 환자를 대상으로, 의료용 광선 조사기 ‘BELLALUX Lite’의 창상 회복에 대한 안전성 및 유효성을 평가하기 위해, 단일기관, 이중 눈가림, 무작위배정, 평행설계(sham기기 대조)의 전향적 탐색 임상시험 |

단국대학교병원 성형외과

# 1. 연구배경

욕창은 전단력과 마찰력을 포함한 복합적인 압력에 의해 피부 및 피하지방이 국소적 손상을 받는 것을 의미하며, 주로 뼈의 돌출부위나 의료 기기 등의 물체에 의해 발생함. 연구에 따라 이환율은 0%~75%까지 보고되고 있으며, 평균적으로 6.3%가량 발생하는 것으로 알려져 있음.[1] 특히 중환자실 환자(8.8%~12.1%)나 척수 손상 환자(33%~60%)의 경우 욕창 이환 가능성이 높음.[2]

국내의 통계는 없지만 미국 통계의 경우, **욕창 환자는 연 250만명**으로 파악되며 이로 인한 **사망도 연간 6만명**으로 보고됨. 이는 연간 독감으로 사망하는 환자(5.6만명)와 자살로 인하여 사망하는 환자(4.4만명)보다 상회하는 수치임. 이렇게 사회적인 문제로 대두되는 욕창은 **연간 약 116억 달러의 사회적 비용을 소요**함. 인당 소요 비용 또한 500달러에서 15만달러에 이름.[1-3]

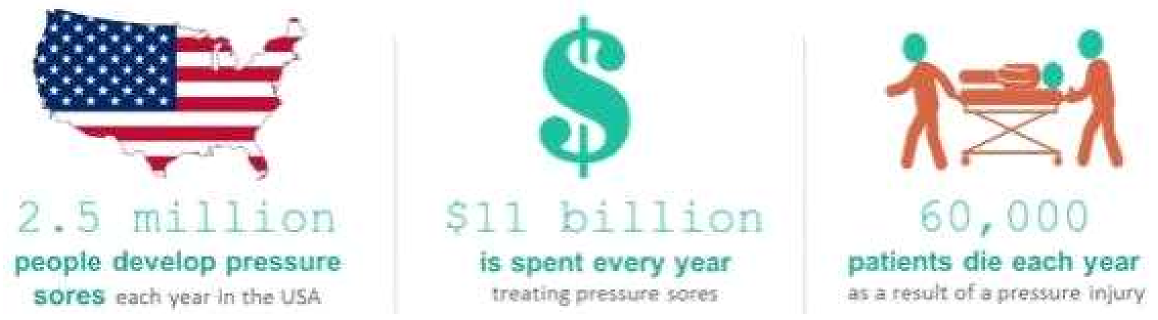


**그림 1.** 욕창 환자의 이환 정도와 연간 사회적 비용 (미국 통계)

일반적으로 모세혈관의 평균 압력은 32mmHg 정도이며, 이의 두 배의 압력(70~80mmHg)이 2시간 이상 지속될 경우 허혈성 괴사가 진행됨. 특히 전단력과 마찰력이 동반될 경우 배의 압력으로 조직에 작용되어 괴사의 진행이 빨라짐. 병리 기전 상 욕창은 우리 몸 전체에 발생 가능한 질환이며, 연구에 따라 다르지만 일반적으로 엉치(28.3%), 뒤꿈치(23.6%), 좌골(17.2%) 순으로 흔히 발생함.[2]

---

1) NPIAP-EPUAP-PPPIA. Prevention and Treatment of Pressure Ulcers/Injuries: Clinical Practice Guideline. The International Guideline 2019

2) Robert K, Juan LR, Jeffery E. Pressure Sores. Neligan 4th edition. Elsevier. 2016 Vol. 4;350-380.

3) William VP, Benjo AD. The National Cost of Hospital-Acquired Pressure Injuries in the US. Int Wound J. 2019;16(3):634-640.

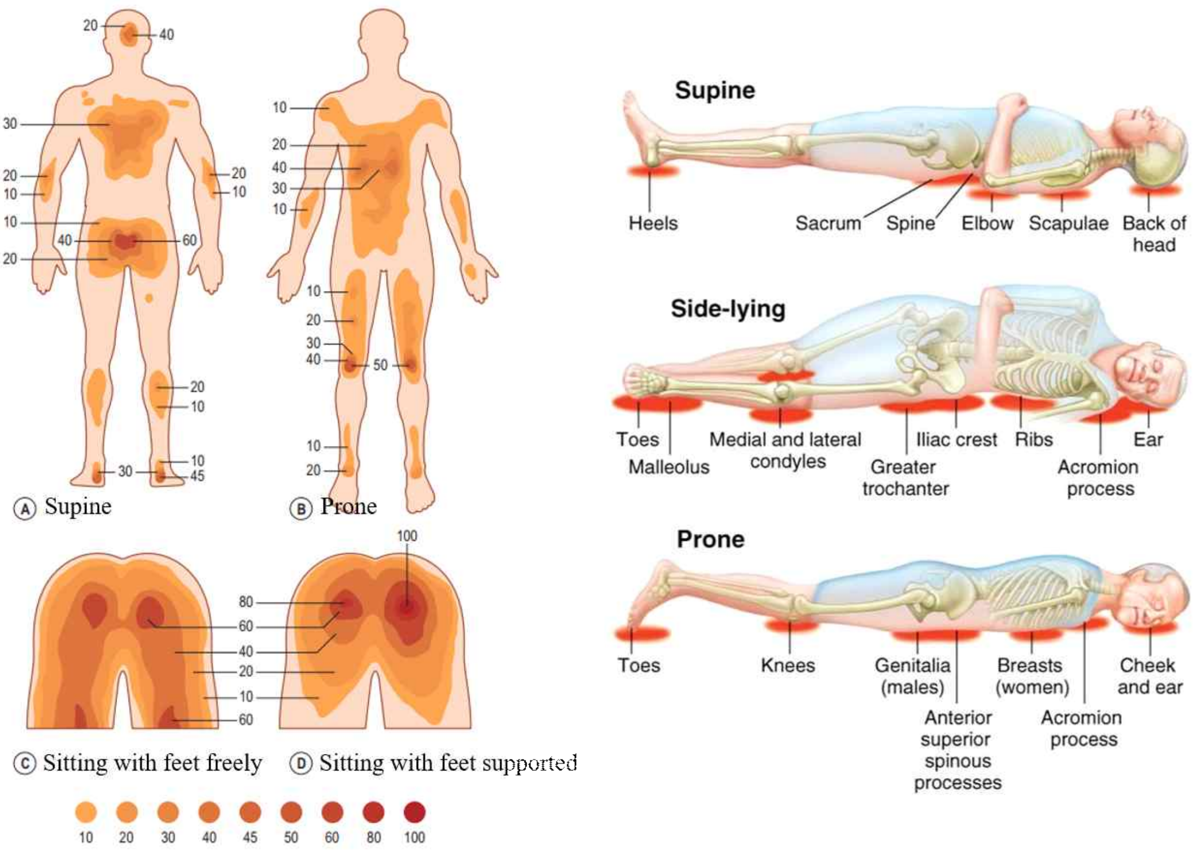


**그림 2.** 자세에 따른 연부조직 부하 압력과 욕창 호발 부위

발생된 욕창은 다양한 방법으로 분류할 수 있으나, 최근 가장 많이 이용되고 있는 방법은 National Pressure Ulcer Advisory Panel (NPUAP)에서 공표한 단계 분류법임. 이는 깊이에 따라 1~4단계로 나누며, 2가지의 미분류 욕창을 포함하여 총 6단계로 분류한 방법임.[1] **NPUAP 2단계 부터는 피부 손상**을 보이며, 3단계 이상이 되면 피부 조직 괴사가 발생하여 변연절제술이 필요함.

욕창은 염증 반응이 지속적으로 발생하는 만성 창상의 전형적인 예시이며, 결손된 조직은 범위와 깊이에 따라 **회복 기간**에 차이가 있지만 **경도의 경우 1~3개월, 중등도 이상은 최소 6개월~1년 이상의 시간**을 필요로 함. 하지만 이의 회복은 반드시 달성되는 것은 아니며, 직접 원인이 되는 압박의 완화와 더불어 지속 압박에 이르기까지 해결되지 못했던 간접 원인인 내과적 질환 관리 및 영양, 지속적인 창상관리 등이 원활하게 수행될 경우 달성 가능함.

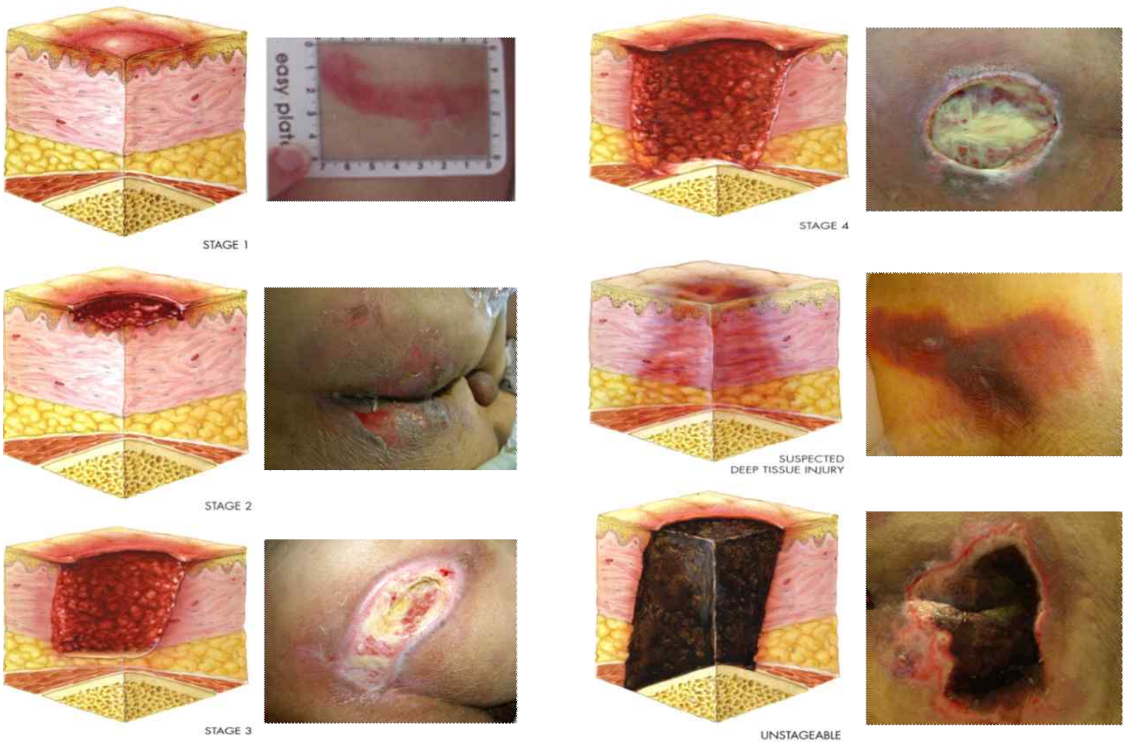


그림 3. NPUAP 욕창 분류법

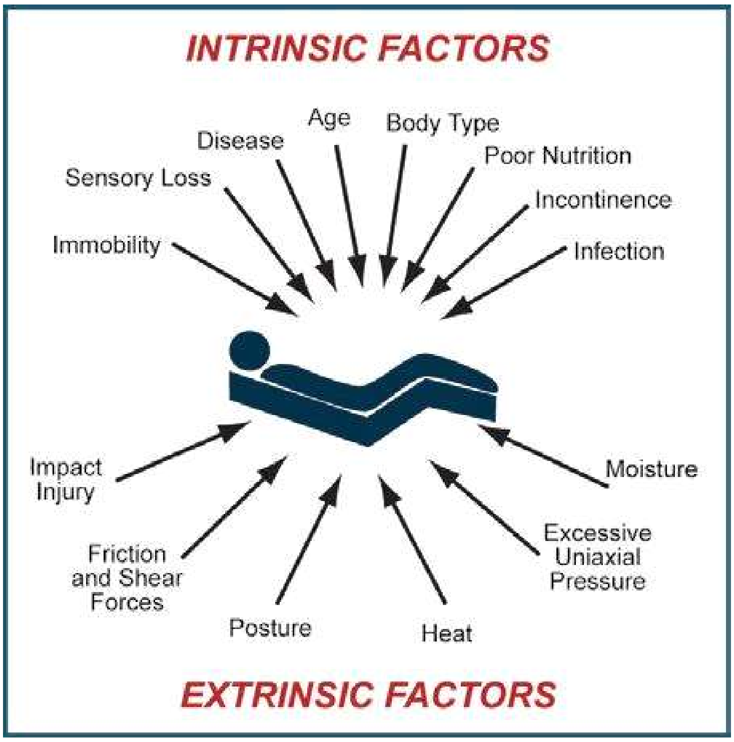


그림 4. 욕창의 다인적 요인

이렇듯 **다인적 요인의 큰 사회적 비용이 소요되는 욕창은 세계적인 이슈**이며, 미국과 유럽, 환태평양의 의료진들이(National Pressure Injury Advisory Panel - European Pressure Ulcer Advisory Panel - Pan Pacific Pressure Injury Alliance) 2009년부터 지금까지 3차례 '욕창 국제 가이드라인'을 발표하였음(2009 초판, 2014, 2019 개정). 해당 가이드라인에 따르면 **욕창의 가장 좋은 치료 방법은 예방**이며, **체위 변경의 중요성을 강조**하고 있음. 연구에 따라 다르지만, 최대 95%까지 욕창은 예방 가능하다고 알려져 있음.[1]

하지만 한번 발생된 피부 괴사는 감염을 동반할 수 있기에 변연절제술이 선행되어야 함. 변연절제 후 결손 부위는 피판술 등을 통한 수술적 치료로 복원할 수도 있지만, 전신마취를 수행할 수 없을 경우에는 **보존적 치료를 시행**할 수밖에 없음. 이 경우 **염증 조절과 창상 회복 과정의 촉진을 유도하는 것이 매우 중요**함. 오늘날까지 많은 창상 피복재가 개발되었으며, 환부 상태에 적합한 소독재제를 선택함으로써 창상 회복을 유도하고 있음. 하지만 치료제는 각각의 한계가 있기에 욕창의 보존적 치료에는 정해진 방법이 없음.

환부에 저출력 광선을 조사하여 치유를 유도하는 **광생물 조절 치료 (photobiomodulation therapy)** 또한 산화조절 및 성장 인자를 촉진시킴으로써 창상 치유에 도움된다는 보고가 많이 있음. 그 외에도 항염증효과, 진통, 살균 등에도 도움된다고 알려져 있으며, 욕창에 대해서도 광생물 조절 치료를 적용하여 그 효과를 검증한 논문도 보고됨.[4,5] 하지만 아직까지 광생물 조절 치료의 정확한 기전에 대해서는 명확하게 밝혀지지 않음.

Francislene FCP 등이 2020년에 보고한 5개 논문에 대한 체계적 리뷰에 의하면, **658 nm 파장의 경우 욕창 치유에 의미있는 결과를 도출**하였지만, 808 nm 혹은 990 nm의 경우는 유의한 차이를 보이지 않았음. 대부분 연구에서 Energy density는 1 J/cm$^2$ or 4 J/cm$^2$을 사용하였고, 주 3~5회 4~6 주간 치료를 시행하였음. 치료 효과의 분석은 환부 크기 및 깊이, 재상피화 정도, 성장인자 분비 및 사이토카인의 정량 분석 등을 통해 시행하였음. 하지만 해당 연구들에 대해 선입견 지표(bias)를 분석하였을 때, 완성도 높은 연구는 거의 없었음. 또한 최종 결과에 대한 분석만 있을 뿐, 만성 창상의 회복 과정에 대한 분석은 없음.[5]

4) Chen C, How WH, Chan ESY, Yeh ML, Lo HLD. Phototherapy for Treating Pressure Ulcers. Cochrane Database Syst Rev. 2014;11(7):CD009224.

5) Francislene FCP, Jorge VCF, Hellen R, et al., Effect of Photobiomodulation on Reparing Pressure Ulcers in Adult and Elderly Patients: A systematic Review. Photochem Photobiol. 2020;96(1):191-199.

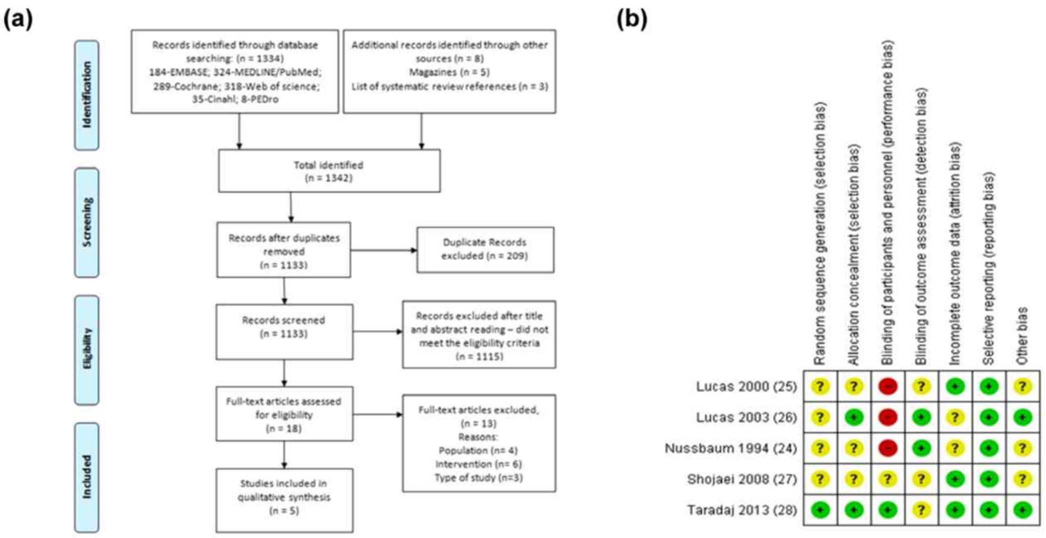


**그림 5.** (a) 체계적 분석위한 연구 선택 흐름도. (b) 선택된 5개 연구의 선입견 (bias) 조사표

본 임상시험에 이용할 **'BELLALUX Lite'**는 2019년 7월 IECEE-CB(IEC System for Conformity Testing and Certification of Electrical Equipment-Certified Body) 인증을 받은 제품으로 **2등급 의료용 조합 자극기**임. 이 제품은 전신인 'BELLALUX(모델명 : RED&AMBER-MD1)'의 성능 개선 및 경량화한 제품으로, RED (630 nm) 및 AMBER (595 nm) 파장 외에도 BLUE (460 nm) 및 NIR (850 nm) 4개의 파장을 냄.

전신 모델인 'BELLALUX'의 욕창치료 효능검증을 위해 **Rat을 이용한 전임상 시험 2019년도 본원에서 수행**하였음. 이 결과에 의하면 국소 림프절의 피부 감작성 평가에서 대조군에 비해 자극 지표(SI : Stimulation Index)가 0.7 ~ 1.3 수준으로, 피부 감작 물질로 판단하는 SI 1.6에 비해 낮아 **안전성이 증명**되었음.

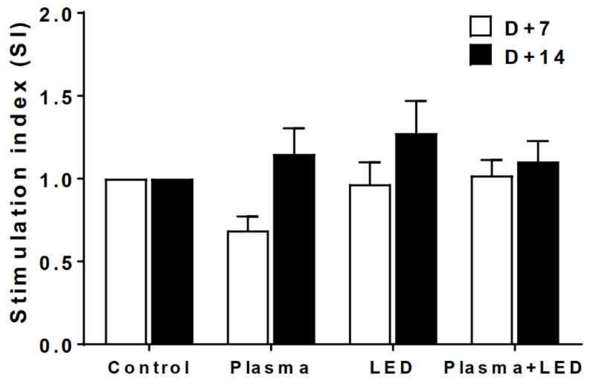


**그림 6.** 안전성 평가에 대한 전임상 시험 결과 (단국대학교 의과대학 광생물학실험실)

유효성 평가에 대해서는 감염성 창상에 대한 회복력을 기준으로 삼았으며, 대조군에 비해 플라즈마와 광선을 각각 조사한 군에서 창상 크기가 유의한 차이로 줄어드는 것을 확인할 수 있었고, 특히 플라즈마와 광선을 동시에 조사한 실험군에서 더 빠른 회복을 보였음.

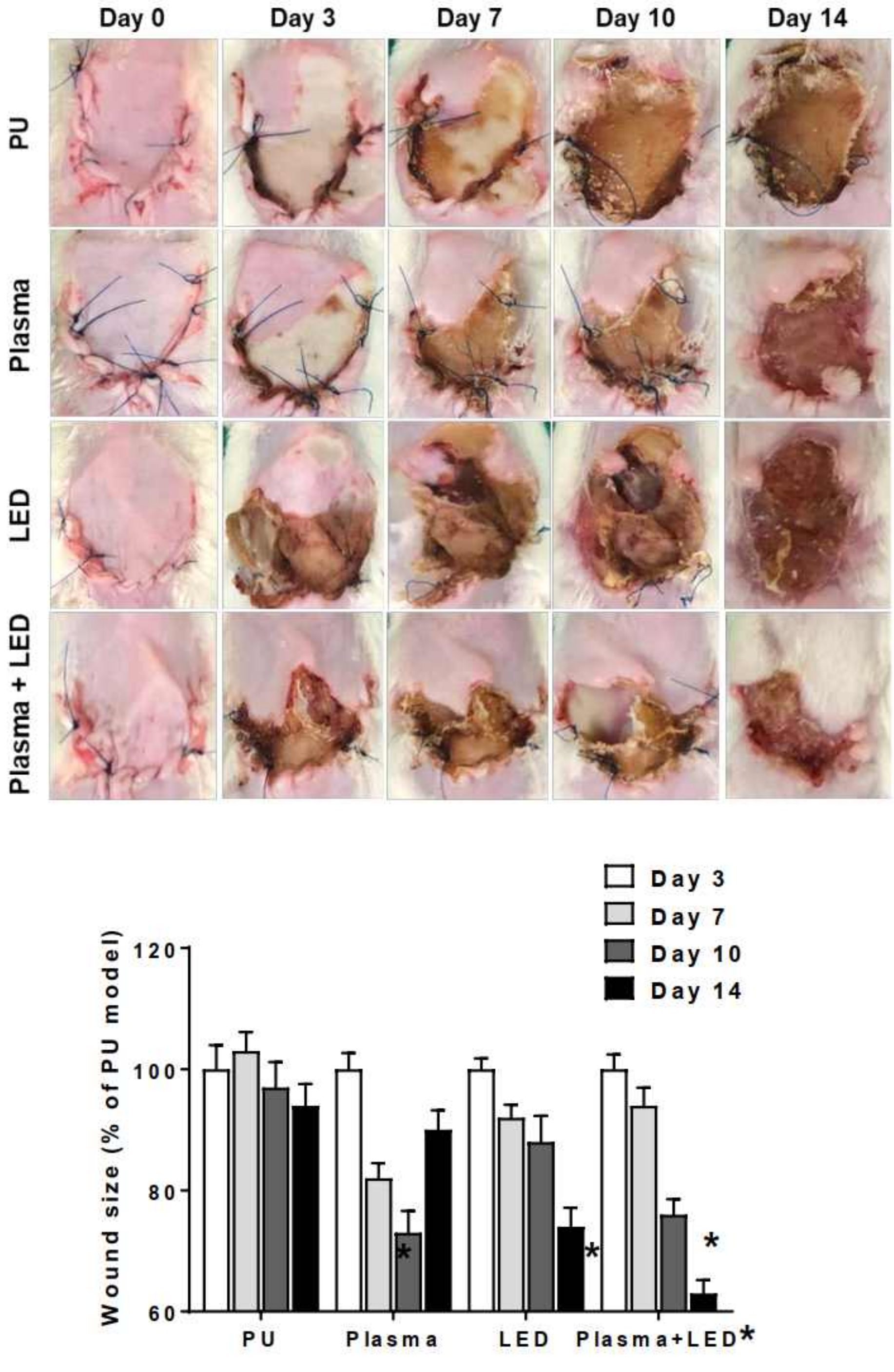


그림 7. 유효성 평가에 대한 전임상 시험 결과 (단국대학교 의과대학 광생물학실험실)

이에 **임상적으로도 해당 의료기기의 유효성과 안전성을 평가의 필요성이 있어 본 임상시험을 계획**함. 전임상 시험에서 이용된 플라즈마의 경우 발생되는 오존에 의해 살균력이 발생하여 상기 실험과 같은 감염성 창상에 효과적이지만, 사람에 있어 해당 오존의 발생이 잠재적 위험을 가질 수 있기에 임상시험에는 적합하지 않음. **전임상 시험에서 사용한 모델의 후속 모델인 본 임상시험 기기(BELLALUX Lite)의 경우에는 460 nm의 BLUE 파장도 추가되어 향상된 살균력을 기대할 수 있음**. BLUE 파장의 살균력에 대해서는 본 연구실에서 다음과 같은 선행연구를 수행한 바 있음.

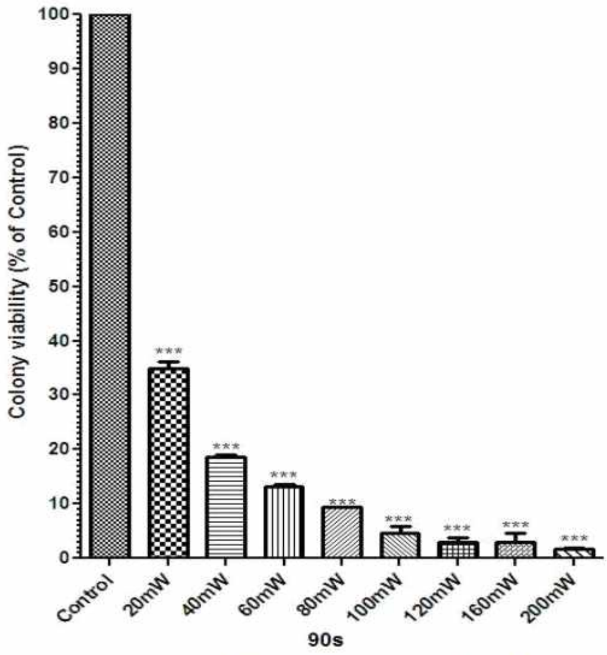


Fig. 6 90s, 20mW, 40mW, 60mW, 80mW, 100mW, 120mW, 160mW, 200mW로 조사한 *Propionibacterium acnes* 균의 viability

**그림 8.** 408 nm 파장에 대한 혐기성 균인 *Propionibacterium acnes* 의 생존율

**따라서, 본 임상기기는 만성 창상의 전형인 욕창에 대해 충분한 살균력(BLUE 파장)과 창상 회복력(RED+AMBER 파장)을 기대할 수 있겠으며, 본 기기로의 임상시험의 시도는 충분히 그 의의가 있겠음.**

또한, 본 임상시험은 대조군에서도 통상적인 보존적 치료(항균 소독)을 수행하면서 실험군에서 추가적으로 LED 치료기기의 효과를 보는 것이라 최소 위험을 벗어나지 않음.

○ **임상시험용 의료기기의 개요**

● **제품개요**

• **개발 경위**

㈜링크옵틱스에서 개발한 저출력광선조사기와 적외선조사기가 조합된 2등급의료용조합자극기품목으로 전임상시험을 통해 욕창에도 치료에도 효과가 있음을 확인하여 제품을 개발함.

• **작용 원리**

본 제품은 저출력광선조사기와 적외선조사기가 조합되어 사용되는 2등급의료용조합자극기로, 전기에너지가 인가되면 가시광선[RED (630nm), AMBER (595nm), BLUE (460nm)] 파장을 가진 가시광선 LED 램프와 근적외선 (NIR, 850nm) 파장의 근적외선 램프를 통해 광(光)의 형태로 에너지를 방출하게 되고, 이 빛이 세포내에 흡수되어 세포 기능이 활성됨으로 피부 질환 치료, 욕창 치료 등에 사용하기 위해 개발한 기기임. 본 제품은 조사부와 본체부로 크게 구성된다. 조사부는 램프가 부착되고 전원 공급 시 가시광선 및 적외선을 방사하고 조사부 연결부를 통해 조사부의 각도와 방향을 조절하는 기능을 하며, 본체부는 조사부를 지지하고 제품의 높이 조절을 가능하게 함.

● **모양 및 구조 (외관)**

• 외관형태

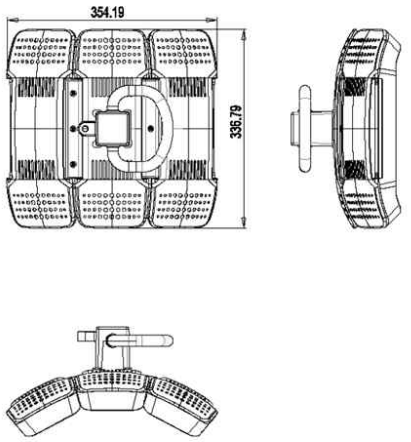


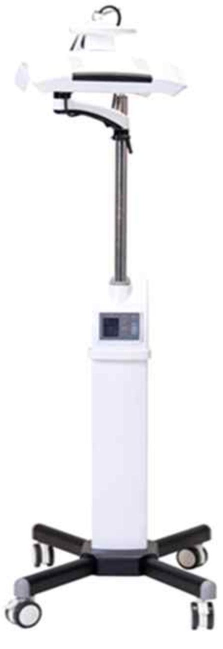

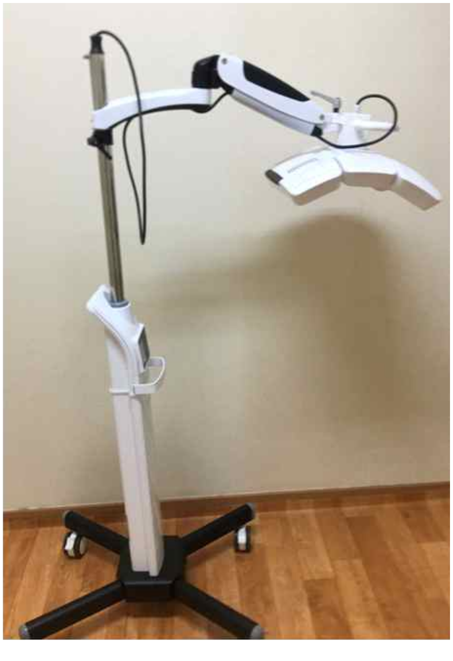

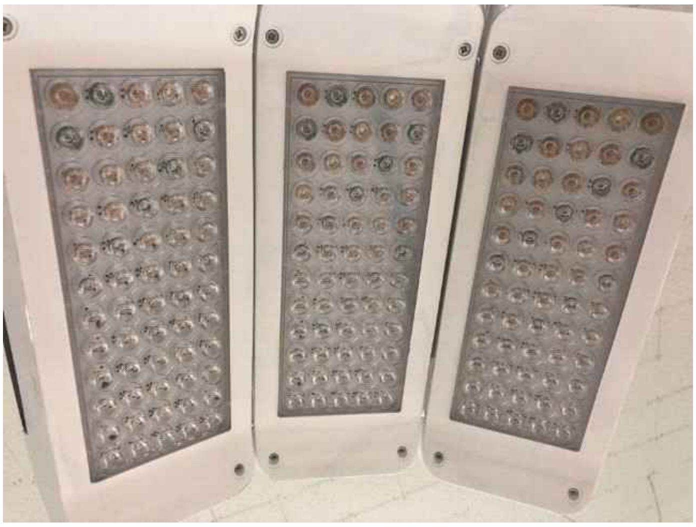

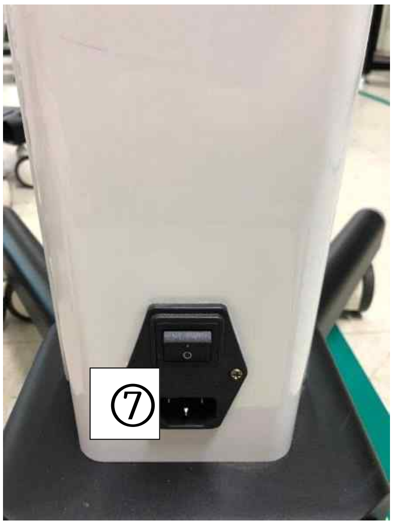


그림 9. ‘BELLALUX Lite’ 및 제품 외관

- **외관설명**

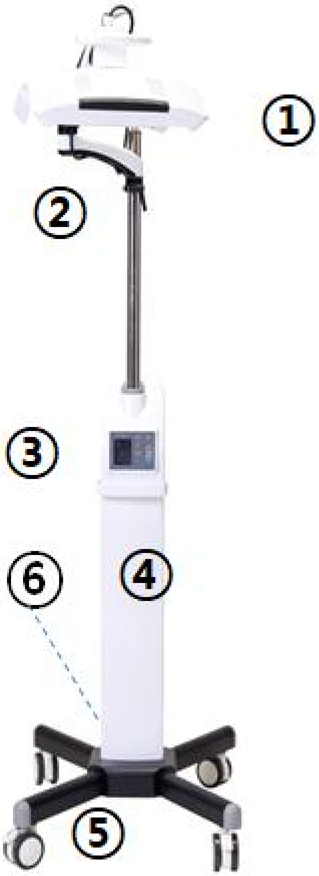


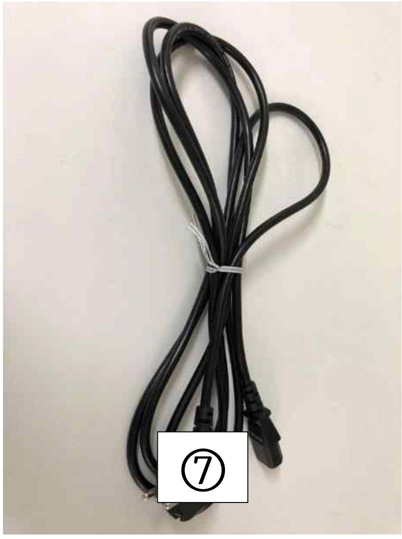

<table>
<tr><th>번호</th><th>명 칭</th><th>기 능</th></tr>
<tr><td>①</td><td>조사부</td><td>광원부 - LED 광선 출력 [15cm 위치에서의 출력단위(mW/㎠)]
<table>
<tr><th>파장 / LED 갯수</th><th>1단계</th><th>2단계</th><th>3단계</th><th>4단계</th><th>5단계</th></tr>
<tr><td>Blue(460nm) /45개</td><td>0.80</td><td>2.20</td><td>3.60</td><td>5.10</td><td>8.40</td></tr>
<tr><td>Amber(595nm) /45개</td><td>2.00</td><td>4.50</td><td>7.00</td><td>9.00</td><td>12</td></tr>
<tr><td>RED(630nm) / 45개</td><td>3.60</td><td>8.80</td><td>13.80</td><td>19.00</td><td>30.00</td></tr>
<tr><td>NIR(850nm) / 45개</td><td>5.60</td><td>12.40</td><td>21.00</td><td>28.00</td><td>40.00</td></tr>
</table></td></tr>
<tr><td>②</td><td>조절부</td><td>광원부의 높이 및 방향 조정</td></tr>
<tr><td>③</td><td>LCD화면 & 버튼</td><td>LCD 화면과 작동 버튼</td></tr>
<tr><td>④</td><td>본체</td><td>제품의 본체</td></tr>
<tr><td>⑤</td><td>이동형 받침대</td><td>본체를 지지해주며 기기를 이동</td></tr>
<tr><td>⑥</td><td>전원부</td><td>제품의 전원</td></tr>
<tr><td>⑦</td><td>전원 코드</td><td>제품 전원 코드</td></tr>
</table>

- **버튼 설명**

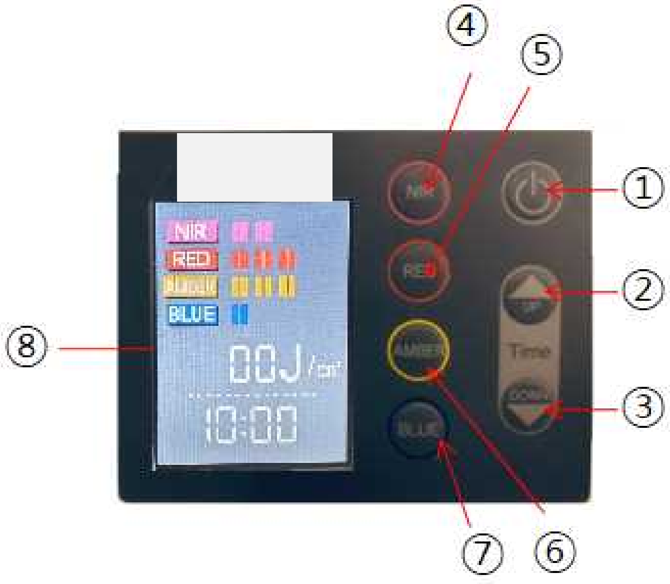


- 외부 작동 버튼 설명

| 번호 | 명 칭 | 기 능 |
|---|---|---|
| ① | 작동 버튼 | 기기 작동 유무 스위치 |
| ② | 시간 조절 올림 버튼 | 최대 25분까지 5분단위로 올릴 수 있음 |
| ③ | 시간 조절 내림 버튼 | 최소 5분까지 5분단위로 내릴 수 있음 |

| | | |
|---|---|---|
| ④ | NIR | NIR파장을 조절 할 수 있는 스위치<br>0~5단계로 설정 가능 |
| ⑤ | RED | RED 파장을 조절할 수 있는 스위치<br>0~5단계로 설정 가능 |
| ⑥ | AMBER | AMBER파장을 조절할 수 있는 스위치<br>0~5단계로 설정 가능 |
| ⑦ | BLUE | BLUE파장을 조절할 수 있는 스위치<br>0~5단계로 설정 가능 |
| ⑧ | LCD화면 | LCD화면 현재의 상황을 표시 |

- **모양 및 구조 (특성)**
- **작동원리**

본 제품은 LED(Light Emitting Diode)를 이용하여 만든 램프에 제품 전원을 통해 전기적 에너지를 가하면 LED(Light Emitting Diode)에서 나오는 가시광선과 적외선의 빛 에너지를 인체의 피부질환 및 욕창의 환부에 조사하여 사용함.

- **전기적 정격**

- 정격전압 : 220V AC
- 정격주파수 : 60Hz
- 소비전력 : 200W

- **전기 충격에 대한 보호형식 및 보호정도에 의한 분류**

- 1급 기기, 장착부 없음

- **안전장치**

- Fuse(2A) : 전원이 단락(short circuit)되거나, 장비에 이상이 있을 시 자동적으로 장비의 전원을 차단시켜 줌.

- **내장 소프트웨어**

- 모델명 : Bellalux Lite FW
- 명칭 : Lite 광원 제어 프로그램
- 소프트웨어 안전성 등급 : A

- 의료기기 사이버 보안 안전성 등급 : 중

- 알고리즘, 구조, 주요기능

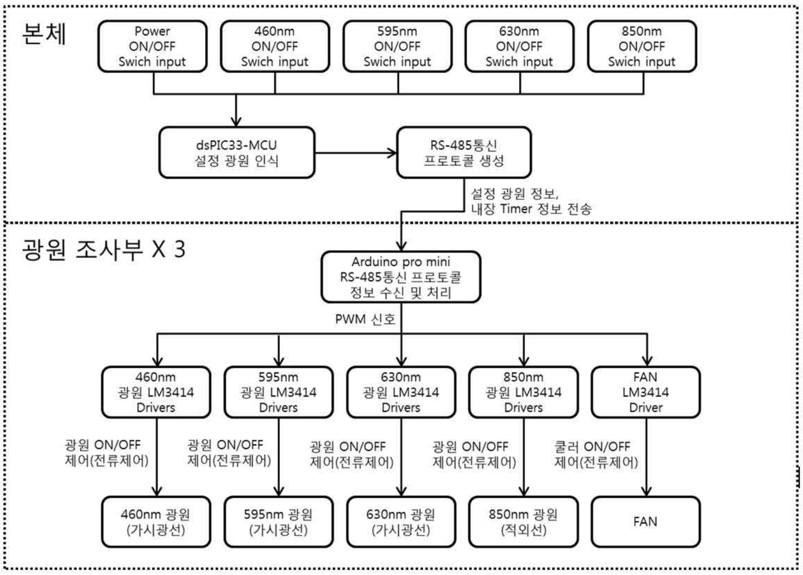


**그림 10.** ‘BELLALUX Lite’ 알고리즘 모식도

● **성능 : 광선출력 (조사부에서 15cm 떨어진 곳)** [출력단위 (mW/cm$^2$)]

| 파장 | 1단계 | 2단계 | 3단계 | 4단계 | 5단계 |
|---|---|---|---|---|---|
| Blue | 0.80 | 2.20 | 3.60 | 5.10 | 8.40 |
| Amber | 2.00 | 4.50 | 7.00 | 9.00 | 12 |
| RED | 3.60 | 8.80 | 13.80 | 19.00 | 30.00 |
| NIR | 5.60 | 12.40 | 21.00 | 28.00 | 40.00 |

* 각 기준의 ±20%이내여야 한다.

- **출력시간** : 최대 25분이며 5분 간격으로 조절

- **광선파장** : BLUE(460nm±10nm), AMBER(595nm±10nm), RED(630nm±10nm), NIR(850nm±10nm)

- **조사면적** : LED 광조사부에서 15cm 떨어진 지점

- **안전장치** : 과전류 시 퓨즈가 단선되어 전원을 차단

● **대조기기 (Sham 기기)**

대조기기는 임상시험용 제품 BELLALUX Lite와 동일한 형상을 갖으며, 광출력은 10% 미만(대조기기의 광선 조사부는 12mW/cm$^2$ 이하의 LED)임. 시험기기에서 조사되는 조사부와 유사하게 보이도록 링크옵틱스(주)에서 제작됨. 또한 기타 외관 및 조작방법은 임상시험기기와 동일하게 제작될 예정.

## 2. 연구목적

본 임상시험은 **의료용 광선 조사기** ‘BELLALUX Lite’의 창상 회복에 대한 안전성 및 유효성을 평가하기 위해, **경도 욕창 이환 환자**를 대상으로 **단일기관, 이중눈가림, 무작위배정, 평행설계(sham기기 대조)의 전향적 탐색 임상시험**임.

- **1차 목적** : 본 임상시험의 1차 목적은 경도 욕창 치료에 대하여 임상시험용 의료기기 적용 4주 후 창상 크기 및 재상피화 정도, 면역화학적 지표를 비교하여 ‘BELLALUX Lite’의 **유효성을 평가**함.

- **2차 목적** : 임상시험용 의료기기 적용 전 대비 적용 4주 후 시험자에 의해 평가된 이학적 검사 및 이상반응 평가를 통한 ‘BELLALUX Lite’의 **안전성 평가**함.

## 3. 연구 실시 기관명 및 주소

## 4. 연구 지원기관

본 임상시험은 2020년도 제1차 범부처전주기의료기기연구개발사업(제품개발 단계별 임상지원)에 선정됨.

## 5. 연구책임자, 공동연구자, 담당자의 성명과 직명

### (1) 임상시험의 책임자 • 담당자 및 공동연구자의 성명 및 직명

### (2) 임상시험용 의료기기를 관리하는 관리자의 성명 및 직명

## 6. 연구기간

본 임상시험의 기간은 **임상시험심사위원회(Institutional Review Board : IRB) 계획 승인일로부터 30개월**로 함. 예상되는 단계별 소요기간은 다음과 같음.

**(1) 보고서 IRB 통과** : 2개월

**(2) 피험자 모집기간** : 13개월

피험자 선정기준에 속하는 엉치부위 욕창 2기 환자는 현재 본원에서 월 평균 10명의 신환이 발생함. 이 중 제외기준에 포함되는 자는 월 평균 5명임. 따라서 실험에 동의하지 않는 이를 제외한다면 **월 평균 3명의 연구 참여자가 발생할 것으로 기대함**. 이에 39명의 피험자를 모집하기 위해서는 13개월의 시간이 소요될 것으로 예측됨.

**(3) 피험자 임상기간** : 12개월

욕창은 만성 창상으로 보존적 치료를 수행했을 경우 완전 회복까지 오랜 시간이 소요됨. **경도의 경우 1~3개월**, **중등도 이상은 최소 6개월~1년 이상의 시간을 필요**로 함. 또한 **회복 후 6개월까지 창상 성숙기가 존재하기에 창상 완쾌 후에도 6개월 뒤 추적 관찰이 필요**함.

그러나 창상의 회복은 반드시 달성되는 것은 아니며, 직접 원인이 되는 압박의 완화와 더불어 지속 압박에 이르기까지 해결되지 못했던 간접 원인인 내과적 질환 관리 및 영양, 지속적인 창상관리 등이 원활하게 수행될 경우 달성 가능함. 이에 **충분한 임상기간이 필요할 것으로 사료**됨.

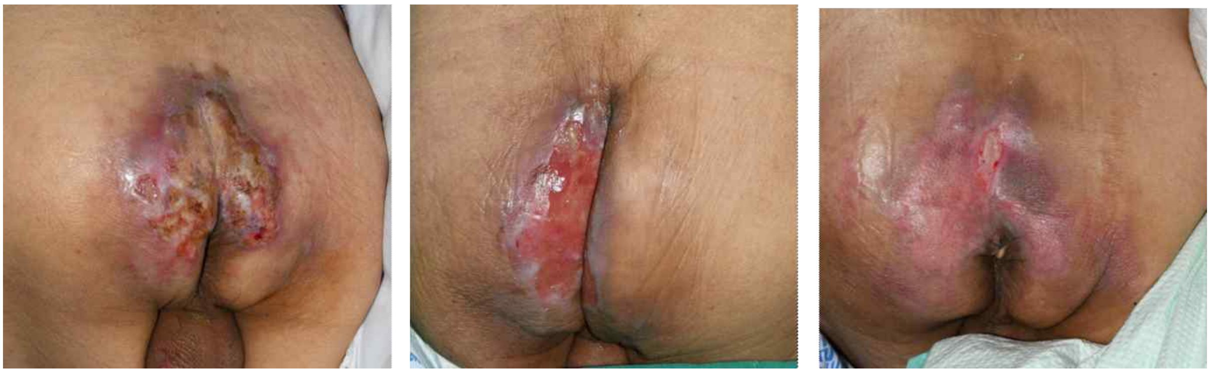

**그림 9.** 보존적 치료로 3개월에 걸쳐 회복된 엉치 부위 욕창 2기 환자

**(4) 임상결과 분석 (통계처리 기간) 및 보고서 작성** : 3개월 + 9개월(가변적)

단, 임상시험 진행에 영향을 미칠 수 있는 상황이 발생한 경우에는 기간의 변동이

있을 수 있음.

| | 2020 | | 2021 | | | | 2022 | | | | 2023 | | |
|---|---|---|---|---|---|---|---|---|---|---|---|---|---|
| | 3/4 | 4/4 | 1/4 | 2/4 | 3/4 | 4/4 | 1/4 | 2/4 | 3/4 | 4/4 | 1/4 | 2/4 | 3/4 |
| IRB 승인 | | | | | | | | | | | | | |
| 피험자 모집 | | | | | | | | | | | | | |
| 임상시험 | | | | | | | | | | | | | |
| 결과분석 및 보고서 작성 | | | | | | | | | | | | | |

**그림 10.** 본 임상시험 계획도

## 7. 연구대상자

### [1] 피험자의 선정기준

(1) 엉치 부위 욕창 2기(NPUAP 가이드라인에 근거)에 이환된 자

(2) 만 19세 이상의 성인

(3) 본인의 의사 표현에 문제가 없는 자

### [2] 피험자의 제외기준

(1) 임산부 및 수유부

(2) 엉치부위 수술 기왕력이 있는 자

(3) 재발한 욕창

(4) 골염이 있는 자

(5) 내성균으로 격리된 자

(6) 30분 이상 복와위 자세 취할 수 없는 자

(7) 내과적 질환으로 면역억제제 혹은 스테로이드 지속 복용해야 하는 자

(8) 광선 빛에 민감한 체질(광과민증 등)이거나 관련된 약물을 복용하고 있는 자

(9) 기타 연구담당자가 판단하기에 임상시험을 제대로 수행할 수 없다고 판단되는

자

## 8. 예상 연구대상자 수와 산출 근거

### (1) 피험자 수

최종평가 **피험자 군당 16명을 목표**로 임상시험을 진행하고, 중도 탈락률 15%를 고려하여 **대조군 및 실험군 당 19명씩, 총 38명을 대상**으로 함.

### (2) 산정근거

본 연구는 **탐색적 임상시험**으로써, **확증 임상시험의 통계적 검증법 위한 산출근거를 마련하기 위한 연구**임. 2020년도 발표된 Francislene FCP 등의 욕창치료에 있어 광생물학적 치료의 효과에 대한 체계적 리뷰에 의하면 5개의 리뷰된 실험은 각각 군당 6명에서 40명으로 평균 15.8명이었음. 이에 본 실험은 군 당 16명을 최소 기준으로 삼고, 예측되는 중도 탈락률 15%로 선정함. 이에 **최종평가에는 피험자 군당 19명이 목표**임.

**중도 탈락률의 선정 근거**로는, **최근 2년 동안** 본원 단일 교수(임상연구책임자 : 성형외과 임남규 교수)가 치료한 250명의 환자 중 재원기간 내 사망자가 45명으로 **18%의 치사율**을 보였음. 상대적으로 중등도 이상의 욕창 환자에서 치사율이 높은 바, **본 연구에 포함되는 피험자의 경우 치사율은 이보다 낮을 것으로 예측**하여 15%의 중도 탈락률을 설정함.

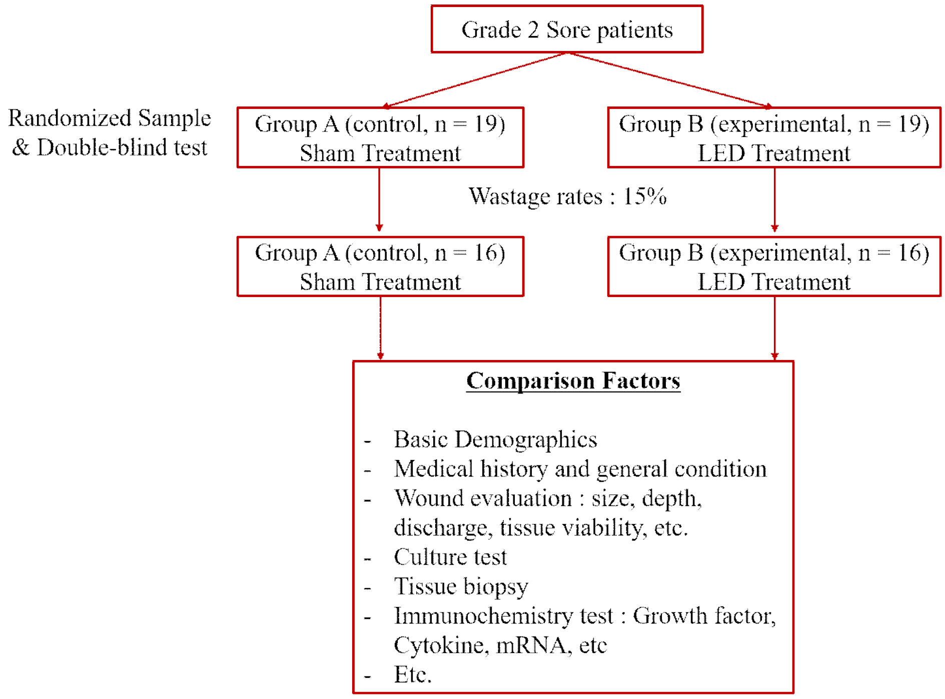


**그림 11.** 본 임상시험 모식도

## 9. 연구대상자 모집

욕창 치료를 위해 성형외과에 의뢰된 외래 및 입원환자 중 선정 및 제외기준을 적용하여 연구 대상에 해당하는 환자에게 연구에 대한 설명을 시행한 후 이에 동의한 환자를 연구에 포함시킴.

## 10. 연구대상자 동의

임상시험 동의서 별도 첨부

## 11. 연구방법

- **임상시험 방법**

본 연구는 본인의 자발적 의지에 의해 임상시험 동의서에 서명한 피험자를 대상으로 임상시험 계획서에 따라 필요한 검진 및 검사를 실시한 후, 선정기준 및 제외기준을 검토하여 본 임상시험에 적합하다고 판단되면 임상시험일정 및 방법 등을 교육함. 피험자의 엉치 부위 욕창에서 15cm 떨어진 위치에서 4가지 파장의 LED를 최대 출력인 5단계[1단계(12mW/$cm^2$), 2단계(27.9mW/$cm^2$), 3단계(44.6mW/$cm^2$), 4단계(61mW/$cm^2$), 5단계(90mW/$cm^2$)]으로 최대 시간(25분)을 조사합니다. LED 조사는 주 3회 시행하며, 한 주의 치료가 끝난 후에는 이학적 검사와 환부 사정을 시행합니다. 이를 총 4주간 수행할 예정임.

이의 유효성 평가를 위해서 1차 척도로 창상 크기 및 재상피화 정도를 파악하며, 2차 척도로 조직 검사에서의 면역화학적 지표를 확인함. 안정성 평가를 위해서는 신체검진 및 활력징후 파악과 더불어 이상사례 조사를 수행할 예정임.

욕창의 보존적 치료 시 통상적으로 수행되는 항생제 치료 및 창상 소독은 기본적으로 시행되며, 이에 임상시험에 참여할 경우 추가적으로 광선 치료를 받게됨. 해당 광선는 4가지 파장이 복합적으로 조사되며, 살균 (BLUE 파장) 및 조직 재생 (AMBER 및 RED 파장)에 유리할 것으로 예측함.

- **임상시험에 사용되는 기기에 관한 정보 및 시험군 또는 대조군에 무작위 배정될 확률**

본 의료기기제품은 2등급 의료용 조합 자극기로써 저출력광선조사기(에너지밀도 20J/$cm^2$ 또는 2W/$cm^2$ 미만의 출력광선)과 적외선조사기가 조합된 발광 다이오드(LED : Light Emitting Diode) 기구임. 조사부는 약 336 × 354 × 105 mm 크기로, 연결된 헤드 연결부에서 각도와 방향이 조절 가능하며, 본체부에서 제품의 높낮이를 조절할 수 있어 원하는 위치에 수월하게 적용 가능하도록 설계되었음.

본 임상시험은 대조기기(Sham 기기)를 이용한 이중 눈가림, 무작위 배정, 평행

설계의 전향적 탐색 임상시험으로써, 의료기기 외에 동일한 외형의 대조기기가 대조군 환자에게 적용됨. 대조기기의 광선 조사부는 12mW/cm$^2$ 이하의 LED를 이용하여 시험기기에서 조사되는 조사부와 유사하게 보이도록 제작됨. 환자 동의시, 무작위로 시험군 또는 대조군 한 쪽 의료기기에 50%의 확률로 배정됨.

- **무작위 배정방법 (Randomization)**

본 임상시험은 피험자 선정 및 제외기준을 만족하고 시험에 참여하기로 동의한 피험자가 최종 선정되면 피험자 식별코드를 부여받음. 피험자 등록번호에 따른 시험군과 대조군의 배정순서는 통계 프로그램인 'R'을 이용하여 블록 무작위 배정방법으로 미리 지정한 블록의 크기를 고려하여 충분한 크기의 무작위 배정번호를 부여함. 무작위 배정표가 작성되면 이는 제3의 독립된 무작위배정 관리 담당자가 독립적으로 관리함.

- **이중 눈가림 (Blinding)**

1) 맹검 주체 : 피험자, 시험자

2) 맹검 방식

  - 맹검 유지를 위하여 맹검 번호는 제3의 독립된 무작위배정 관리자가 관리하며, 임상시험용 의료기기는 독립된 무작위배정 관리자의 지시에 따라 재포장 및 맹검 번호 라벨링하여 임상시험기관으로 전송함.
  - 피험자 눈가림방법 : 시험기기와 대조기기의 외관을 동일하게 하여 피험자가 어느 군에 속하였는지 모르도록 하고 의료기기 관리자는 어떤 기기가 배정되었는지 피험자에게 알리지 않음.
  - 시험자 눈가림방법 : 임상시험용 의료기기는 제3의 독립된 무작위배정 관리 담당자의 지시에 따라 맹검번호를 라벨링하여 임상시험기관으로 전송하며, 의료기기 관리자는 라벨링을 확인하여 피험자에게 배정된 기기를 사용하도록 하고, 이를 시험자에게 알리지 않음.

3) 맹검 해제

  - 맹검시험에는 두 세트의 응급코드 해제표가 제공되는데, 한 세트는 임상시험연구기관에서 보관하고 나머지 한 세트는 의료기기 관리자에게 보내짐.
  - 의료기기 관리자는 각각의 독립된 봉투 안에 각 피험자의 맹검코드를

봉합하여 시험책임자에게 전달함.

- 맹검코드해제용 표는 쉽게 떼어내어 제거할 수 있는 겉표지로 봉함되어 있음. 응급상황이 발생한 경우, 해당 피험자에게 사용중인 의료기기를 식별하기 위해 그 겉표지를 떼어낼 수 있음.
- 겉표지는 응급상황에서만 제거되어야 함. 임상시험 책임자가 그 겉표지를 떼어내어 제거한 경우, 그 시험책임자는 맹검코드 해제일자 및 시간, 구체적인 사유를 기록하고 이 내용을 증례기록서의 '종결'란에 기재해야 함.
- 또한 시험책임자는 즉시 이 맹검코드 해제에 대해 모니터 요원과 수탁기관에 알려야 함.

## ● 임상시험 진행 일정표

| 방문 | 스크리닝 | 치료기 | | | | 추적관찰 | |
|---|---|---|---|---|---|---|---|
| | Visit 1 | Visit 2~4 (1주차) | Visit 5~7 (2주차) | Visit 8~10 (3주차) | Visit 11~13 (4주차) | Visit 14 | Visit 15 |
| 시점 (±2 days) | D0 | D1,D3,D5 | D8,D10,D12 | D15,D17,D19 | D22,D24,D26 | D29 | D210 |
| 임상시험 설명 | ○ | | | | | | |
| 피험자 동의서 취득 및 스크리닝번호 부여 | ○ | | | | | | |
| 적합성 판정 (선정/제외기준) | ○ | | | | | | |
| 인구학적 조사 | ○ | | | | | | |
| 병력 조사 (과거력, 수술기왕력, 약물투여력) | ○ | | | | | | |
| 임신검사 (가임기 여성에 해당) | ○ | | | | | | |
| 이학적 검사 및 활력징후 | ○ | ○ | ○ | ○ | ○ | ○ | ○ |
| 환부사정 및 Braden scale 측정 | ○ | ○ | ○ | ○ | ○ | ○ | ○ |
| 실험실 검사1 (혈액검사, 소변검사) | ○ | | | | | ○ | ○ |
| 실험실 검사2 (골반 X-ray 검사) | ○ | | | | | | |
| 의료기기 적용 | | ○ | ○ | ○ | ○ | | |
| 실험실 검사3 (균 배양 검사, 조직 생검) | | ○ | | | | ○ | ○ |
| 선정기준/제외기준 재확인 | | ○ | | | | | |
| 적합성재판정 및 피험자등록번호 부여 | | ○ | | | | | |
| 이상사례 조사 | | | | | | ○ | ○ |

- **관찰검사방법**

- **스크리닝방문 (Visit 1, D0)** : 이 시험에 참가하도록 선택된 피험자는 시험에 대한 설명을 듣고 다음 순서에 따라 평가를 받음.

  ① 피험자를 시험에 참가시키기 전에 시험과정을 설명하고, 피험자에게 서면 동의서를 받음.

  ② 피험자는 순서대로 스크리닝 번호를 시정 받음.

  ③ 피험자의 인구학적 정보와 병력을 조사함.

  ④ 가임기 여성의 경우 임신 검사를 시행함.

  ⑤ 이학적 검사 및 활력징후를 실시함.

  ⑥ 환부사정 및 Braden scale을 측정함.

  ⑦ 혈액검사, 소변검사, 골반 X-ray 검사를 시행함.

- Visit 2 (D1 ± 2 days)

  ① 광선 조사 전 선정기준 및 제외기준을 재확인한 후 적합성재판정 및 피험자등록번호 부여함.

  ② 이학적 검사 및 환부 사정 시행함.

  ③ 균 동정 검사 및 조직검사 시행함.

  ④ 광선 치료 시행함.

- Visit 3 (D3 ± 2 days) ~ Visit 13 (D26 ± 2 days)

  ① 이학적 검사 및 환부 사정 시행함.

  ② 광선 치료 시행함.

- Visit 14 (D29 ± 2 days)

  ① 이학적 검사 및 환부 사정 시행함.

  ② 혈액검사, 소변검사, 균 동정 검사 및 조직검사 시행함.

③ 광선 치료기 직후 이상 반응 여부 확인함.

- Visit 15 (D210 ± 2 days)

① 이학적 검사 및 환부 사정 시행함.

② 혈액검사, 소변검사, 균 동정 검사 및 조직검사 시행함.

③ 추적 관찰 도중 이상 반응 여부 확인함.

## 12. 관찰 항목

- **관찰항목 및 임상검사항목**

- **환자 동의, 스크리닝 번호 부여 및 인구학적 조사** : 임상시험에 들어가기 전, 본 임상시험의 목적과 내용에 대하여 피험자에게 상세히 설명하고, 서면으로 동의를 받고, 서면동의를 받는 순서에 따라 스크리닝 번호를 부여한 후 인구학적 정보를 조사함. 기록사항은 서면 동의 여부 및 동의 일자, 피험자 이니셜, 성별, 생년월일, 주소 및 연락처 등임.

- **적합성 판정 및 피험자 등록번호 부여** : 피험자 선정기준의 모든 항목이 '예'이고 피험자 제외기준의 모든 항목이 '아니오'인 경우 적합한 피험자로 판정하고 피험자 식별코드를 부여함.

- **병력 조사** : 스크리닝 방문 시에 문진과 과거 진료 기록 점검을 통하여 피험자의 병력에 대해 상세히 조사하여 기록함. 당뇨, 고혈압 등의 과거력, 수술기왕력, 약물 투여력 등이 포함됨.

- **이학적 검사** : 이학적 검사는 매 방문 시 시행되며, 검사 상 발견된 유의할 만한 사항은 증례기록표의 이학적 검사란에 기록하고, 시험의 개시 후 이상반응의 정의에 부합하는 이학적 소견 상의 유의할 만한 사항이 발견된 경우에는 이상반응 증례기록표에 기록함. 단, 시험의 개시 이전에 발현된 바람직하지 못한 이학적 증상의 경우에는 현 병력 조사란에 추가하여 기입하도록 함.

- **활력징후** : 체온, 혈압(수축기, 이완기), 맥박수 등 피험자의 활력징후를 매 방문 시 확인함.

- **환부 사정** : 연구원이 매 치료 방문시마다 욕창의 크기(가로세로깊이), 색깔, 괴사조직 유무, 분비물 양상, 냄새, 미세혈관 출혈, 육아조직 형성 정도 등을

판단함.

- **Braden Scale 측정** : 욕창 위험도를 파악하는 지표로, 6개 항목(감각 인지, 피부 습도, 활동 정도, 체위 변경, 영양, 마찰 및 전단 정도)으로 나누어 점수를 측정함.(각 항목당 1~4점이며, 최대 24점.)

- **기타 검사**

  - **임신검사** : 피험자 선정, 제외기준 부합여부를 확인하기 위해 가임기 여성에 대하여 스크리닝방문에서 임신검사를 실시하며 임신 여부를 증례기록표에 기록함.

  - **실험실검사1** : 피험자 선정, 제외기준 부합여부를 확인하기 위하여 실험실 검사를 수행하며 검사항목은 다음과 같음. 해당 검사가 2주 이내 수행되었을 경우 갈음할 수 있음.

    - ✓ **혈액검사** : CBC, ESR, CRP, 생화학검사 등의 검사를 스크리닝방문 시에 실시하여 검사치 정상여부 및 임상적 의미를 증례기록표에 기록함.

    - ✓ **소변검사** : 스크리닝방문 시에 실시하여 검사치 정상여부 및 임상적 의미를 증례기록표에 기록함.

  - **실험실검사2** : 피험자 선정, 제외기준 부합여부를 확인하기 위하여 골반 X-ray를 촬영하여 골염 여부를 확인함.

  - **실험실검사3** : 임상시험 치료 직전(D1), 임상시험 치료 직후(D29), 임상시험 치료 후 장기 경과관찰 시(D210) 균 동정 검사 및 조직검사 시행하여 변화 과정을 관찰함.

    - ✓ **균 동정 검사** : 조직 검체로 균 동정 검사 시행함. 검사 결과에 약 7일 소요됨.

    - ✓ **조직검사** : 창상의 조직 변화 과정을 조직검사 통하여 파악함과 동시에 면역화학적 검사(IL-1, 4, 6, 10, 13, TNF-a, TGF-b, MMP-1, 2, TIMP, Colla1, 3 등)를 통하여 창상 회복과정을 파악함.

    - ✓ **해당 검사를 위해서는 3~4mm punch biopsy를 이용하여 진행하며, 동의를 구한 자에게만 실시**하도록 함.

    - ✓ **해당 검사는 침습적이나 만성 감염성 창상인 욕창 치료에 있어 필수 검사**이며, **이의 검사가 욕창 회복의 지연이나 환자의 해로움에 영향**

**을 끼치지 않음**. 다음 예시는 균 동정 배양 검사 및 조직검사를 시행한 욕창 환자의 창상 회복 과정을 보여주며, 환자 창상 회복에 있어 해당 검사가 해롭지 않음을 증명함.

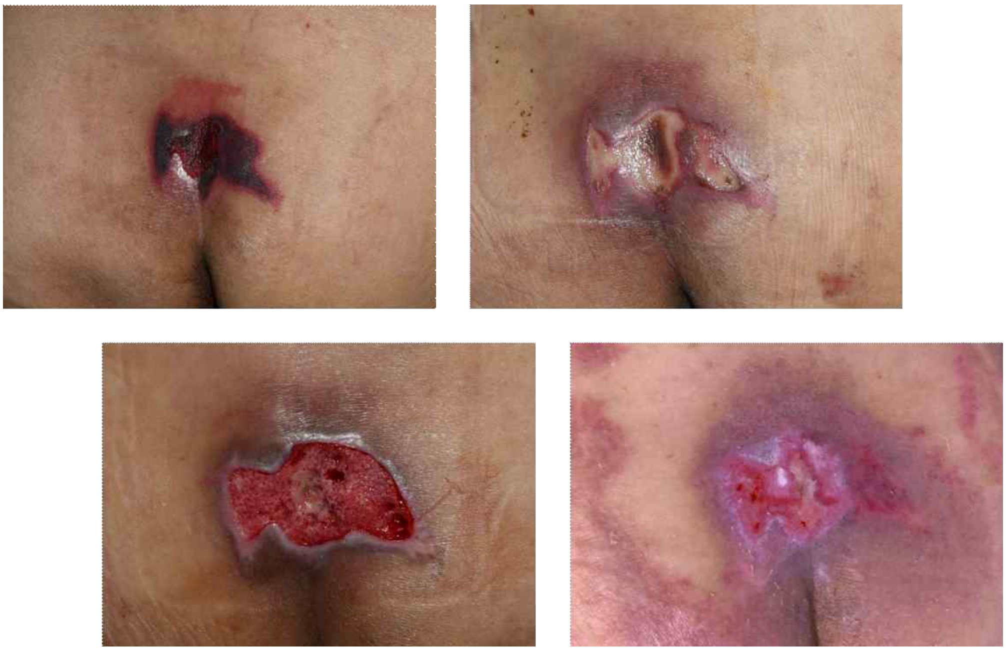

**그림12.** 균 동정 배양 검사 및 조직검사 시행한 욕창 환자의 자연적 창상회복 과정 예시

- **이상반응 확인** : 이상반응에 대한 정보는 수시로 피험자에게 자발적인 보고를 하도록 교육하며, 시험담당자는 문진을 통하여 이상반응 발현 여부를 확인함. 이상반응 발현 시 발현일 및 소실일, 이상반응의 정도 및 결과, 광선조사와 관련하여 취해진 조치 및 광선 조사와의 인과관계, 광선 조사 이외 의심되는 약제명이나 처치, 이상반응에 대한 치료 여부 및 내용 등을 증례기록지에 상세히 기록함.
- **본 연구의 예상되는 이상반응** (Chen C. 등의 2014년도 코크란 리뷰에 의거함)
  - 경증 : 경한 통증, 경한 발적 등
  - 중등증 : 중등 통증, 중등 발적, 경도 삼출물 등
  - 중증 : 세균 번식, 창상 출혈, 조직 괴사 등

## 13. 효과 평가 기준 및 방법 (자료 분석 및 통계적 방법 포함)

- 1차 유효성 평가 척도 : <u>창상 크기 및 재상피화 정도</u>

  - 0주와 4주 후 대조군과 실험군의 창상 크기 및 재상피화 정도를 시간대비하여 각각 산출하여 두 군 간의 차이를 paired T-test를 이용하여 분석

  - 창상 크기 및 재상피화 정도는 실험 방문일에 연구자가 이학적 검사를 통하여 욕창 크기(가로×세로×깊이 : 자를 이용하여 cm 단위로 측정) 측정함.

- 2차 유효성 평가 척도 : <u>조직 검사에서의 면역화학적 지표</u>

  - <u>Pro-inflammatory cytokine (IL-1, 6) / Anti-inflammatory cytokine (IL-4, 10, 13) 비율</u>을 구한 후 0주와 4주 후 대조군과 실험군을 비교함으로써 증식기로의 진행 과정을 분석함.

  - 면역화학적 지표는 실험실에서 연구원이 RNA sequencing을 통하여 측정함.

## 14. 안전성 평가 기준 및 평가 방법

- **평가기준**

  - 의료기기 사용 시 또는 사용 후 발생한 이상 반응에 대한 피험자의 호소증상 : 엉치 부위의 자극 증상 (따가움, 두드러기, 염증, 가려움 등) 여부

  - 이학적 검진 : 엉치 부위의 피부 병변 (홍반, 부종 등) 여부

  - 추적관찰 기간 : 연구 종료 후 6개월 후까지 추적관찰 진행 예정.

  - 경증(mild) : 피험자의 정상적인 일상생활을 저해하지 않고, 최소한의 불편을 야기하며 피험자가 쉽게 견딜 수 있는 경우, 처치가 간증하며 완전한 회복으로 의료기기의 유효성을 미치지 않는 경우, 의학적 소견 정도와 임상용 의료기기와의 인과관계를 평가한다.

  - 중증도(moderate) : 피험자의 정상적인 일상생활을 유의하게 저해하는 불편을 야기하는 경우, 지속적인 처치로 회복이 가능하며 임상시험용 의료기기의 유효성을 미치지 않는 경우, 의학적 소견정도와 임상용 의료기기와의 인과관계를 평가한다.

  - 중증(severe) :피험자의 정상적인 일상생활을 불가능하게 하는 경우, 처치가 불가능하며, 연구용 임상의료기기가 유효성에 영향을 미치는 경우, 의학적소견정도와 연구용 임상의료기기와의 인과관계를 평가한다.

- **평가방법**

- 피험자에 의해 보고되거나 이학검진에 의해 판명된 이상사례에 대하여 중증도와 유형 및 시험군과 대조군의 이상사례 발생률을 평가하기 위한 분석을 수행함.

- **부작용 보고**

1) 부작용의 정도 : 경증, 중등증, 중증 등을 이상사례 기록표에 표시할 수 있도록 함.

2) 임상시험용 의료기기와의 인과관계 : 평가기준에 따라 인과관계를 판정하여 이상사례 기록표에 표시할 수 있도록 함.

3) 처치 : 이상사례로 인하여 사용방법변화, 사용횟수 감소, 사용중단 등 임상시험방법의 변경이 있는 경우 기록표에 표시할 수 있도록 함.

4) 처치경과 : 이상사례 발현 후 처치에 따른 이상사례의 소실, 악화 등을 기재할 수 있도록 함.

5) 임상시험담당자의 견해 : 이상사례에 대한 시험 담당자의 견해를 기록할 수 있도록 함.

- **보고방법** : 본 내용은 의료기기 임상시험 관련 규정 및 의료기기 임상시험기관에서 규정하고 있는 표준작업지침서에 따라 보고함.

1) 이상사례는 임상시험 중 피험자에서 발생한 모든 의도하지 않은 증후, 증상, 질병을 말하며, 이상사례에 대한 의학적 소견 및 정도와 임상시험용 의료기기와의 인과관계를 평가하여 증례기록서에 기록하여야 함. 따라서 이상사례에 대한 임상시험용 의료기기와의 인과관계에 대한 평가기준이 제시되어야 함.

2) 안전성 평가기준은 예측되는 부작용 및 이상사례가 발생하면 중증도를 평가하여 증례기록서에 기록하여야 하므로 그 평가기준을 말함.

3) 안전성 평가방법은 부작용, 의료기기이상반응, 임상시험용 의료기기와의 연관성이 있는 이상사례의 발현빈도 등에 대한 시험군과 대조군의 비교 평가를 위한 통계분석방법과 평가기준을 제시함.

4) 부작용 보고 : 시험책임자는 모든 중대한 이상사례를 즉시 의뢰자에게 알려야

하고, 계획서에 따라 문서로 상세한 내용이 포함된 추가 보고를 하여야 함. 사망을 초래하거나 생명을 위협하는 경우에는 의뢰자가 이 사실을 보고 받거나 알게 된 날로부터 7일 이내에, 이 경우 의료기기이상반응에 대한 상세한 정보를 최초 보고일로부터 8일 이내에 추가로 보고하여야 함. 그 밖에 중대하고 예상하지 못한 의료기기이상반응의 경우에는 의뢰자가 이 사실을 보고받거나 알게 된 날로부터 15일 이내에 식약처장에게 각각 보고하여야 함.

## 15. 예측 부작용 및 주의사항과 조치

- **예측되는 부작용**

- 염증 반응 촉진으로 인한 삼출물 증가, 세균 번식, 창상 출혈, 조직 괴사 등의 부작용이 발생 가능함.
- Chen C. 등의 2014년도 코크란 리뷰에 의하면 광생물학적 치료로 통증, 미세출혈, 발적 등의 부작용 또한 발생 가능함.
- 부작용이 있는 경우, 부작용 명과 증상의 정도를 증례기록지에 기록함.

- **사용 시 주의사항**

- 광선 빛을 눈에 직접 비추지 말 것. 광선 빛을 자기 눈에 비추거나, 다른 사람의 눈에 비추거나, 확대경을 통하여 비추거나, 거울을 통해 반사된 빛을 보는 등의 행위를 할 경우 눈에 일시적인 장애가 올 수 있으며, 장기간 노출 시에는 눈에 해로움.
- 광선 빛에 민감한 체질의 사람이거나, 관련된 약물을 복용하고 있는 사람은 본 제품에 해로울 수 있으므로 전문가와 상담 후 사용할 것.
- 본 제품을 사용할 때는 반드시 사용설명서에 기재된 지침에 따라야 함.

## 16. 중지 및 탈락기준

- **중지기준**

- 임상시험 진행 중 부작용, 이상반응 등의 관찰되는 상황이 임상시험을 계속 진행하는 것에 무리가 있다고 판단되는 경우에는 임상시험책임자는 임상시험심사위원회에 임상시험 중지요청을 하여야 하고, 임상시험심사위원회의 결정

에 따라 임상시험을 중지할 수 있음.

- 임상시험의뢰자는 임상시험용 의료기기의 안전성 등의 사유로 임상시험을 중지하고자 할 경우 임상시험심사위원회에 임상시험 중지요청을 하여 임상심사위원회의 결정에 따라 임상시험을 중지할 수 있음.

● **탈락기준**

- 임상시험에 참여하는 모든 피험자의 시험 완료 여부를 기록하고, 광선 조사나 관찰이 중단된 경우에는 그 이유를 기록함. 임상시험이 진행 중인 피험자에 대하여 시험을 중단할 수 있는 경우는 다음과 같음.
  - ■ 선정기준 및 제외기준에 위배된 경우
  - ■ 치료기간에 금지된 약물을 사용한 자 : 스테로이드, 면역억제제
  - ■ 피험자에게 중대한 이상반응이 발생한 경우 혹은 이상반응으로 인하여 피험자가 시험 중단을 요구하는 경우, 이상반응으로 인하여 시험자가 시험 중단이 필요하다고 판단되는 경우
  - ■ 임상시험 전 검사에게 발견치 못한 전신질환이 발견된 피험자
  - ■ 임상시험 기간 중 만족스럽지 못한 치료 효과로 인하여 피험자 또는 피험자의 법정 대리인이 시험 중단을 요구하는 경우
  - ■ 시험자나 피험자에 의해 시험계획을 위반하는 경우
  - ■ 피험자가 임상시험 참가 동의를 철회한 경우
  - ■ 피험자의 추적이 안되는 경우
  - ■ 피험자에게 광선 조사하는 데 문제가 발생한 경우
  - ■ 기타 즉각적인 치료를 요하는 질환의 발생으로 담당자의 판단에 의해 시험의 진행이 적합하지 못하다고 판단되는 경우

● **중지 및 탈락처리**

- 임상시험기간 중 피험자의 중지 혹은 탈락이 발생할 경우, 해당 사항을 증례기록서에 기록하고, 발생할 수 있는 이상반응에 대해 문진이나 전화방문을 통해 추적 관찰함.

- 중도 탈락된 자는 타당한 이유 또는 근거가 없는 한 안전성 및 유효성 평가 통계 처리에 포함되며, 결측치에 대해서는 결측 직전의 값으로 대체하는 LOCF(Last Observation Carrying Forward) 방법으로 분석을 실시함.

## 18. 연구대상자의 위험과 이익

경도 욕창의 경우 보존적 치료를 수행하며, 항생제 치료 및 창상 소독과 더불어 압박 제거를 위한 체위 변경이 중요한 치료입니다. 창상 소독 제재의 종류가 많으나 해당 제품마다 가지고 한계가 있어 아직까지 임상적으로 정립된 소독 방법은 없습니다. 만일 본 임상시험에 참여하지 않기로 결정한다면 통상적인 보존적 치료를 받을 것이며, 임상시험 도중에도 광선 치료 거부 의사를 밝힐 수 있으며, 그 즉시 통상적인 보존적 치료를 받을 수 있을 것입니다. 따라서 피험자가 임상시험 참여 여부와 상관없이 질환의 회복을 위해 노력할 것이며, 피험자의 위해가 최소화하도록 노력할 것입니다.

본 임상시험 의료기기의 예상되는 부작용은 엉치부위의 자극 증상 (따가움, 두드러기, 염증, 가려움 등)이 발생할 수 있으며, 엉치 부위의 홍반 및 부종 등이 나타날 수 있습니다. 이상 증세가 나타나는 즉시 해당 증세에 적합한 치료를 수행할 예정이며, 본 임상시험과 관련된 손상이나 피해가 발생될 경우에는 전적으로 임상시험 의뢰자((주)링크옵틱스)가 법적인 책임을 부담하며 피해를 보상할 것입니다. 하지만, 임금손실과 같은 기타 상해 관련 또는 질병 관련 비용은 보상하지 않습니다.

본 임상시험에 참여함으로써 기대되는 이익은 연구에 참여하는 동안 연구와 연관된 혈액 검사, 소변 검사, 방사선 검사, 조직검사, 균 동정 검사를 연구비에서 지원하는 것입니다. 이 연구수행과 무관한 입원비 및 검사비와 진찰료는 귀하가 부담하게 됩니다.

## 19. 연구대상자 안전대책 및 개인정보 보호대책

- **피해자 보상에 대한 규약 (별첨 첨부)**

본 임상시험과 관련된 손상이나 피해가 발생될 경우에는 **전적으로 임상시험 의뢰자 ((주)링크옵틱스)가 법적인 책임을 부담하며 피해 보상할 것**이며, 부작용 및 질환 악화의 경우는 이미 알려진 치료 방법으로 치료 받게 됨. 그러나, **임금손실과 같은 기타 상해 관련 또는 질병 관련 비용은 보상하지 않음.** 연구 담당의사 및 연구진이 그 밖의 가능한 모든 재정적 보상에 대한 추가 정보를 제공할 것임.

① 일시적 통증 또는 쉽게 치료될 수 있는 정도의 손상으로서 시험기관이 이에 대한 치료가 필요하다고 판단하는 경우(보상범위는 필요한 치료비에 한정함)

② 입원 또는 입원 기간의 연장이 필요한 경우

③ 지속적 또는 의미 있는 불구나 기증 저하를 초래하는 경우

④ 선천적 기형 또는 이상을 초래하는 경우

⑤ 사망을 초래하거나 생명을 위협하는 경우

**- 임상시험 후 피험자의 진료에 관한 사항**

임상시험에서 탈락되거나 반응이 없는 환자는 다른 적절한 치료를 받을 수 있도록 지도하며, 임상시험이 종료된 환자는 이후의 진료에 관하여 해당 병원의 치료절차에 따르며, 이후의 치료비는 피험자가 지출하여야 한다. 다만, 부작용 발생시, 임상시험에 사용된 임상의료기기와의 인과관계 유, 무를 확인한 후, 본 임상시험에 사용된 임상의료기기로 인하여 부작용이 발생한 경우 의뢰자에게 치료에 관한 부작용이 소실될 때까지 치료비를 지불한다.

**- 피험자의 안전보호에 관한 대책**

● **임상시험 실시기관**

임상시험 실시기관의 장은 해당 임상시험의 실시에 필요한 임상시험실, 설비와 전문인력을 갖추어야 하고, 긴급 시 필요한 조치를 취할 수 있도록 하는 등 해당 임상시험을 적절하게 실시할 수 있도록 하여야 함.

● **임상시험심사위원회(Institutional Review Board : IRB)**

IRB는 국내법규 및 관례에 따라 구성되어 있어야 하며, 피험자의 권리, 안전, 복지를 보호해야 함. 또한 취약한 환경에 있는 피험자가 임상시험에 참여하는 경우에는 그 이유의 타당성을 면밀히 검토하여야 함. IRB는 임무를 수행함에 있어 피험자의 시험 참가 동의가 적절하게 얻어지지 않았거나 임상시험이 임상시험 계획서에 따라 진행되지 않은 경우 또는 심각한 이상반응 혹은 이상반응이 나타난 경우에는 임상시험의 일부 또는 전부에 대하여 중지명령 등 필요한 조치를 시험책임자에게 하여야 함.

● **시험자(Investigator)**

1) 시험자라 함은 시험책임자, 시험담당자, 임상시험 조정자를 말함. 시험자는 의뢰자와 합의되고 임상시험심사위원회 및 식품의약품안전처장의 승인을 득한

임상시험계획서를 준수하고 임상시험을 실시하여야 함.

2) 임상시험 중 또는 임상시험 이후에도, 시험자는 임상적으로 의미있는 실험실적 검사치의 이상을 포함하여 임상시험에서 발생한 모든 이상반응에 대해 피험자가 적절한 의학적 처치를 받을 수 있도록 조치하여야 하고, 시험자가 알게 된 피험자의 병발질환에 대해 의학적 처치가 필요한 경우 이를 피험자에게 알려주어야 함.

3) 시험자는 임상시험 계획을 정확히 분석 및 숙지하고, 대상 피험자의 문제점을 적극적으로 대응함.

- **의뢰자**

1) 임상 연구의 계획, 관리, 재정 등에 관련된 책임을 갖고 있는 자로, 통상 의료기기 임상시험의 경우 의료기기 제조업자(수입자를 포함)를 말함.

2) 임상시험대상, 시험방법, 증례보고서의 서식과 내용 등이 임상시험계획서의 절차에 따라 이루어지도록 하여야 함.

3) 의뢰자의 점검 계획과 절차는 임상시험의 중요도, 피험자 수, 임상시험의 종류와 복잡성, 피험자에게 미칠 수 있는 잠재적인 위험의 정도 및 이미 확인된 임상시험 실시 상의 문제점 등에 따라 결정되어야 함.

- **모니터링(Monitoring)**

1) 모니터링이라 함은 임상시험 진행과정을 감독하고, 해당 임상시험이 임상시험계획서, 표준작업지침서, 임상시험 실시기준 및 관련규정에 따라 실시 및 기록되는지 여부를 검토 및 확인하는 활동을 말함.

2) 임상시험에 대한 모니터링은 임상시험 모니터 요원의 정기적인 임상시험 실시기관 방문과 전화 등을 통하여 이루어짐. 방문 시 모니터는 환자 기록원본, 임상시험용 의료기기 관리 기록, 자료 보관 (연구파일) 등을 확인함.

3) 임상시험 모니터 요원은 임상시험 진행 과정을 잘 살피고, 문제가 있을 경우 시험자와 상의함.

- **임상시험계획서의 변경**

1) 임상시험계획서를 임상시험심사위원회 및 식품의약품안전처장으로부터 승인받은 후, 시험 절차가 광범위해지거나 위험도가 높아지거나 피험자 선정기준에 변화가 있거나 추가적인 안전성 정보로 인해 임상시험계획서를 변경하는 경우에는 임상시험심사위원회 및 식품의약품안전처장의 승인을 받아야 함.

2) 임상시험계획서를 수정할 때에는 개정일자, 개정이유, 개정내용 등을 기록하여 보관하여야 함.

3) 시험자는 피험자에게 발생한 즉각적 위험 요소의 제거가 필요한 경우를 제외하고는, IRB 및 식품의약품안전처장의 변경승인 이전에는 계획서와 다르게 임상시험을 실시하여서는 안됨. 만일 피험자에게 발생한 즉각적 위험요소를 제거하기 위해 IRB의 승인을 얻기 전에 이러한 임상시험계획서의 변경을 적용하게 되는 경우, 가능한 한 빨리 변경에 대하여 IRB, 의뢰자, 식품의약품안전처장에게 제출하여야 함. 그리고 IRB 위원장이나 간사가 승인한 문서를 의뢰자에게 보내야 함.

4) 임상시험에 영향을 주지 않는 사소한 수정이나 명시는 승인이 반드시 필요한 것은 아니며 행정상 변경이 필요함.

- **피험자 동의(Informed Consent)**

1) 피험자 동의라 함은 피험자가 임상시험 참여유무를 결정하기 전에 피험자를 위한 설명서를 통해 해당 임상시험과 관련된 모든 정보를 제공받고, 서명과 서명 날짜가 포함된 문서를 통해 본인이 자발적으로 임상시험에 참여함을 확인하는 절차를 말함.

2) 피험자 본인 또는 대리인이 동의서 서식, 피험자 설명서 및 기타 문서화된 정보를 읽을 수 없는 경우에는 공정한 입회자가 동의를 얻는 전 과정에 참석하여야 함.

3) 동의를 얻기 전에 시험자는 피험자 또는 대리인이 임상시험의 세부사항에 대해 질문하고 해동 임상시험의 참여 여부를 결정할 수 있도록 충분한 시간과 기회를 주어야 하며, 모든 임상시험 관련 질문에 대해 피험자 또는 대리인이 만족할 수 있도록 대답해 주어야 함.

- **피험자 기록의 비밀보장**

1) 피험자의 신원을 파악할 수 있는 기록은 비밀로 보장될 것이며, 임상시험의 결과가 출판될 경우에도 피험자의 신원을 비밀 상태로 유지함.

2) 본 임상시험에 관련된 의뢰자, 모니터 및 점검자는 본 임상시험의 모니터링과 점검 및 진행사항 관리를 위한 목적으로 피험자의 기록을 열람할 수 있음. 시험자는 본 임상시험계획서에 서명함으로써, 국내의 법규와 윤리적 측면에서 임상시험의뢰자 또는 모니터 및 점검자가 피험자의 차트와 증례기록서 기록을 검증하기 위하여 해당문서를 검토하거나 복사할 수도 있음을 인정함. 이러한

정보들은 기밀로 보관되어야 함.

3) 증례기록서 등 임상시험에 관련된 모든 서류에는 피험자 이름이 아닌 피험자 식별코드(일반적으로 피험자 이니셜)로 기록하고 구분함.

- **기록의 보존**

임상시험 실시와 관련된 각종 자료 및 기록을 잘 보존하여야 하며, 보안을 유지하도록 함. 임상시험 결과보고서 작성 완료 이후에는 임상시험 관련 문서를 임상시험 종료일로부터 10년 간 보존하도록 함.

- **검체의 처리**

- 모든 검체는 코드화하여 개인 식별 불가하도록 처리함.

- 균 동정 검사 검체 : 진단검사의학과에서 검사 수행 및 검체 처리

- 조직검사 검체 : 의과대학 328호 중개임상의학센터에서 면역화학적 검사 시행 후 잔존 검체 발생 시 연구실 내 의료용 폐기물 장소에서 폐기 예정.

**- 그 밖에 임상시험을 안전하고 과학적으로 실시하기 위해 필요한 사항**

- **증례기록서**

1) 본 임상시험에서는 종이형태의 증례기록서를 사용하여 자료를 수집함. 피험자의 근거문서라 함은 시험기관에 보관되는 담당의사의 피험자 기록을 의미함. 대부분의 근거문서는 병원이나 담당의사의 차트이며, 피험자의 증례기록서에 기록된 모든 정보는 해당 근거문서와 일치해야 함.

2) 증례기록서를 기록, 검토, 서명하는 것은 시험책임자의 의무임.

3) 증례기록서 작성 후, 시험책임자는 개개의 증례기록서에 서명하여 증례기록서에 기록된 정보가 사실임을 보증함. 즉, 시험책임자는 증례기록서에 기재된 본 임상시험과 관련된 데이터의 최종적인 책임을 갖음.

- **모니터링**

1) 의뢰사 또는 의뢰사로부터 모니터링 업무를 위탁받은 기관에서는 피험자의 권리와 복지 보호 및 본 임상시험 자료의 질과 신뢰성 확인을 위하여 모니터링을 실시함. 시험의뢰자인 의뢰사는 모니터 요원을 선정하고, 모니터 요원의

명단과 자격에 대한 문서를 갖추고 있어야 함. 또한 모니터 요원은 본 임상시험용 의료기기, 임상시험계획서, 동의서 서식, 피험자 설명서 및 그 밖의 피험자에게 제공되는 서면정보, 시험의뢰자 표준작업지침서와 관련 법령 등 모니터링에 필요한 과학적 또는 임상적으로 충분한 지식을 가져야 함.

2) 모니터 요원은 임상시험 관련 자료가 근거문서와의 대조 시 정확하고 완전하며 검증이 가능한지, 그리고 본 임상시험이 승인받은 시험계획서와 의료기기 시행규칙 제24조(임상시험 실시기준 등) 등 관련 규정에 따라 수행되는 지 여부를 확인함.

3) 시험책임자와 시험담당자는 의료기기임상시험관리기준에 정의된 것과 같이 모니터 요원이 증례기록서에 기입된 자료들을 확인할 수 있는 자료들(Source document : 병원 또는 개인 차트, 실험실 결과 기록, 예약 기록 등)을 볼 수 있도록 함.

- **자료의 기록 및 보관**

1) 임상시험 실시와 관련된 각종 자료 및 기록을 잘 보존하도록 보관하는 장소가 따로 준비되어 있어야 하며, 보안이 유지하도록 함.

2) 결과보고서 작성 완료 이후에는 보관책임자를 정하여 임상시험 관련 문서를 임상시험 종료일로부터 3년간 보존하도록 함.

- **보고서 제출 및 출판**

1) 시험책임자는 시험의뢰자 또는 시험기관과 의논하여 본 임상시험의 내용에 대한 보고서를 작성함.

2) 임상시험 결과로부터 나오는 출판물에는 시험책임자 및 시험담당자 등의 참여가 명시되어야 하며, 저자의 서열에 관한 계약은 원고를 작성하기 이전에 이루어져야 함.

3) 임상시험에 관련된 모든 요약, 원고 또는 발표물들은 출판 또는 발표 전에 시험의뢰자 또는 시험기관에 제공되고 검토되어야 함.

4) 시험기관에 의하여 기밀사항이라고 판단되는 모든 자료는 삭제해야 함. 이때, 본 임상시험에서 얻어진 결과는 기밀사항으로 간주하지 않음.

- **계약서**

  - 시험의뢰자는 임상시험기관의 장과 임상시험계약을 체결하여야 하며, 계약서에는 임상시험의 재정에 관한 사항, 업무의 위임 및 분장에 대한 사항 및 시험의뢰자와 임상시험기관의 장의 의무사항을 포함함.

## 20. 참고문헌


1) NPIAP-EPUAP-PPPIA. Prevention and Treatment of Pressure Ulcers/Injuries: Clinical Practice Guideline. The International Guideline 2019

2) Robert K, Juan LR, Jeffery E. Pressure Sores. Neligan 4th edition. Elsevier. 2016 Vol. 4;350-380.

3) William VP, Benjo AD. The National Cost of Hospital-Acquired Pressure Injuries in the US. Int Wound J. 2019;16(3):634-640.4)

4) Chen C, How WH, Chan ESY, Yeh ML, Lo HLD. Phototherapy for Treating Pressure Ulcers. Cochrane Database Syst Rev. 2014;11(7):CD009224.

5) Francislene FCP, Jorge VCF, Hellen R, et al., Effect of Photobiomodulation on Reparing Pressure Ulcers in Adult and Elderly Patients: A systematic Review. Photochem Photobiol. 2020;96(1):191-199.


**[별첨] 피해자 보상에 대한 규약**

- **보상요건**

본 보상규약에 따른 피험자 보상은 다음의 요건 하에 이루어집니다.

① 본 연구용 임상의료기기로 인하여 발생한 신체상의 손상일 것.

② 당해 임상 시험으로 인하여 피험자의 상태가 악화되었을 경우.

③ 시험자가 식품의약품안전청장의 승인을 받은 임상시험계획서의 제반 내용을 준수하였을 것

④ 시험자의 명백한 과실이나 의무태만에 기인하지 아니하였을 것

⑤ 피험자가 시험책임자 또는 시험담당자의 제반 지시사항을 모두 준수하였을 것

⑥ 피험자가 당해 신체상의 손상으로 인한 손해의 발생을 최소화하기 위하여 조치를 하였을 것

● **보상제외 사유**

① 연구용 임상의료기기로부터 기대된 효과, 효능의 불충분으로 의한 손상

② 피험자의 부주의로 인하여 발생한 손상

: 정해진 시간 동안 정해진 신체 부위에 사용하지 않은 경우

: 부작용 증상 발생 시, 상담 및 치료를 요청하지 않은 경우

: 피험자 주의사항을 지키지 않은 경우

**< 피험자 주의 사항>**

1) 광과민증 환자는 사용해서는 안된다.

2) 광과민증을 증가시키는 약물과 음식물은 병용할 경우 기기를 사용한 부위에 화상과 같은 손상을 입힐 수 있어 주의가 필요하다.

- 광과민증을 일으킨다고 알려진 약물 : 퀴놀론계 약물(날리디식산, 오플록사신, 스파플록 사신 등), 테트라사이클린계 약물(염산미노사이클린, 염산독시사이클린 등), 설파제, 그리세오풀빈, 타르제제, 치아짓계 약물(에치아짓, 사이클로벤치아짓, 펜플루티짓 등), 포르피린계 약물(NAPP 등), 페노치아진계 약물(프로클로르페라진, 클로르프로마진 등), 메칠렌블루 등

- 광과민증을 증가시키는 음식물

: 푸로쿠마린을 함유한 음식물 (샐러리, 라임, 당근, 파세리, 무화과, 겨자 등)

3) 임신 중이거나 수유중인 여성은 사용을 금지한다.

4) 제품을 과도한 습도, 과열, 과냉에 노출시키면 안된다.

5) 제품이 젖어있거나 물이 새고 있는 상태에서는 사용해서는 안된다.

6) 제품이 과열되므로 30분 이상 연속적으로 사용해서는 안된다.

7) 광조사부가 심하게 오염되면 제품 효율이 떨어지므로 마른 수건으로 오염물질 제거 후, 사용한다.

8) 제품 동작 시, 눈으로 점등된 광 조사부를 환자가 바라보면 안된다.

9) 상처가 있는 부위에 사용해서는 안된다.
10) 전기적 충격을 예방하기 위하여 자석 또는 전류가 흐르는 물체와의 접촉을 피하여 보관하여야 한다.
11) 사용자 임의대로 제품을 개조, 분해, 수리해서는 안된다.
12) 제품 사용 중 불편하다고 느낄 경우에는 사용을 중단한다.

● **보상기준**

① 예상된 의료기기 이상반응에 대하여 당사자들 간에 미리 합의한 보상액 또는 조치가 있는 경우, 당해 기준에 따라 이를 보상합니다.

② 그 외에 경우에는 신체손상의 정도, 성격, 지속기간, 유사사례 등을 종합적으로 고려하여 당사자들 간에 합의한 보상방법에 따라 이를 보상합니다.

③ 당사자들 간에 전항의 합의가 이루어지지 아니한 경우에는, 법원의 판결 및 이에 준하는 결정의 확정내용에 따라 보상합니다.

● **보상절차**

① 본 보상규약에 따른 신체상의 손상을 입은 피험자는 임상시험의 시험책임자나 시험기관에 먼저 필요한 의료조치를 요청하여야 합니다.

② 시험책임자나 시험기관의 조치에도 불구하고 신체상의 손상이 완치되지 아니한 피험자는 의뢰기관에 대하여 이에 대한 보상을 요청할 수 있습니다.

③ 의뢰기관은 위 보상요청을 받은 후 지체 없이 보상대상 해당여부 및 보상기준에 대한 조 사를 마치고 이에 관한 내용을 피험자에게 통보하여야 합니다.

④ 피험자는 위 통보내용에 의의가 있는 경우, 위 통보를 받은 날로부터 영업일(5) 이내에 이에 대한 이의내용을 의뢰기관에 통보하여야 합니다.

⑤ 피험자가 제(3)항의 통보를 받고도 이에 대한 이의를 통보하지 아니한 경우, 양 당사자는 위 통보내용에 따른 보상에 합의한 것으로 양해합니다.

⑥ 피험자가 제(4)항의 규정에 따라 이의를 통보한 경우, 의뢰기관은 피험자에게 위 보상 대상 해당여부 및 보상기준에 관하여 판단할 객관적인 전문가를 복수로 추천하고, 피험자를 위 추천일로부터 영업일(3)내에 추천인 1명을 지명합니다. (피험자가 지명하지 않을 경우 의뢰 자가 임의 택일합니다)

● **적용범위**

① 본 보상규약은 의뢰기관이 의뢰하는 모든 임상시험에 참여하는 피험자에 의뢰기관과 피험자 간에 다른 약정이 없는 한 그 범위 내에서 일반적으로 적용됩니다.

② 피험자가 임상시험에 관한 보상에 대하여 의뢰기관의 승인을 받지 아니하고 임상 시험과 관련된 다른 제3자와 체결한 일체의 합의내용은 의뢰기관에 대하여 효력이 없습니다.

폐사는 위의 여러 제반 내용을 참고하여 피험자가 본 임상시험에 의해 어떠한 불이익이라도 받지 않도록 주의하며, 만약 본 임상시험에 의해 문제점이 발생한 경우 피해자 보상 규약에 의거하여 책임질 것을 서약합니다.
