# Supplementary material for: The validity and safety of multispectral light emitting diode (LED) treatment on grade 2 pressure ulcer: Double-blinded, randomized controlled clinical trial
Source: PLoS One. 2024 Aug 23;19(8):e0305616. doi: 10.1371/journal.pone.0305616 (PMC11343461; doi:10.1371/journal.pone.0305616)
Supplement: S14 File — (PDF) [file pone.0305616.s022.pdf]

# Research protocol (for human subjects research)

ver 2.0

| Study Title |
| --- |
| For patients with mild pressure ulcer co-morbidity, medical light<br>To evaluate the safety and efficacy of the irradiator BELLALUX Lite on wound healing, a single-center, double-blind, randomized, parallel design (sham device control) prospective exploratory clinical trial |

## 1. Research Background

Pressure ulcers are localized damage to the skin and subcutaneous fat caused by combined pressure, including shear and friction forces, and are often caused by objects such as bony prominences or medical devices. Studies have reported morbidity rates ranging from 0% to 75%, with an average of 6.3%.[1] Pressure ulcer morbidity is particularly high in intensive care unit patients (8.8% to 12.1%) and those with spinal cord injuries (33% to 60%).[2]

While domestic statistics are not available, U.S. statistics estimate that 2.5 **million people per year suffer from pressure** ulcers, and **60,000 people** per **year die from** them. This is more than the number of deaths from the flu (5.6 million) and suicide (44,000) per year. As a societal problem, pressure ulcers **cost society an estimated $11.6 billion annually**. The cost per person ranges from $500 to $150,000.[1-3]

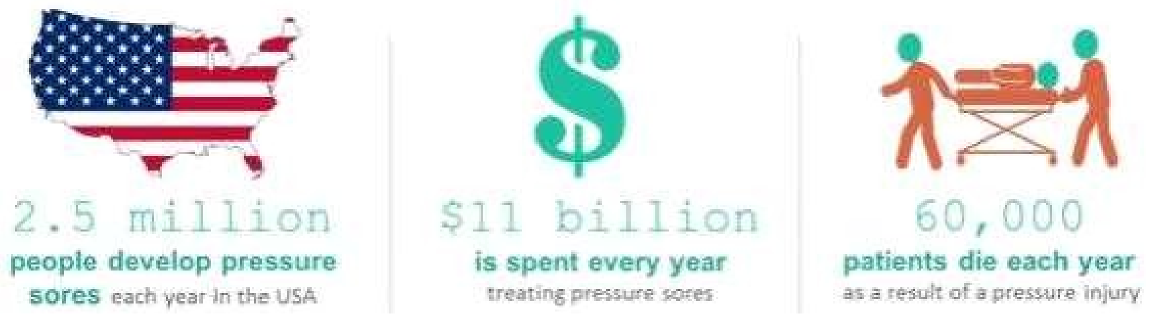


**Figure 1.** Level of morbidity and annual societal cost of pressure ulcers (US statistics)

In general, the average pressure in capillaries is around 32mmHg, and ischemic necrosis occurs when twice this pressure (70~80mmHg) is sustained for more than 2 hours. Doubled pressure acts on the tissue and accelerates the progression of necrosis, especially when accompanied by shear and friction forces. In terms of pathogenesis, pressure ulcers can occur anywhere on the body, and while studies vary, they are most commonly found on the hip (28.3%), heel (23.6%), and sciatic (17.2%).[2]

---

1) NPIAP-EPUAP-PPPIA. Prevention and Treatment of Pressure Ulcers/Injuries: Clinical Practice Guideline. The International Guideline 2019

2) Robert K, Juan LR, Jeffery E. Pressure Sores. Neligan 4th edition. Elsevier. 2016 Vol. 4;350-380.


The

3) William VP, Benjo AD. The National Cost of Hospital-Acquired Pressure Injuries in the US. Int Wound J. 2019;16(3):634-640.

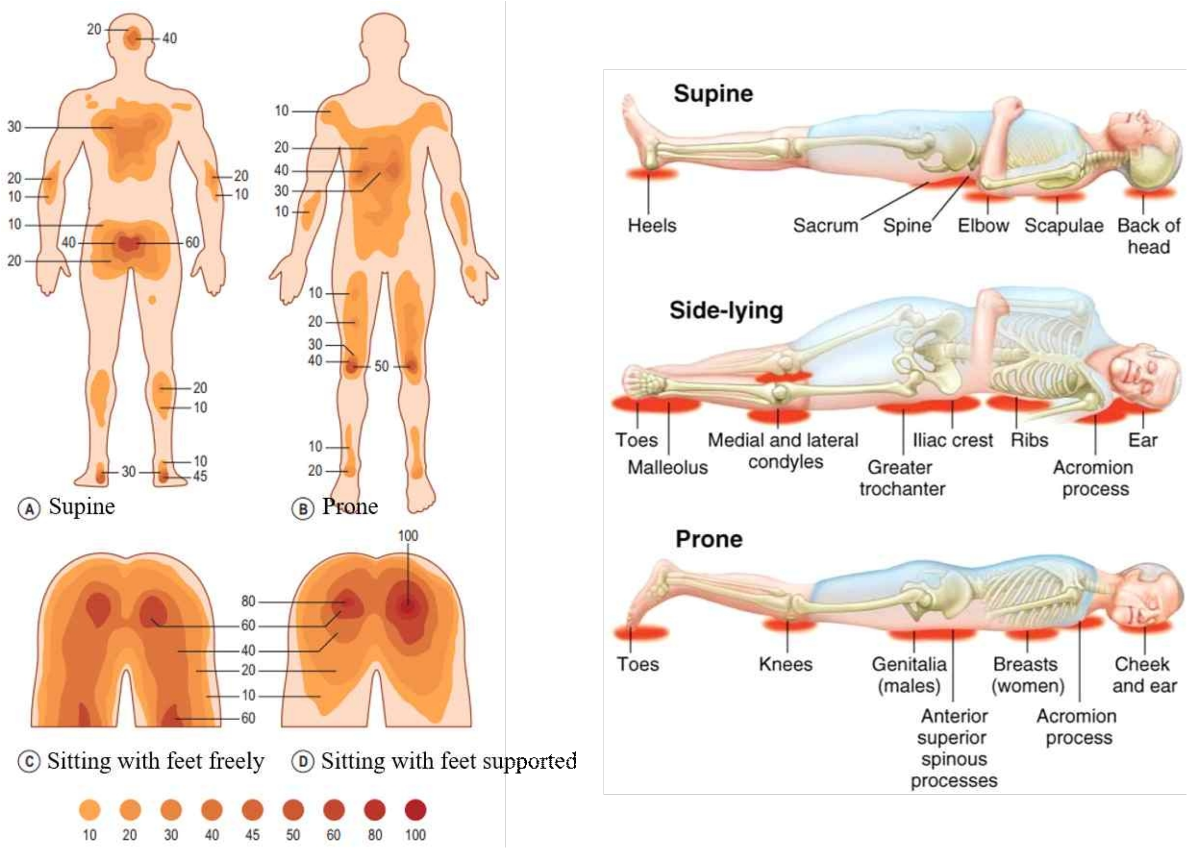


**Figure 2.** Soft tissue loading pressure and pressure ulcer predisposition based on posture.

Pressure ulcers can be categorized in a variety of ways, but the most commonly used is the staging system published by the National Pressure Ulcer Advisory Panel (NPUAP). The NPUAP classifies pressure ulcers into stages 1 through 4 based on depth, with 6 stages in total, including 2 unclassified ulcers.[1] **From NPUAP stage 2 onward, skin** damage is present, and at stage 3 and above, skin tissue necrosis occurs, requiring limbectomy.

Pressure ulcers are a classic example of a chronic wound with an ongoing inflammatory response, and while the length of **time the** missing tissue takes to **heal** depends on the extent and depth of the wound, it can range from **one to three months for mild** to
Degrees ⑨ and **above** require at **least 6 months to a year to recover**. Recovery, however, is not guaranteed and is dependent on the resolution of the direct cause of the pressure, as well as the management of the indirect causes that have not been addressed, such as internal medicine, nutrition, and ongoing wound care.

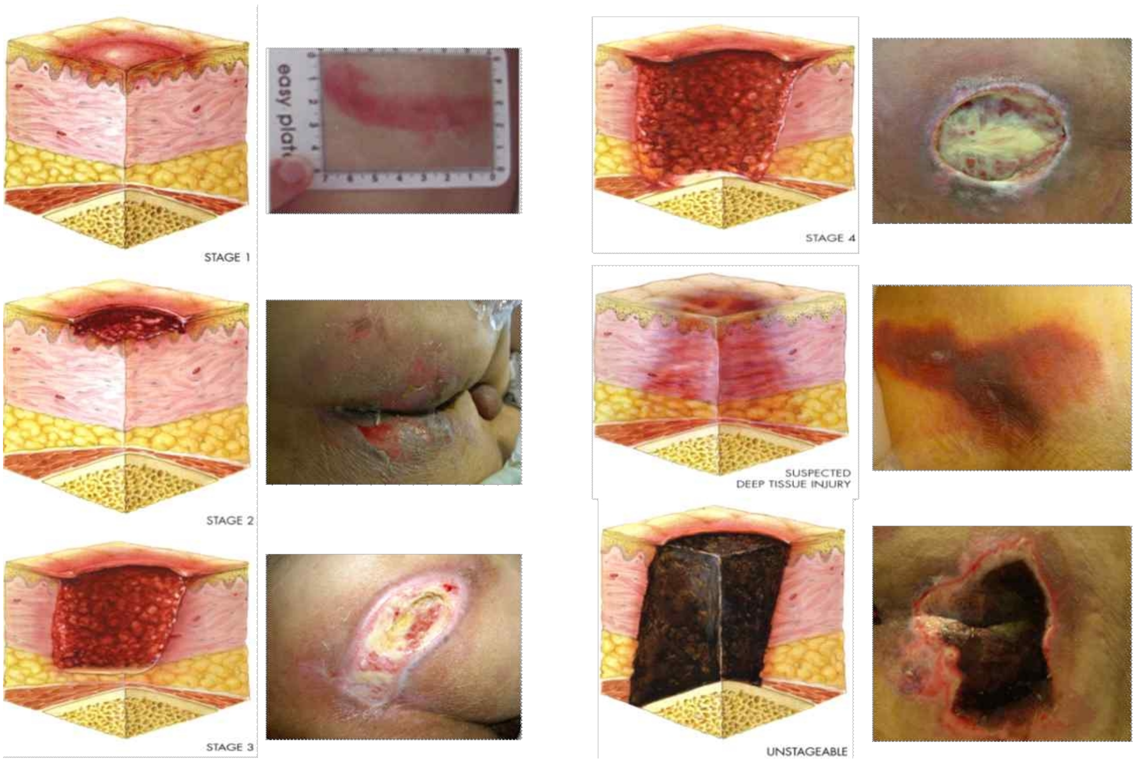


**Figure 3.** NPUAP Pressure Ulcer Classification

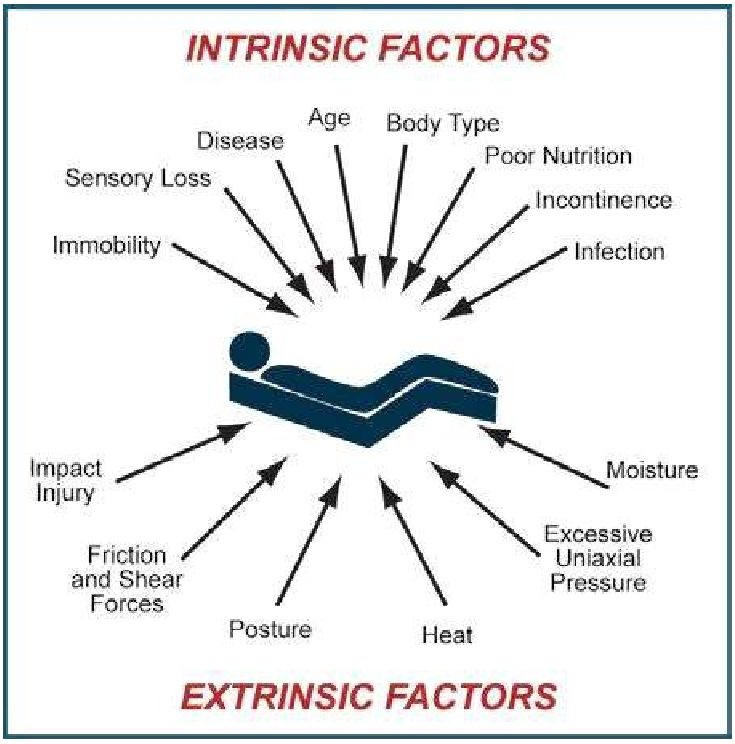


**Figure 4.** Multifactorial causes of pressure ulcers

This **multifactorial, high societal cost of pressure** ulcers is a **global issue, and** medical experts from the United States, Europe, and the Pacific Rim (National Pressure Injury Advisory Panel - European Pressure Ulcer Advisory Panel - Pan Pacific Pressure Injury Alliance) have published three international guidelines on pressure ulcers since 2009 (first edition 2009, revised 2014, 2019). The guidelines state that the **best treatment for pressure ulcers is prevention** and emphasize the **importance of position modification**. Depending on the study, up to 95% of pressure ulcers are preventable.[1]

However, once skin necrosis has occurred, it can be accompanied by infection and should be preceded by limbectomy. After limbectomy, the defect can be surgically repaired with flaps, but if general anesthesia cannot be performed, **conservative treatment** is the only option. In this case, it is **very important to control inflammation and accelerate the wound healing process**. To date, many wound coverings have been developed, and wound healing can be achieved by choosing the right antiseptic for the condition. However, each treatment has its own limitations and there is no single method for the conservative treatment of pressure ulcers.

**Photobiomodulation therapy, which involves irradiating the** affected area with low-powered light to induce healing, has also been reported to help heal wounds by promoting oxidative modulation and growth factors. It is also known to have anti-inflammatory, analgesic, and sterilizing effects, and there are even studies that have verified the effectiveness of photobiomodulation therapy for pressure ulcers.[4,5] However, the exact mechanism of photobiomodulation is still unclear.

A systematic review of five papers reported in 2020 by Francislene FCP et al,

**While the 658 nm wavelength produced meaningful results in pressure ulcer healing,** the 808 nm or
990 nm did not show a significant difference. Most studies used energy densities of 1 J/cm$^2$ or 4 J/cm$^2$ and treated for 4-6 weeks, 3-5 times per week. Treatment effects were analyzed by quantitative analysis of wound size and depth, degree of re-epithelialization, growth factor secretion, and cytokines. However, when the studies were analyzed for bias, very few were complete. In addition, only the final outcome was analyzed, not the healing process of chronic wounds.[5]

4) Chen C, How WH, Chan ESY, Yeh ML, Lo HLD. Phototherapy for Treating Pressure Ulcers. Cochrane Database Syst Rev. 2014;11(7):CD009224.

5) Francislene FCP, Jorge VCF, Hellen R, et al., Effect of Photobiomodulation on Reparing Pressure Ulcers in Adult and Elderly Patients: A systematic Review. Photochem Photobiol. 2020;96(1):191-199.

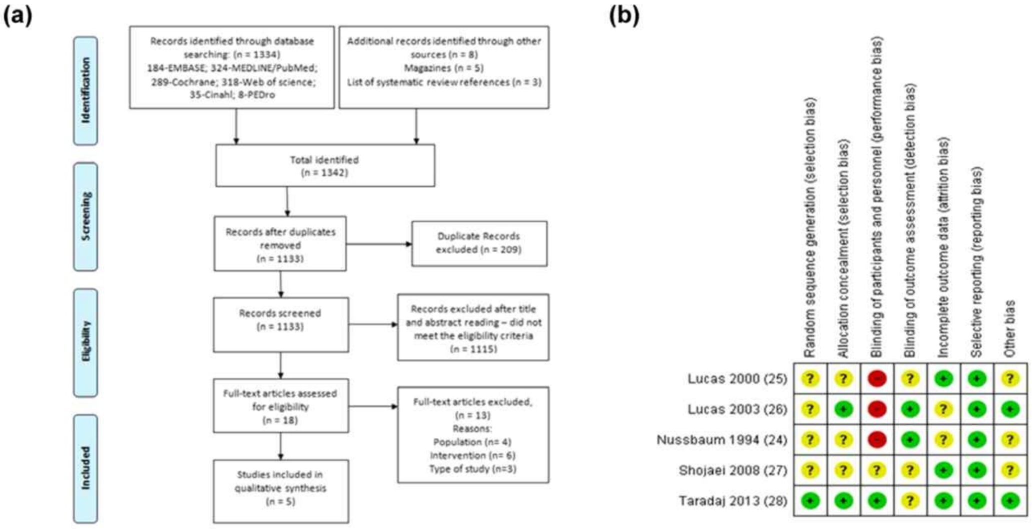


**Figure 5.** (a) Flowchart of study selection for systematic analysis. (b) Bias survey table of the five selected studies.

The **BELLALUX Lite, which will be** used in this clinical trial, was certified by IECEE-CB (IEC System for Conformity Testing and Certification of Electrical Equipment-Certified Body) in July 2019 and is a **class 2⑨ medical combination stimulator**. This product is a performance improvement and lightweight version of its predecessor, BELLALUX (model name: RED&AMBER-MD1), and emits four wavelengths: BLUE (460 nm) and NIR (850 nm) in addition to RED (630 nm) and AMBER (595 nm) wavelengths.

**A preclinical trial using rats** to verify the efficacy of 'BELLALUX', a systemic model, in treating pressure ulcers was **conducted at our headquarters in 2019**. The results showed that the Stimulation Index (SI) of the local lymph nodes was between 0.7 and 1.3 compared to the control group, which is lower than the SI of 1.6 for skin sensitizers, **proving safety**.

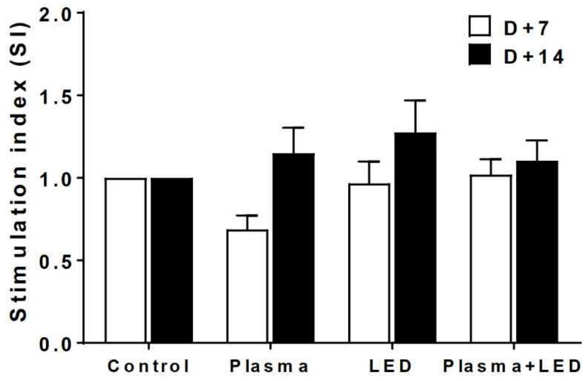


**Figure 6.** Preclinical test results for safety evaluation (Laboratory of

Photobiology, College of Medicine, Dankook University)

The **efficacy** evaluation was based on the recovery from infectious wounds, and it was found that the wound size was significantly reduced in the plasma and light groups compared to the control group, and the recovery was faster in the experimental group that was irradiated with both plasma and light.

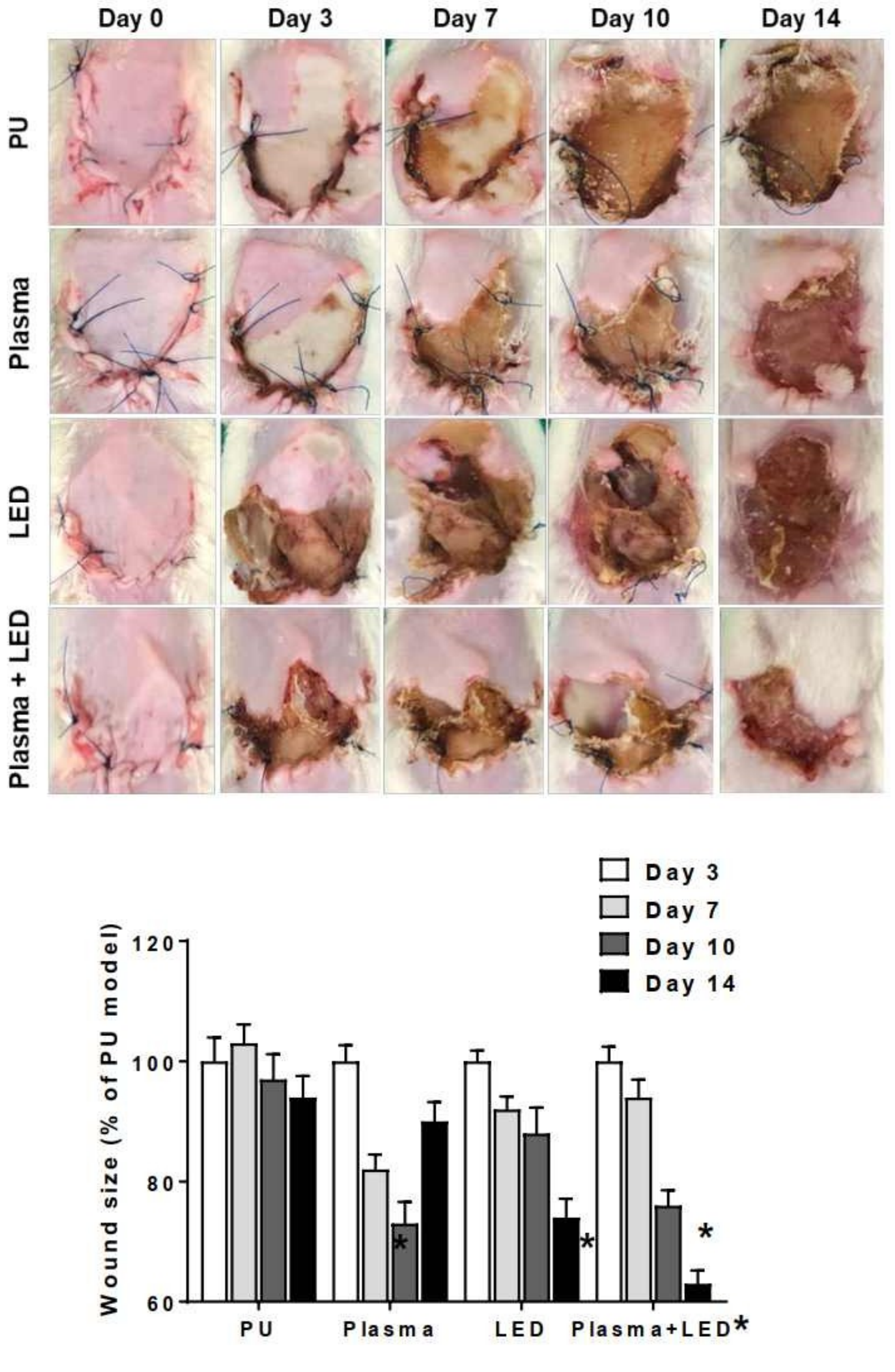


**Figure 7.** Preclinical test results for efficacy evaluation (Laboratory of Photobiology, College of Medicine, Dankook University)

Therefore, it is **necessary to evaluate the efficacy and safety of this medical device clinically, and this clinical trial is planned**. 생Although the plasma used in the preclinical trial was sterilized by ozone생 and was effective for infectious wounds, such as the one described above, it is not suitable for clinical trials due to the potential risk of ozone exposure to humans. This study **device (BELLALUX Lite), which is a successor to the model used in preclinical trials, also has a BLUE wavelength of 460 nm, which may provide enhanced sterilization**. The sterilizing power of the BLUE wavelength has been studied in our laboratory as follows.

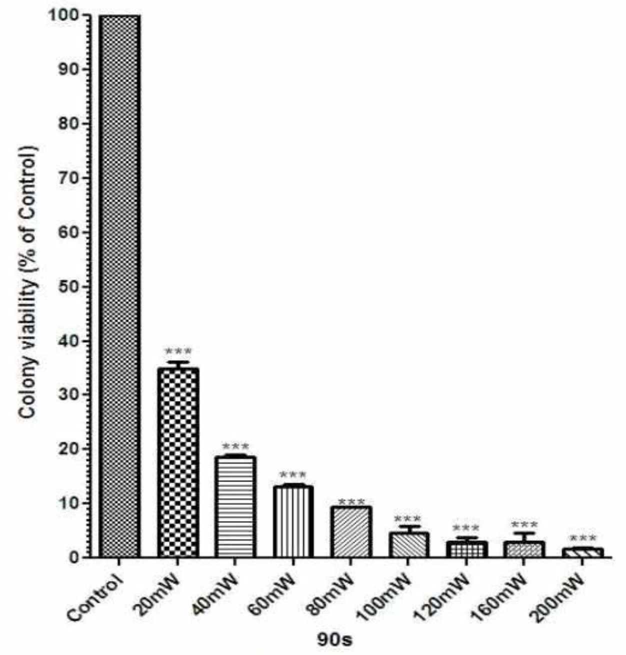


Fig. 6 90s, 20mW, 40mW, 60mW, 80mW, 100mW, 120mW, 160mW, 200mW로 조사한 *Propionibacterium acnes* 균의 viability

**Figure 8.**생 dependence of the anaerobic bacterium Propionibacterium acnes on the wavelength of 408 nm.

**Therefore, this device can be expected to have sufficient bactericidal power (BLUE wavelength) and wound healing power (RED+AMBER wavelength) for pressure ulcers, which are typical of chronic wounds, and clinical trials with this device are warranted.**

**In addition, this trial is minimal risk as the control group will receive standard conservative care (antimicrobial disinfection) and the experimental group will receive the additional benefit of the LED therapy device.**

○ **Overview of Investigational Medical Devices**

- **Product Overview**

- **Development History**

**Ltd.** developed the product by confirming that it is effective in treating pressure ulcers through preclinical trials as a class 2 therapeutic combination stimulation device that combines a low-power light irradiator and an infrared irradiator.

- **How it works**

This product is a class 2 medical combination stimulator that combines a low-power light irradiator and an infrared irradiator, and when electrical energy is applied, it emits energy in the form of light through a visible light LED lamp with a visible light [RED (630nm), AMBER (595nm), BLUE (460nm)] wavelength and a near-infrared lamp with a near-infrared (NIR, 850nm) wavelength, and this light is absorbed by cells and activates cell function, so it is a device developed for use in treating skin diseases and treating bedsores. This product is composed of an irradiation part and a main body part. The irradiation part is attached with a lamp and emits visible and infrared light when supplied with power, and functions to adjust the angle and direction of the irradiation part through the irradiation part connection, and the main body part supports the irradiation part and enables height adjustment of the product.

- **Shape and structure (appearance)**

- Appearance

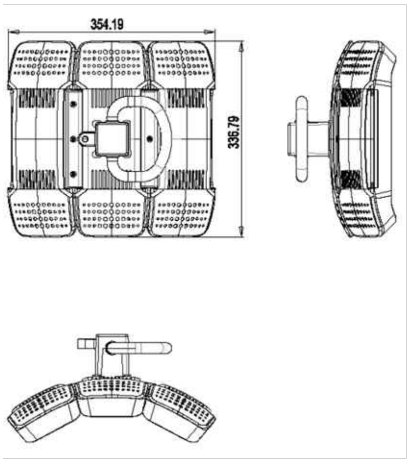


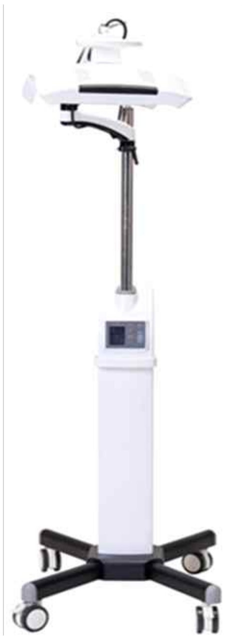

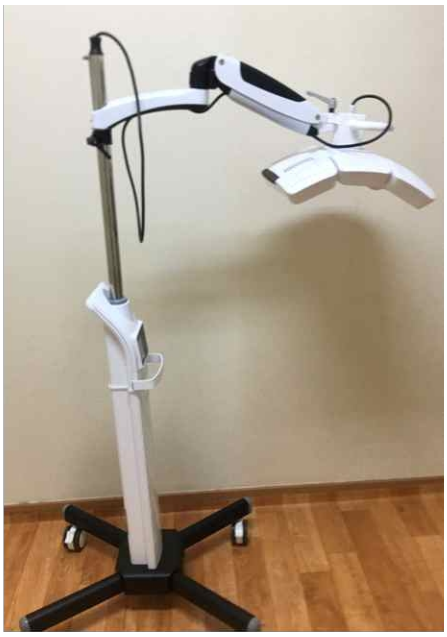

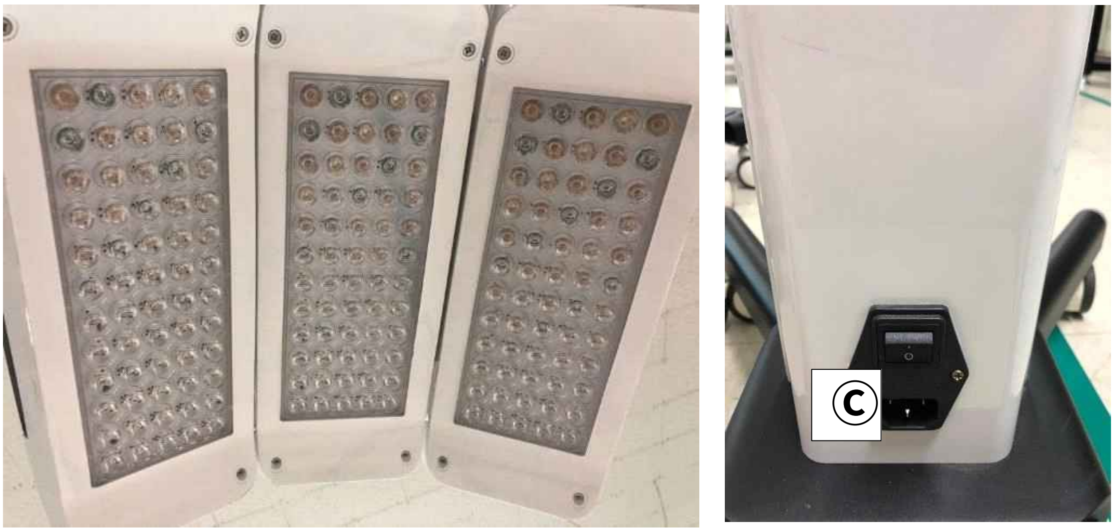


**Figure 9.** 'BELLALUX Lite' and product appearance

- **Appearance Description**

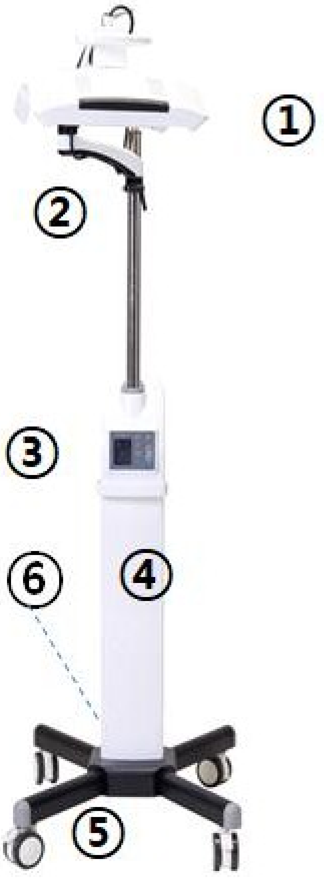


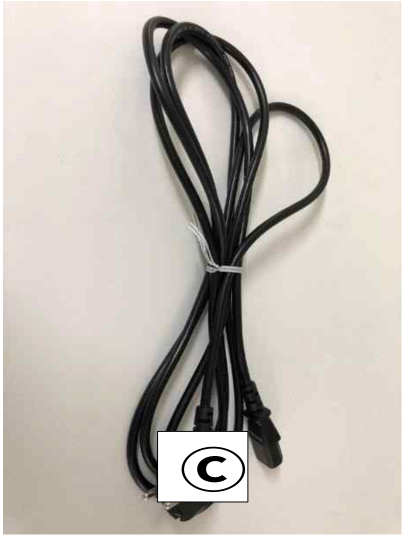

<table>
<tr><th>Num ber</th><th>Name</th><th>功能</th></tr>
<tr><td>1.</td><td>Investigations</td><td>Light Source - LED Light Output [Output in mW/cm at 15 cm (²)]
<table>
<tr><th>Wavelength/Number of LEDs</th><th>Step 1</th><th>Step 2</th><th>Step 3</th><th>Step 4</th><th>Step 5</th></tr>
<tr><td>Blue (460 nm) /45 pcs</td><td>0.80</td><td>2.20</td><td>3.60</td><td>5.10</td><td>8.40</td></tr>
<tr><td>Amber (595 nm) /45 pieces</td><td>2.00</td><td>4.50</td><td>7.00</td><td>9.00</td><td>12</td></tr>
<tr><td>RED (630nm) / 45 pcs</td><td>3.60</td><td>8.80</td><td>13.80</td><td>19.00</td><td>30.00</td></tr>
<tr><td>NIR (850 nm) / 45</td><td>5.60</td><td>12.40</td><td>21.00</td><td>28.00</td><td>40.00</td></tr>
</table></td></tr>
<tr><td>2.</td><td>Adjustments</td><td>Adjust the height and orientation of the light source</td></tr>
<tr><td>γ</td><td>LCD Screen & Buttons</td><td>LCD screen and operation buttons</td></tr>
<tr><td>④]</td><td>Body</td><td>The body of the product</td></tr>
<tr><td>⑤And</td><td>Movable Pedestal</td><td>Move the device while supporting the base</td></tr>
<tr><td>6</td><td>Power</td><td>Product power</td></tr>
<tr><td>⑦â</td><td>Power cord</td><td>Product power cord</td></tr>
</table>

- **Button Description**

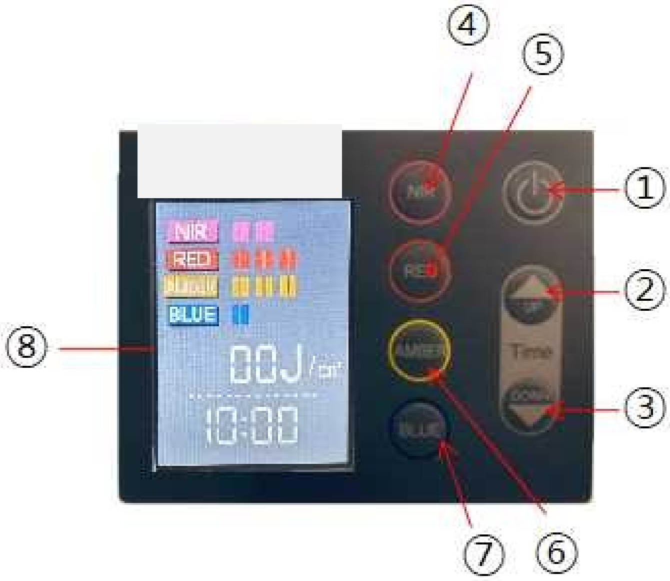

- External Actuation Button Descriptions

| Number | Name | 功能 |
|---|---|---|
| 1. | Operation buttons | Device On/Off switch |
| 2. | Time adjustment up button | Can be incremented in 5 minute increments up to 25 minutes |
| γ | Time adjustment down button | Can be lowered in 5 minute increments up to a minimum of 5 minutes |

| | | |
|---|---|---|
| ④] | NIR | Switch to adjust the NIR wavelength Can be set from 0 to 5 levels |
| ⑤And | RED | Switch to adjust RED wavelength from 0 to 5 steps |
| 6 | AMBER | Switch to adjust AMBER wavelength from 0 to 5 steps |
| ⑦â | BLUE | Switch to adjust BLUE wavelength from 0 to 5 steps |
| ⑧â | LCD Screen | LCD screen Displays the current status |

- **Shape and Structure (Attributes)**
- **How it works**

This product is used to irradiate visible light and infrared light energy from LED (Light Emitting Diode) to the affected areas of skin diseases and pressure ulcers of the human body by applying electrical energy through the product power supply to the lamp made using LED (Light Emitting Diode).

- **Electrical ratings**

- Rated voltage : 220V AC
- Rated frequency : 60Hz
- Power consumption: 200W

- **Classification by type and degree of protection against electrical shock**

- Class 1 device, no mounting

- **Safeguards**

- Fuse(2A) : Automatically cuts off the power to the device when the power is short-circuited or there is an abnormality in the device.

- **Embedded software**

- Model Name: Bellalux Lite FW
- Name: Lite Light Source Control Program
- Software safety rating: A

- Medical Device Cybersecurity Safety Rating: Medium
- Algorithms, Structure, and Key Features

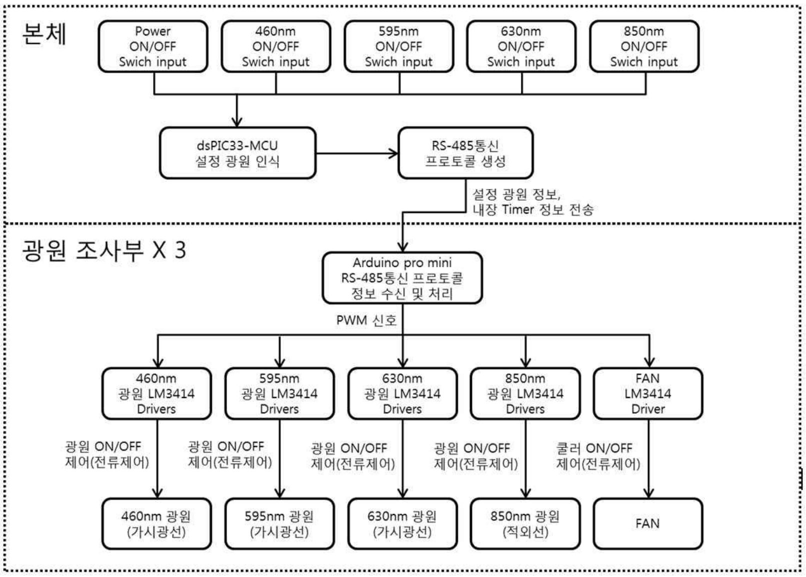


**Figure 10.** Schematic of the 'BELLALUX Lite' algorithm

- **Performance: Radiant Power (15 cm from the irradiator)** [Output i n mW/cm$^2$ )]

| Wavelength | Step 1 | Step 2 | Step 3 | Step 4 | Step 5 |
|---|---|---|---|---|---|
| **BLue** | 0.80 | 2.20 | 3.60 | 5.10 | 8.40 |
| **Amber** | 2.00 | 4.50 | 7.00 | 9.00 | 12 |
| **RED** | 3.60 | 8.80 | 13.80 | 19.00 | 30.00 |
| **NIR** | 5.60 | 12.40 | 21.00 | 28.00 | 40.00 |

* It must be within ±20% of each criterion.

- **Output time**: up to 25 minutes, adjustable in 5 minute increments
- **Light wavelength** : BLUE(460nm±10nm), AMBER(595nm±10nm), RED(630nm±10nm), NIR(850nm±10nm)
- **Irradiation area**: 15cm away from the LED light source
- **Safety device**: blown fuse cuts off power in case of overcurrent

- **Control Device (Sham Device)**

The control device has the same geometry as the investigational product BELLALUX Lite and has less than 10% of the light output (LEDs in the irradiated portion of the control device are 12 mW/cm$^2$ or less). Manufactured by Link Optics, Inc. to resemble the irradiated portion of the study device. In

addition, other appearance and operation methods will be the same as the clinical trial device.

## 2. Research Objectives

This is a **single-center, double-blind, randomized, parallel-group, prospective** **exploratory clinical** trial to evaluate the safety and efficacy of the **medical light irradiator BELLALUX Lite** on wound healing in patients with **mild pressure ulcers**.

- **Primary Objective**: The primary objective of this study is **to evaluate** the **efficacy of BELLALUX Lite** in the treatment of mild pressure ulcers by comparing wound size, degree of re-epithelialization, and immunochemical markers 4 weeks after application of the investigational device.

- **Secondary objective**: **To evalu**ate the **safety of BELLALUX Lite** as assessed by investigator-assessed medical examinations and adverse events 4 weeks after application compared to before application of the investigational device.

## 3. Name and address of the organization conducting the study

## 4. Research organizations

This clinical trial was selected for the 2020 First Interdepartmental Medical Device Research and Development Project (Clinical Support by Product Development Stage).

## 5. Full name and title of principal investigator, co-investigator, and contact person

**(1) Full name and title of the principal investigator and co-investigators.**

**(2) Full name and title of the administrator managing the investigational medical device**

## 6. Study Period

The duration of the study will be 30 **months from the date of Institutional Review Board (IRB) protocol approval**. The estimated duration of each phase is as follows

**(1) Report IRB clearance**: 2 months

**(2) Subject recruitment period**: 13 months

Patients with stage 2 pressure ulcers who meet the inclusion criteria currently see an average of 10 new patients per month at our center for foot 생 . Of these, an average of 5 patients per month meet the exclusion criteria. Therefore, if we exclude those who do not consent to the study**, we expect an average of 3 study participants per month**. We estimate that it will take 13 months to recruit 39 subjects.

**(3) Subject study duration**: 12 months

Pressure ulcers are chronic wounds that take a long time to fully heal when treated conservatively. **Mild wounds** require **1-3 months** and **moderate to severe wounds** require **at least 6 months to 1 year**. There is also a **wound maturation period up to 6 months after healing, so follow-up is required 6 months after healing**.

However, healing of the wound is not always achieved, and is dependent on the management of the indirect causes of the wound, such as internal medicine and nutrition, and ongoing wound care, that have not been addressed in addition to the relief of the direct causes of pressure, leading to sustained pressure. **Sufficient clinical time may be required for** this.

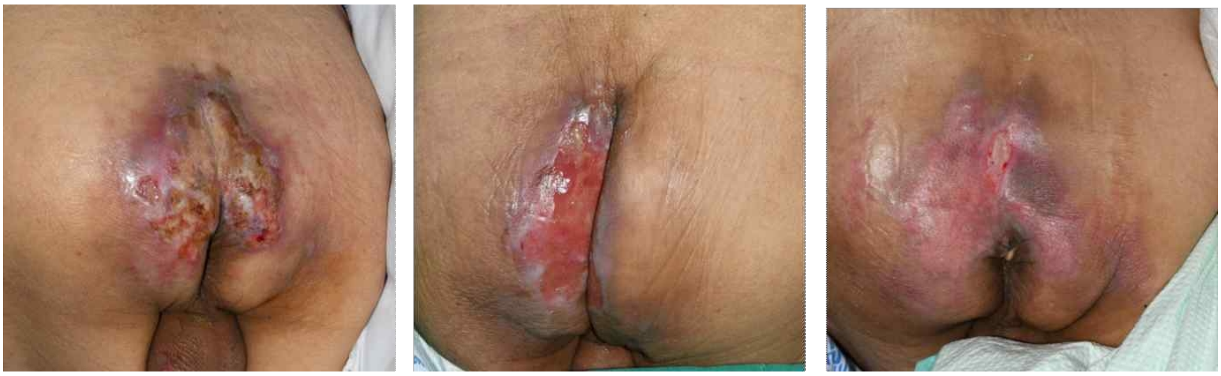

**Figure 9.** A stage 2 pressure ulcer in the hip area that healed over 3 months with conservative treatment.

(4) **Clinical outcome analysis (statistical processing period) and report generation**: 3 months + 9 months (variable)

생However, if a situation arises that may affect the progress of the clinical trial, the timeframe may be changed to the

Possibly.

| | 2020 | | 2021 | | | | 2022 | | | | 2023 | | |
|---|---|---|---|---|---|---|---|---|---|---|---|---|---|
| | 3/4 | 4/4 | 1/4 | 2/4 | 3/4 | 4/4 | 1/4 | 2/4 | 3/4 | 4/4 | 1/4 | 2/4 | 3/4 |
| IRB approval | | | | | | | | | | | | | |
| Recruiting subjects | | | | | | | | | | | | | |
| Clinical trials | | | | | | | | | | | | | |
| Analyze results and Build reports | | | | | | | | | | | | | |

**Figure 10. A** schematic of this study

## 7. Research subjects

### [1] Selection criteria for subjects

(1) Patients with stage 2 pressure ulcers (based on NPUAP guidelines) in the hip area.

(2) Adults 19 years of age or older

(3) People who have no problem expressing themselves

### [2] Exclusion criteria for subjects

(1) Pregnant and nursing women

(2) Prior hip surgery

(3) Recurrent pressure ulcers

(4) People with osteitis

(5) Isolated with resistant strains

(6) Unable to be in the supine position for more than 30 minutes

(7) People who need to take immunosuppressants or steroids for a medical condition.

(8) Have a light sensitivity (such as photosensitivity) or are taking medications related to light.

(9) Other cases where, in the opinion of the investigator, the study cannot be conducted properly.

The

## 8. Estimated number of subjects and rationale

### (1) Number of subjects

Aiming for **16 subjects per** endpoint group, with a 15% dropout rate, **19 subjects per control and experimental group,** for a **total of 38 subjects.**

### (2) Rationale

This study is an **exploratory trial** and is intended to **provide a statistical basis for a confirmatory trial**. A 2020 systematic review by Francislene FCP et al. on the effectiveness of photopheresis생 in the treatment of pressure ulcers found that the five reviewed trials had a mean of 15.8 patients per arm, ranging from 6 to 40 patients per arm. We chose 16 subjects per arm as the minimum standard for this trial, with a predicted dropout rate of 15%. We will **target 19 subjects per group for the final evaluation**.

**The dropout rate was selected based on the fact** that **in** the **last two years, a** single faculty member (Clinical Research Director
) treated 250 patients with 45 deaths during the study period, an **18% mortality rate. Given the** relatively high mortality rate in patients with moderate to severe pressure ulcers, we predicted a **lower mortality rate** for **the subjects in this study and** set a dropout rate of 15%.

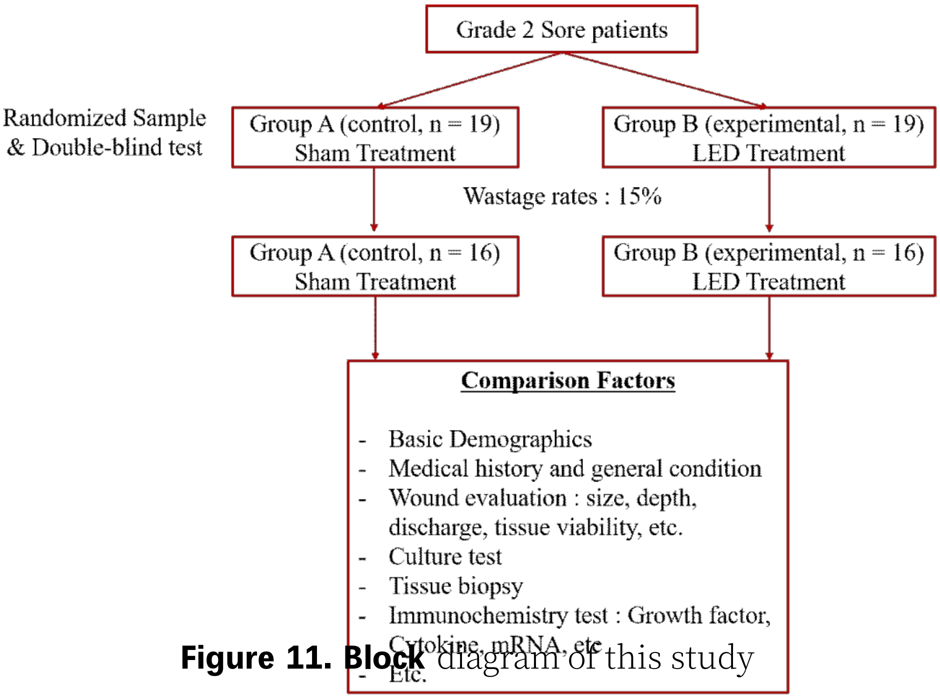


**Figure 11. Block** diagram of this study

## 9. Recruit subjects

Among the outpatients and inpatients referred to the plastic surgery department for pressure ulcer treatment, the inclusion and exclusion criteria were applied, and patients who were eligible for the study were explained about the study and gave their consent were included in the study.

## 10. Human subjects consent

Attach a separate informed consent form

## 11. Research Methods

- **Clinical trial methods**

This study was conducted on subjects who signed the informed consent form of their own volition, and after conducting the necessary examinations and tests according to the protocol, reviewed the selection criteria and exclusion criteria, and determined that they were suitable for this study, they were instructed on the study schedule and methods. Irradiate the subject's hip area pressure ulcer 15 cm away from the ulcer for a maximum time (25 minutes) with four wavelengths of LEDs in five stages of maximum power [stage 1 (12 mW/cm$^2$ ), stage 2 (27.9 mW/cm$^2$ ), stage 3 (44.6 mW/cm$^2$ ), stage 4 (61 mW/cm$^2$ ), and stage 5 (90 mW/cm$^2$ )]. LED irradiation will be performed 3 times per week, and after one week of treatment, a medical examination and wound biopsy will be performed. This will be done for a total of 4 weeks.

Efficacy will be assessed by determining wound size and degree of re-epithelialization as primary measures and immunochemical markers on biopsy as secondary measures. To assess safety, the study will include a physical examination and vital signs, as well as an adverse event investigation. Patients will be treated as usual for the conservative treatment of pressure ulcers with anti-생 agents and wound disinfection, with the addition of light therapy. The light will be a combination of four wavelengths and is predicted to be beneficial for bactericidal (BLUE wavelength) and tissue regeneration생 (AMBER and RED wavelengths).

- **Information about the devices used in the trial and the likelihood of being randomized to a treatment or control group.**

This medical device product is a class 2 medical combination stimulator, which is a light emitting diode (LED) device that combines a low power light

irradiator (energy density of 20J/$cm^2$ or output light less than 2W/$cm^2$ ) and an infrared irradiator. The irradiation part is approximately 336 × 354 × 105 mm in size, and is designed to be easily applied to the desired location by adjusting the angle and direction at the head connection part, and the height of the product at the main body part.

This is a double-blind, randomized, parallel group study with a control device (Sham device).

Prospective exploratory clinical trial with a design in which, in addition to the device, an identical-looking control device is applied to a control group of patients. The light irradiation of the control device is made to look similar to the irradiation of the investigational device using LEDs of 12 $mW/cm^2$ or less. Upon patient consent, patients will be randomly assigned to either the study or control device with a 50% probability.

- **Randomization**

Subjects who meet the inclusion and exclusion criteria and agree to participate in the study will be assigned a subject identification code once they are finally selected. The order of assignment to the treatment and control groups according to the subject identification number will be determined by the block randomization method using the statistical program "R" to assign randomization numbers of sufficient size to account for the predetermined block size. Once the randomization table is completed, it is independently managed by a third party independent randomization manager.

- **Double Blindness (BLinding)**

1) Blinding subjects: subjects, investigators

2) Blind Method

- To maintain blinding, blinding numbers are managed by a third party independent randomization administrator, and investigational medical devices are repackaged, labeled with blinding numbers, and shipped to the study site as directed by the independent randomization administrator.
- Subject blinding: The test and control devices are identical in appearance so that subjects do not know which group they are in, and the medical device manager does not tell subjects which device they have been assigned.
- Investigator blinding method: Investigational medical devices are labeled with a blinding number and sent to the clinical site under the direction of a third party independent randomization administrator, who verifies the labeling to ensure that subjects use the assigned device and does not inform the investigator.

3) Unblind

- Two sets of emergency code breakers are provided for the blinded trial, one set retained by the clinical trial site and one set sent to the medical device manager.
- The medical device administrator should place each subject's blind code in a separate envelope in the

Sutured and delivered to the Principal Investigator.

- The blinded unblinding table is enclosed in a cover sheet that can be easily peeled off and removed. In the event of an emergency, the cover can be removed to identify the medical device being used on the subject.

- The cover sheet should only be removed in an emergency. If the cover is removed by the principal investigator, the principal investigator must document the date and time of the removal of the blinding code and the specific reason(s) for doing so in the "Closure" section of the case record.

- In addition, the Principal Investigator must immediately notify the monitor and the granting agency of this unblinding.

- **Clinical trial timeline**

| Visit | スク Lining | Therapist | | | | Monitoring | |
|---|---|---|---|---|---|---|---|
| | Visit 1 | Visit 2-4 (Week 1) | Visit 5-7 (Week 2) | Visit 8-10 (Week 3) | Visit 11-13 (Week 4) | Visit 14 | Visit 15 |
| Viewpoint (±2 days) | D0 | D1,D3,D5 | D8,D10,D12 | D15,D17,D19 | D22,D24,D26 | D29 | D210 |
| Clinical trial description | ○ | | | | | | |
| Obtaining subject consent and screening Assigning a ning number | ○ | | | | | | |
| Conformance determination (line Inclusion/Exclusion Criteria) | ○ | | | | | | |
| Demographics | ○ | | | | | | |
| Medical history (past medical history, surgical history, Drug Dosing) | ○ | | | | | | |
| Pregnancy test (Fertility for women) | ○ | | | | | | |
| Medical exams and Vital signs | ○ | ○ | ○ | ○ | ○ | ○ | ○ |
| Annulus and Braden scaLe Measurement | ○ | ○ | ○ | ○ | ○ | ○ | ○ |
| Laboratory tests1 (blood tests, urine Check) | ○ | | | | | ○ | ○ |
| Laboratory tests2 (pelvic X-ray f) | ○ | | | | | | |
| Medical Device | | ○ | ○ | ○ | ○ | | |

| Applications | | | | | | | |
|---|---|---|---|---|---|---|---|
| Laboratory tests3 (Fungal culture tests, Organization⑨Sword) | | ○ | | | | ○ | ○ |
| Selection Criteria/Excluders Quasi-reconfirmation | | ○ | | | | | |
| Eligibility Determination and Subject⑨Record Number Grant | | ○ | | | | | |
| Investigating adverse events | | | | | | ○ | ○ |

- **Observational Test Method**

- **Screening Visit (Visit 1, D0)**: Subjects selected to participate in this study will receive an explanation of the study and be assessed in the following order.

1. Explain the test process and obtain written consent from subjects before allowing them to participate in the test.
2. Subjects are corrected on their screening numbers in order.
3. Research the subject's demographics and medical history.

④ Pregnancy test for women of childbearing age.

5. Performed a medical exam and vital signs.

6 Measures annulus and Braden scale.

⑦. A blood test, urinalysis, and pelvic X-ray are performed.

- **Visit 2 (D1 ± 2 days)**

1. After rechecking the selection and exclusion criteria before irradiation, the suitability is judged and the subject number is assigned.
2. Performed a medical examination and assessment of the affected area.
3. Performed bacterial identification and histologic examination.

④ Phototherapy performed.

- **Visit 3 (D3 ± 2 days) to Visit 13 (D26 ± 2 days)**

1. Performed a medical examination and assessment of the affected area.
2. Performed phototherapy.

- **Visit 14 (D29 ± 2 days)**

1. Performed a medical examination and assessment of the affected area.
2. Blood tests, urinalysis, bacterial identification, and biopsy.

3. Checked for adverse events immediately after the phototherapy machine.

- **Visit 15 (D210 ± 2 days)**

1. Performed a medical examination and assessment of the affected area.
2. Blood tests, urinalysis, bacterial identification, and biopsy.
3. Checking for adverse events during follow-up.

## 12. Observations

- **Observations and clinical test items**

- **Patient consent, screening number assignment, and demographic** survey: Before entering the study, the purpose and content of the study will be explained in detail to the subject, written consent will be obtained, a screening number will be assigned in the order of receipt of written consent, and demographic information will be collected. Records will include whether or not written consent was given and the date of consent, subject identification, gender, 생date, address, and contact information.
- **Determine eligibility and assign a record number**: If all items in the subject selection criteria are 'Yes' and all items in the subject exclusion criteria are 'No', the subject is determined to be eligible and assigned a subject identification code.
- **Medical history**: A detailed investigation and recording of the subject's medical history through a questionnaire and check of past medical records at the screening visit. This includes past history of diabetes, high blood pressure, surgery, medications, etc.
- **Medical examination**: A medical examination is performed at each visit, and any significant findings on the examination are recorded in the Medical Examination section of the case record, and any significant medical findings that meet the definition of an adverse event after study initiation are recorded in the Adverse Event case record. However, undesirable medical events that occurred prior to study initiation should be recorded in addition to the current medical history section.
- **Vital signs**: Check the subject's vital signs at each visit, including

temperature, blood pressure (systolic, diastolic), and pulse rate.

- **Wound assessment**: At each treatment visit, the researcher will assess the pressure ulcer for size (width and depth), color, presence of necrotic tissue, discharge, odor, microvascular bleeding, and granulation tissue formation.

Judgment.

- **Braden ScaLe Measure**: An indicator of pressure ulcer risk, with six items (sensory perception, skin moisture, activity level, positioning, nutrition, friction, and shear) that are scored (1-4 points for each item, for a maximum of 24 points).

- **Other inspections**

  - **Pregnancy test**: A pregnancy test is performed on women of childbearing age at the screening visit to confirm that the subject meets the inclusion and exclusion criteria, and the pregnancy is recorded in the case record.

  - **Laboratory test 1**: A laboratory test is performed to confirm that the subject meets the inclusion and exclusion criteria, and the test items are as follows. If the test is performed within 2 weeks, it can be replaced.

    - ✓ **Blood tests**: CBC, ESR, CRP,생 chemistry tests, etc. are performed at the screening visit, and normal test values and clinical significance are recorded in the case record sheet.

    - ✓ **Urinalysis**: performed at the screening visit, with normal test values and clinical significance recorded in the case report.

  - **Laboratory test 2**: A pelvic X-ray will be performed to confirm the presence of osteitis in order to select subjects and confirm that they meet the exclusion criteria.

  - **Laboratory tests3** : Bacteriologic identification and histologic examination will be performed immediately before study treatment (D1), immediately after study treatment (D29), and at long-term follow-up after study treatment (D210) to observe the change process.

    - ✓ **Bacterial identification test**: A bacterial identification test is performed on a tissue sample. Test results take about 7 days.

    - ✓ **Histologic examination**: Determine the process of tissue changes in the wound through histologic examination and simultaneously perform immunochemical tests (IL-1, 4, 6, 10, 13, TNF-a, TGF-b, MMP-1, 2,

      TIMP, Colla1, 3, etc.) to understand the wound healing process.

- ✓ **This test is performed using a 3- to 4-mm punch biopsy and** should **only be** performed on **consenting individuals**.
- ✓ **This test is required for the treatment of pressure ulcers, which are invasive or chronic infectious wounds, where the test** could **delay recovery or cause harm to the patient.**

**to the patient**. The following example shows the wound healing process of a pressure ulcer patient who had a bacterial culture and histology performed, demonstrating that the test was not harmful to the patient's wound healing.

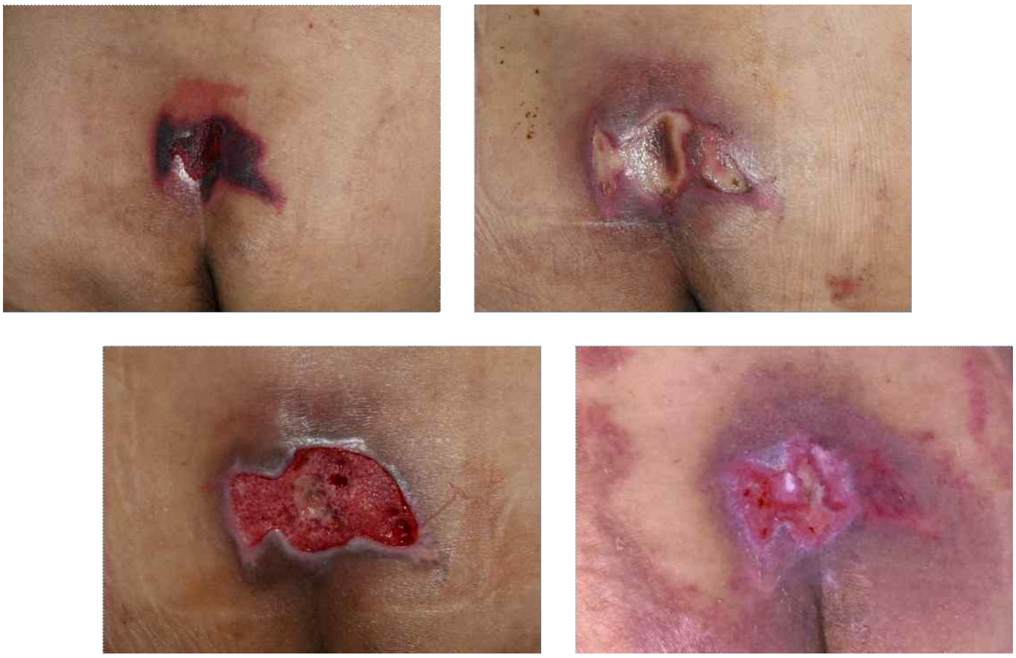

**Figure 12.** Example of spontaneous wound healing in a pressure ulcer patient with bacterial culture and histology.

- **Confirmation of adverse reactions**: Information on adverse reactions will be provided to subjects from time to time to educate them to report voluntarily, and the investigator will confirm the occurrence of adverse reactions through questioning. In the event of an adverse event, the date of onset and disappearance, the extent and consequences of the adverse event, the measures taken in relation to the irradiation and the causal relationship with the irradiation, the name of the drug or treatment other than the irradiation, and whether and how the adverse event was treated are recorded in detail on the case record sheet.

- **Expected adverse events in this study (**based on a 2014 Cochrane review by Chen C. et al)

  - ■ Mild: mild pain, mild redness, etc.
  - ■ Moderate: moderate pain, moderate redness, mild exudate, etc.
  - ■ Severe: bacterial growth, wound bleeding, tissue necrosis, etc.

## 13. **Criteria and methods for evaluating effectiveness** (including data analysis and statistical methods)

- **Primary efficacy measures**: **wound size and degree of re-epithelialization**
  - Wound size and degree of re-epithelialization in the control and experimental groups at 0 and 4 weeks were plotted against time, respectively, and differences between the two groups were analyzed using a paired t-test.
  - Pressure ulcer size and degree of re-epithelialization will be measured by the investigator on the day of the study visit by physical examination (width × length × depth: measured in cm using a ruler).
- **Secondary validity measure**: **immunochemical markers on tissue examination**
  - Pro-inflammatory cytokine (IL-1, 6) / Anti-inflammatory cytokine (IL-4, 10, 13) ratio was obtained and the progression to the proliferative phase was analyzed by comparing the control and experimental groups after 0 and 4 weeks.
  - Immunochemical markers are measured in the lab by researchers through RNA sequencing.

## 14. Safety evaluation criteria and methods

- **Evaluation Criteria**
  - A subject's complaint of an adverse event in the foot생 during or after use of the medical device.

    Whether you have symptoms of irritation in the hip area (stinging, hives, inflammation, itching, etc.)
  - Medical examination: presence of skin lesions (erythema, edema, etc.) in the hip area
  - Follow-up period: Follow-up will be conducted until 6 months after the end of the study.
  - Mild: does not interfere with the subject's normal daily생 routine, causes minimal discomfort, and is easily tolerated by the subject; the treatment is testimonial and does not affect the validity of the medical device with a full recovery; the degree of medical findings and the causal relationship to the clinical medical device are evaluated.
  - Moderate: If the condition causes discomfort that significantly

interferes with the subject's normal daily생 activities, is reversible with continued treatment, and does not affect the validity of the investigational medical device, the extent of the medical condition and its causal relationship to the investigational medical device will be evaluated.

- Severe: If it prevents the subject from performing normal daily생 activities, it is not treatable, and the investigational clinical device affects efficacy, the medical opinion will be evaluated for causality with the investigational clinical device.

- **Evaluation Methods**
  - Perform analyses on adverse events reported by subjects or identified by secondary review to assess severity and type of adverse event and the rate of occurrence of adverse events in the study and control groups .생

- **Reporting adverse events**

1) The severity of the adverse event: mild, moderate, severe, etc. can be labeled in the adverse event report card.
2) Causality with an investigational medical device: Determine causality based on the evaluation criteria so that it can be marked on the adverse event report card.
3) Disposition: Allows you to display in the record table when there is a change in the clinical trial method, such as changing the method of use, reducing the frequency of use, or discontinuing use due to an adverse event.
4) Treatment course: Allows you to describe the disappearance or worsening of an adverse event due to treatment after the event occurs.
5) Investigator's view: Allows you to record the investigator's view of an adverse event.

- **Reporting method**: This information is reported in accordance with the regulations on medical device clinical trials and the standard work instructions prescribed by the medical device clinical trial center.

1) 생An adverse event is any unintended symptom, manifestation, or disease that occurs in a subject during a clinical trial, and should be recorded in the case record based on the medical findings and severity of the adverse event and an assessment of the causal relationship to the investigational medical device. Therefore, the evaluation criteria for the causal relationship of the adverse event to the investigational medical device should be presented.
2) Safety criteria refers to the criteria for evaluating the severity of predicted adverse events and adverse events as they occur생 and should be recorded in the case record.

3) The safety evaluation method provides statistical analysis methods and evaluation criteria for the comparative evaluation of the test group and the control group on the frequency of adverse events, adverse medical device events, and adverse events associated with the investigational medical device.

4) Adverse Event Reporting: The Principal Investigator must report all serious adverse events to the Sponsor immediately.

and make a further report with documented details in accordance with the plan. In the case of an adverse event that results in death or threatens 생 persons, a further report must be made within 7 days of the date the sponsor is informed or becomes aware of the event, in which case detailed information about the adverse event must be provided within 8 days of the initial report. In the case of other serious and unexpected adverse medical events, the sponsor must report each to the head of the Ministry of Food and Drug Safety within 15 days of the date the sponsor receives the report or learns of it.

## 15. Predicted side effects and precautions and actions

- **Possible side effects**

- Side effects such as increased exudate, bacterial growth, wound bleeding, and 생tissue necrosis due to the promotion of an inflammatory response are 발⊠ possible.
- 생A 2014 Cochrane review by Chen C. et al. found that side effects such as pain, microbleedi생ng, and redness are also 발⊠ possible with photodynamic therapy.
- If you have an adverse event, record the name of the event and the severity of the symptoms in the case log.

- **Cautions for use**

- Do not shine the light directly into your eyes. Shining a beam of light into your own eyes, into the eyes of another person, through a magnifying glass, or looking at a reflection in a mirror can cause temporary impairment to your eyes, and prolonged exposure can be harmful to your eyes.
- People with light-sensitive constitutions or those taking related medications may be harmed by this product and should consult a professional before use.
- When using this product, you must follow the instructions in the user manual.

## 16. Stop and drop criteria

- **Stop by**
  - If during the course of a clinical trial, an adverse event, adverse reaction, etc. is observed and it is deemed unreasonable to continue the clinical trial, the principal investigator must request the IRB to stop the clinical trial, and the IRB's decision must be followed.

to stop the trial.

- If the sponsor wishes to stop the clinical trial for reasons such as the safety of the investigational medical device, the sponsor may request the IRB to stop the clinical trial and stop the clinical trial in accordance with the IRB's decision.

- **Elimination Criteria**

- Record whether all subjects in the study have completed the study, and if irradiation or observation has been stopped, record the reason. For subjects in an ongoing clinical trial, the study may be discontinued in the following cases
  - ■ Violation of inclusion and exclusion criteria
  - ■ Use of prohibited medications during the treatment period: steroids, immunosuppressants
  - ■ If a subject develops a serious adverse event생 or if the subject requests discontinuation of the study due to an adverse event, or if the investigator believes that the adverse event warrants discontinuation of the study.
  - ■ Subjects with systemic disease that was not detected by pre-study testing.
  - ■ The subject or the subject's legal representative requests discontinuation of the trial due to unsatisfactory treatment effect during the trial.
  - ■ Violation of the protocol by the investigator or subject.
  - ■ Subject withdraws consent to participate in a clinical trial
  - ■ If a subject can't be tracked
  - ■ If you have a problem with irradiating your subjects with light 생
  - ■ You have any other medical condition that requires immediate medical attention and, in the judgment of생 , you are not fit to proceed with the test.

- **Handling stops and dropouts**

- 생생If a subject withdraws or drops out during the study, this is documented in the case record and followed up with a questionnaire or telephone visit for possible adverse events.

- Dropouts will be included in the statistical processing of safety and efficacy assessments unless there is good reason or evidence to exclude them, and analyses will be conducted using the Last Observation Carrying Forward (LOCF) method, which replaces missing values with the value immediately prior to the dropout.

## 18. Research subject risks and benefits

For mild pressure ulcers, conservative treatment is performed, and repositioning to relieve pressure is an important part of the treatment, along with anti-생 agent therapy and wound disinfection. There are many wound disinfection products available, but each has its own limitations and there is no clinically established method of disinfection. If you decide not to participate in this study, you will receive usual conservative care, and you will be able to express your refusal of phototherapy even during the study, and you will be able to receive usual conservative care immediately thereafter. Therefore, we will strive for recovery of your condition regardless of whether you participate in the study, and we will try to minimize harm to you.

생The expected side effects of the medical device in this clinical trial may include irritation symptoms (itching, hives, inflammation, itching, etc.) in the buttock area, erythema and swelling in the buttock area, etc. 생In the event of any damage or harm related to this clinical trial, the sponsor ( Link Optics Co., Ltd.) will take full legal responsibility and compensate for the damage. However, other injury-related or disease-related costs such as lost wages will not be compensated.

The expected benefit of participating in this study is that the study will pay for blood tests, urine tests, radiology tests, biopsies, and bacterial identification tests related to the study while you are participating in the study. You will be responsible for any hospitalization and laboratory fees not related to the conduct of this study.

## 19. Human subject safety and privacy measures

- **Protocol for Victim Compensation (attached)**

생In the event of any damage or harm related to this clinical **trial, the sponsor (Link Optics, Inc.)** will be **solely responsible and will compensate for the damage,** and side effects and worsening of the disease will be treated with known treatment methods. However, **other injury-related or disease-related**

**expenses, such as lost wages, will not be compensated. The** study physician and research staff will provide you with additional information about all other possible financial compensation.

temporary pain or damage that can be easily treated, and the site determines that treatment is necessary (coverage is limited to the cost of the necessary treatment).

You need to be hospitalized or have your hospital stay extended.

Causes persistent or significant disfigurement or diminished donation.

④ Causes birth defects or abnormalities

Causes death or threatens생 people.

### - Post-trial care for subjects

Patients who drop out of the clinical trial or do not respond shall be instructed to receive other appropriate treatment, and patients who are terminated from the clinical trial shall follow the treatment procedures of the hospital for subsequent treatment, and the subsequent treatment costs shall be supported by the subject. However, in the event of an adverse event생 , after confirming the existence or absence of a causal relationship with the clinical medical device used in the clinical trial, if the adverse event생 is caused by the clinical medical device used in this clinical trial, the sponsor shall pay the treatment fee until the adverse event disappears.

### - Measures to protect the safety of human subjects

- **Clinical trial site**

The head of the organization conducting the clinical trial shall ensure that the clinical trial is conducted appropriately by equipping the clinical laboratory, facilities, and specialized personnel necessary for the conduct of the clinical trial and taking necessary measures in case of emergency.

- **Institutional Review Board (IRB)**

The IRB must be organized in accordance with national laws, regulations, and practices and must protect the rights, safety, and welfare of human subjects. In addition, if subjects from vulnerable environments are included in the trial, the validity of the reasons for doing so should be scrutinized. In fulfilling its duties, the IRB must take necessary action against the investigator, including ordering a halt to part or all of the trial, if it determines that informed consent h a s not been properly obtained, the trial is not being conducted in accordance with the protocol, or a serious adverse event or adverse reaction has occurred.

- **Investigator**

1) Investigator refers to the principal investigator, study staff, and study coordinator. An investigator is an individual who has agreed with the sponsor and has been approved by the Institutional Review Board and the Commissioner of Food and Drug Safety.

Comply with the protocol and conduct clinical trials.

2) 생During and after the clinical trial, the investigator shall ensure that the subject receives appropriate medical care for any adverse event that occurs in the clinical trial, including clinically significant abnormalities in laboratory tests, and shall inform the subject of any co-morbidities that come to the investigator's knowledge that require medical attention.

3) The investigator accurately analyzes and understands the study plan and actively responds to subject concerns.

- **Sponsor**

1) A person with responsibility for the planning, management, and financing of a clinical study, typically the manufacturer (including the importer) of a medical device in the case of a medical device clinical trial.

2) Ensure that clinical subjects, methods, and the form and content of case reports are conducted in accordance with the procedures in the protocol.

3) The sponsor's inspection plan and procedures should be based on the importance of the study, the number of subjects, the type and complexity of the study, the degree of potential risk to subjects, and any known problems in conducting the study.

- **Monitoring**

1) Monitoring refers to the activity of overseeing the progress of a clinical trial and reviewing and verifying that the trial is conducted and recorded in accordance with the protocol, Standard Operating Procedures, Good Clinical Practice, and applicable regulations.

2) Monitoring of clinical trials is accomplished through periodic visits and phone calls by clinical trial monitors to clinical trial sites. During these visits, the monitor will check original patient records, investigational medical device care records, and data storage (study files).

3) Trial monitors keep an eye on the progress of the study and discuss any problems with the investigator.

- **Changes to protocols**

1) After the protocol has been approved by the IRB and the Commissioner of Food and Drug Safety, any changes to the protocol due to broader or

higher risk procedures, changes in subject selection criteria, or additional safety information must be approved by the IRB and the Commissioner of Food and Drug Safety.

2) When revising the protocol, the date of revision, the reason for revision, and the contents of the revision must be recorded and kept.

3) The investigator must not conduct the clinical trial in a manner that deviates from the protocol unless the change is necessary to eliminate an immediate risk of harm to subjects생 and the IRB and the Commissioner of Food and Drug Safety approve the change. 생If a protocol change is made prior to IRB approval to eliminate an immediate risk to subjects, the change must be submitted to the IRB, the sponsor, and the Commissioner of Food and Drug Safety as soon as possible. And send the sponsor a document approved by the IRB chair or secretary.

4) Minor modifications or clarifications that do not impact the study do not require approval and are administrative changes.

- **Informed Consent**

1) Informed consent refers to the process by which a subject is provided with all information relevant to the study in an informed consent document before deciding whether or not to participate in the study, and confirms that he or she is voluntarily participating in the study by signing and dating the document.

2) If the subject or their representative is unable to read the consent form, subject manual, and other documented information, an impartial party should be present throughout the consent process.

3) Before obtaining consent, investigators must give subjects or their representatives sufficient time and opportunity to ask questions about the details of the study and to decide whether to participate in the thawing study, and must answer all study-related questions to the satisfaction of the subjects or their representatives.

- **Confidentiality of subject records**

1) That records that could identify subjects will be kept confidential, and that subjects' identities will remain confidential if the results of the trial are published.

2) The sponsor, monitors, and inspectors involved in this study may have access to the subject's records for the purpose of monitoring and inspecting the study and managing its progress. By signing this protocol, the subject acknowledges that the sponsor or monitors and inspectors

may review or copy the subject's charts and case notes to verify the subject's records in accordance with local laws and ethical considerations. Such

Information must be kept confidential.

3) All documents related to the trial, such as case notes, are recorded and identified with a subject identification code (usually the subject's initials) rather than the subject's name.

- **Retention of records**

Ensure that all materials and records related to the conduct of clinical trials are well preserved and secured. After completion of the clinical trial results report, clinical trial-related documents shall be preserved for 10 years from the end of the clinical trial.

- **Processing specimens**

- All specimens are coded and de-identified.
- Mycobacterial identification specimens: Performing tests and processing specimens in the Department of Diagnostic Laboratories
- Histology specimens: Specimens remaining after immunochemical testing at the Translational Clinical Medicine Center, Room 328 of the School of Medicine 발생 will be disposed of at the medical waste location in the City Laboratory.

**- What else you need to run a clinical trial safely and scientifically**

- **Case notes**

1) This study uses paper case notes to collect data. When we say subject's documentation, we mean the subject's records from their doctor that are kept at the site. Most of these are hospital or physician charts, and all information recorded in the subject's case notes must be consistent with these documents.

2) It is the responsibility of the Principal Investigator to record, review, and sign the case notes.

3) After completion of the case notes, the Principal Investigator will sign each case note to certify that the information recorded in the case note is true. This means that the Principal Investigator has final responsibility for the data related to the study in the case notes.

- **Monitoring**

1) The Sponsor or an organization entrusted by the Sponsor with monitoring duties will conduct monitoring to protect the rights and welfare of subjects and to verify the quality and reliability of the study data. The sponsor will select the monitor(s) and ensure that the monitor's

Have documentation of rostering and qualifications. Monitoring personnel must also have sufficient scientific or clinical knowledge of the investigational device, protocol, consent form, subject manual, and other written information provided to subjects, as well as the sponsor's standard work practices and applicable laws and regulations.

2) The monitor verifies that the clinical trial-related data is accurate, complete and verifiable when compared with the supporting documents, and that the clinical trial is conducted in accordance with the approved protocol and relevant regulations, including Article 24 of the Medical Device Enforcement Rules (Standards for Conducting Clinical Trials, etc.).

3) The principal investigator and study personnel shall make available to the monitor any source documentation, as defined in the Good Clinical Practice for Medical Devices, that would allow the monitor to verify the data in the case record (source documents: hospital or personal charts, lab result records, appointment notes, etc.

- **Recording and archiving materials**

1) Ensure that there is a designated and secure storage area for all materials and records related to the conduct of clinical trials.

2) After the completion of the outcome report, an archivist is appointed to retain the trial-related documents for three years from the end of the trial.

- **Submit and publish reports**

1) The principal investigator, in consultation with the sponsor or site, will write a report on the content of this study.

2) Publications resulting from clinical trials should specify the participation of the principal investigator and study staff, and agreements regarding authorship should be in place before the manuscript is written.

3) All summaries, manuscripts, or presentations related to the trial must be provided to and reviewed by the sponsor or site prior to publication or

presentation.

4) Delete all materials deemed confidential by the site. In this case, the results obtained in this study are not considered confidential.

- **Contracts**

  - The sponsor shall enter into a clinical trial contract with the director of the clinical trial center, which s h a l l include matters related to the financing of the clinical trial, delegation and division of duties, and obligations of the sponsor and the director of the clinical trial center.

## 20. References


1) NPIAP-EPUAP-PPPIA. Prevention and Treatment of Pressure Ulcers/Injuries: Clinical Practice Guideline. The International Guideline 2019

2) Robert K, Juan LR, Jeffery E. Pressure Sores. Neligan 4th edition. Elsevier. 2016 Vol. 4;350-380.

3) William VP, Benjo AD. The National Cost of Hospital-Acquired Pressure Injuries in the US. Int Wound J. 2019;16(3):634-640.4)

4) Chen C, How WH, Chan ESY, Yeh ML, Lo HLD. Phototherapy for Treating Pressure Ulcers. Cochrane Database Syst Rev. 2014;11(7):CD009224.

5) Francislene FCP, Jorge VCF, Hellen R, et al., Effect of Photobiomodulation on Reparing Pressure Ulcers in Adult and Elderly Patients: A systematic Review. Photochem Photobiol. 2020;96(1):191-199.


### [Attachment] Protocol for Victim Compensation

- **Reward requirements**

Subject compensation under this Compensation Agreement is subject to the following requirements

생(1) The injury must have been caused by the investigational clinical device.

The subject's condition has worsened as a result of the clinical trial.

③ The investigator has complied with all aspects o f t h e protocol approved by the Korea Food and Drug Administration.

④ The failure was not caused by the tester's manifest negligence or breach of duty.

The subject has complied with all instructions given by the principal investigator or study staff.

⑥ The subject would have taken steps to minimize the incidence생 of damages resulting from the bodily injury.

- **Reasons for exclusion**

Damage due to insufficient effectiveness or efficacy of an investigational clinical medical device

Damage to the foot생 caused by the subject's negligence.

If it hasn't been used on a given body part for a given amount of time.

If you have not sought counseling and medical attention at the time of: side effect symptoms 발

Failure to comply with subject precautions.

**< Note to subjects**

1) People with photosensitivity should not use it.

2) Medications and foods that increase photosensitivity should be avoided when combined as they may cause burn-like damage to the area where the device is used.

- Drugs known to cause photosensitivity : Quinolones (nalidixic acid, ofloxacin, sparfloxacin, etc.), tetracyclines (minocycline hydrochloride, doxycycline hydrochloride, etc.), sulfa drugs, griseofulvin, tarzepine, ciazepines (etazit, cyclobenzazit, fenfluthizide, etc.), porphyrins (NAPP, etc.), phenothiazines (prochlorperazine, chlorpromazine, etc.), methylene blue, etc.

- Foods that increase photosensitivity

: Foods containing furocoumarin (celery, lime, carrots, parsley, figs, mustard, etc.)

3) Not for use by pregnant or lactating women.

4) Do not expose the product to excessive humidity, overheating, or overcooling.

5) Do not use the product when it is wet or leaking water.

6) The product will overheat and should not be used continuously for more than 30 minutes.
7) If the light source is heavily contaminated, the efficiency of the product will be reduced, so use a dry towel to remove any contamination before use.
8) When the product is in operation, the patient should not look at the illuminated light source with their eyes.

9) It should not be used on open wounds.
10) To prevent electrical shock, store away from contact with magnets or energized objects.
11) You may not modify, disassemble, or repair the product in any way.
12) If you feel uncomfortable while using the product, stop using it.

- **Reward criteria**

If there is a pre-agreed compensation amount or measure between the parties for an anticipated medical device adverse event, we will compensate according to that standard.

In other cases, we will compensate you according to the compensation method agreed upon between the parties, taking into account the extent, nature, duration, and similar cases of physical damage.

③ If the parties do not agree on the preceding paragraph, compensation shall be provided in accordance with the court's judgment and confirmation of the decision.

- **Compensation Process**

Subjects who suffer bodily injury under this compensation protocol must first request necessary medical treatment from the principal investigator of the clinical trial or the clinical trial center.

Subjects whose physical injuries are not cured despite the actions of the principal investigator or the institution may request compensation from the sponsoring organization.

③ After receiving the above request for compensation, the sponsoring organization shall promptly complete the investigation on the eligibility and compensation criteria for compensation and notify the subject about it.

④ The subject shall notify the sponsoring organization of any objections to the above notification within five (5) business days from the date of receipt of the above notification.

⑤ If the Subject fails to notify us of any objection after receiving the notice in paragraph (3), the Parties understand that we have agreed to compensation in accordance with the above notice.

⑥ If the subject notifies an objection in accordance with the provisions of Paragraph (4), the sponsoring organization shall recommend multiple objective experts to the subject to judge whether the subject is eligible for the

above compensation and the compensation standard, and the subject shall nominate one recommendation within three (3) business days from the date of the above recommendation. (If the subject does not nominate, the sponsor shall choose at random.)

- **Coverage**

This Compensation Statement applies generally to subjects participating in all clinical trials sponsored by the Sponsoring Organization, to the extent that there are no other arrangements between the Sponsoring Organization and the subject.

Any agreement that a subject enters into with any other third party related to the clinical trial that is not approved by the Sponsor for compensation for the clinical trial is not enforceable against the Sponsor.

We take care to ensure that subjects do not suffer any harm as a result of this study, and we pledge to be responsible under the Victim Compensation Protocol if any problems are caused생 by this study.
