# Supplementary material for: The validity and safety of multispectral light emitting diode (LED) treatment on grade 2 pressure ulcer: Double-blinded, randomized controlled clinical trial
Source: PLoS One. 2024 Aug 23;19(8):e0305616. doi: 10.1371/journal.pone.0305616 (PMC11343461; doi:10.1371/journal.pone.0305616)
Supplement: S16 File — (PDF) [file pone.0305616.s024.pdf]

Medical Device Protocols

Neck

A single-center, double-blind, randomized, parallel-group, prospective exploratory study to evaluate the **safety and efficacy of the medical light irradiator BELLALUX Lite on wound healing** in patients with **mild pressure ulcer co-morbidities.**

Protocol No	:	DKUHPS01_PU
Version No	:	4.0
Version Date	:	April 1, 2021

Table of Contents

1. **Name and location of the clinical trial site** 1

2. **Full name and title of the principal investigator and co-investigators.** 1

3. **Full name and title of the administrator managing the investigational medical device.** 1

4. **The name and address of the person who wants to conduct the trial.** 1

5. **Purpose and background of the trial** 2

5.1 Purpose 2

5.2 背景 2

6. **Overview of Investigational Medical Devices** 9

6.1 Product Overview 9

6.1.1 Development History 9

6.1.2 How it works 9

6.1.3 Shape and Structure (Appearance) 9

6.1.4 Shape and Structure (Attributes) 12

6.1.5 Commodities 16

6.1.6 Performance 17

6. 2Purpose and Research 17

6.2.1 Intended Use (Indications)

…………………………………………………………………………………… 17

6.2.2 Prior Research …………………………………………………………………………………………… 17

7. **Inclusion Criteria, Exclusion Criteria, and Number of People Covered by an Investigational Medical Device or Included in a Control Group and Rationale for Participation in a Clinical** Trial……………………………………………………………… 18

7. 1Subject Selection Criteria ………………………………………………………………………………… 18

7.2Exclusion Criteria for Subjects ………………………………………………………………………………… 18

7.3 Number of subjects and rationale for calculations ……………………………………………………………………………… 18

7.3.1 Number of subjects 18

7.3.2 Calculation Basis 19

8. **Clinical Trial Duration** 20

9. **Clinical Trial Methods** 21

9.1Designing a Clinical Trial 21

9.2 Clinical Trial Methods 21

9.2.1 Preparing subjects 21

9.2.2 Setting up a Test Group/Control Group 22

9.2.3 Randomization Methods 23

9.2.4 Double Blindfold 23

9.3How to use medical devices 24

9.3.1 Preparation before use 24

9.3.2 Application Area and Usage Time 24

9.3.3 How to Operate or Use 25

9.3.4 How to store and care for after use 26

9.3.5 Cautions for Use 26

9.3.6 How to Use a Sham Device 27

9.3.7 Combination Therapy 27
10. **Observations · Clinical Examination Items and Observation Methods** 28
10.1 Clinical Trial Timeline 29
10.2 Observations and Clinical Tests 29
10.3 Observational Testing Methods 32
11. **Possible side effects and precautions for use** 33
11.1 Possible side effects 33

11.2 Cautions for use 34

12. **Stop and drop criteria** 35

12.1 Stopping Criteria 34

12.2 Elimination Criteria 34

12.3 Handling Stops and Dropouts 35

13. **Evaluation criteria, methods, and interpretation of validity** 35

13.1 Primary Validity Scale 35

13.2 Secondary Validity Scale 36

14. **Criteria for evaluating safety, including adverse events, and how they are evaluated and reported** 36

14.1 Definition of an adverse event, adverse reaction 36

14.2 Evaluation of Adverse Events 37

14.3 Causation with Clinical Devices 37

14.4 Evaluation Criteria 38

14.5 Evaluation Methods 39

14.6 Reporting adverse events 39

14.7 How to report 39

15. Informed **consent form (attached)** 40

16. **protocol for victim compensation** 40

16.1 Compensation Requirements 40

16.2 Reasons for Exclusion 41

16.3 Compensation Criteria 42

16.4 Compensation Procedures 42

16.5 Coverage 43

17. **matters concerning the care of subjects after the study** 43

18. **risks and benefits to human subjects** 43

19. **Measures to protect the safety of human subjects** 44

19.1 Clinical Trial Sites 44

19.2 Institutional Review Board (IRB) 44

19.3 Investigator 44

19.4 Sponsor 45

19.5 Monitoring 45

19.6 Changes to the Protocol 45

19.7 Informed Consent 46

19.8 Confidentiality of Subject Records 46

19.9 Retention of records 47

19.10 Processing Specimens 47

20. **what else is needed to conduct clinical trials safely and scientifically** 47

20.1 **Case notes** 47

20.2 **Monitoring** 47

20.3 The History and archiving 48
20.4 Reports 提交 and publishing 48
20.5 Contracts 48

Protocol Summary

**Study title**	To evaluate the **safety and efficacy of the** medical light irradiator **BELLALUX Lite on wound healing in** patients with **mild pressure ulcer co-morbidity, a single-center, double-eye** **Prospective exploratory clinical trial with a masked, randomized, parallel design (sham device control)**
**Test Sponsor**	Link Optics, Inc.
**Clinical sites and Testers**	.
**Examination syllabus number**	loc_bellaluxlite_01
**Clinical trial design**	Single-center, randomized, parallel design, prospective exploratory clinical trial
**Number of subjects**	38 participants (19 experimental, 19 control, 15% dropout rate)
**Clinical Trial Purpose**	For patients with mild pressure ulcer co-morbidity, the medical light irradiator 'BELLALUX Lite' was used as the Exploratory evaluation of safety and efficacy for wound repair
**Trial Period**	30 months from clinical start date
**Subject selection criteria**	(1) Patients with stage 2 pressure ulcers (based on NPUAP guidelines) in the hip area.<br>(2) 13 years of age or older
**Subject exclusion criteria**	(1) Pregnant and nursing women<br>(2) Prior hip surgery (however, convalescent subjects may be enrolled at the discretion of the investigator)<br>(3) People with osteitis<br>(4) Isolated with resistant strains<br>(5) Unable to be in the supine position for more than 30 minutes<br>(6) Persons requiring continuous use of immunosuppressive drugs or steroids for internal medical conditions (but may be enrolled if, in the opinion of the investigator, these agents have a low impact on immune function).<br>(7) Have a light sensitivity (such as photosensitivity) or are taking medications related to light.<br>(8) Any other reason that the investigator believes the trial cannot be conducted properly.<br>The Judged

**Test Device/Control Device**	**Test device**: BELLALUX Lite<br>**Control device (Sham)**: Pseudo-medical device with the same geometry as the test device.<br>- How to use the control device: The control device is a device with the same shape as the test device, and the light irradiation part is made to look similar to the light irradiator irradiated by the test device using LEDs of 12mW/cm2 or less, and other appearance and operation.<br>The method is the same as for the tester.
**Research Methods**	For subjects who signed the informed consent form of their own volition, after conducting the necessary examinations and tests according to the protocol, reviewing the inclusion and exclusion criteria, and deeming them suitable for this study, educating them about the study schedule and methods. Subjects will be irradiated with 4 different wavelengths of LEDs at maximum power (5 levels (90 mW/cm^2) for a maximum duration (25 minutes) at a distance of 15 cm from the pressure ulcer in the buttock area. LED irradiation will be **performed 3 times per week**, with one week of treatment completed.<br>This is followed by a medical examination and wound assessment. This is **done for a total of 4 weeks**.
**Evaluation Variables**	(1) Validation<br>1) Primary efficacy measures: wound size and degree of re-epithelialization<br>2) Secondary validity measure: immunochemical markers on tissue examination<br>(2) Assessing **safety**<br>1) Anomalies<br>2) Physical exam and vital signs
**Analysis Methods**	- **Wound size and degree of re-epithelialization**: calculate the wound size and degree of re-epithelialization of the control and experimental groups at 0 and 4 weeks, respectively, against time, and analyze the differences between the two groups using a paired t-test.<br>- **Immunochemical markers on tissue examination**: pro-inflammatory cytokine (IL-1, 6)/anti-inflammatory cytokine (IL-4, 10, 13) ratio at 0 and 4 weeks.<br>Analyzes progression to the proliferation phase by comparing control and experimental groups

1. **Name and location of the study site**

2. **Full name and title of the principal investigator and co-investigators of the study**

3. **Full name and title of the administrator managing the investigational medical device**

4. **Full name and address of the person conducting the study**

5 **The purpose of clinical trials and background**

5.1 Purpose

This is a **single-center, double-blind, randomized, parallel-group, prospective exploratory study (compared to a sham device) in** patients with **mild pressure ulcers to** evaluate the safety and efficacy of the **medical light irradiator BELLALUX Lite on** wound healing.

- **Primary Objective**: The primary objective **of** this study is to compare wound size, degree of re-epithelialization, and immunochemical markers after 4 weeks of application of **the** investigational device in the treatment of mild pressure ulcers.

Validated.

- **Secondary objective**: **To evaluate** the safety of **BELLALUX Lite** by means of biomedical examinations and adverse events assessed by the investigator before application of the investigational device and 4 weeks after application.

5.2 Backgrounds

Pressure ulcers are localized damage to the skin and subcutaneous fat caused by a combination of pressures, including shear and frictional forces, and are most commonly caused by objects such as bony prominences or medical devices. Studies have reported morbidity rates ranging from 0% to 75%, with an average of 6.3%.[1] Pressure ulcer morbidity is particularly high in intensive care unit patients (8.8% to 12.1%) and patients with spinal cord injury (33% to 60%).[2]

While no national statistics are available, U.S. statistics show that 2.**5 million people a year suffer from pressure ulcers,** and **60,000 people a year die from** them. This is more than the number of deaths from the flu (5.6 million) and suicide (44,000). As a societal problem**, pressure ulcers cost society an estimated $11.6 billion annually**. The cost per person ranges from $500 to $150,000.[1-3]

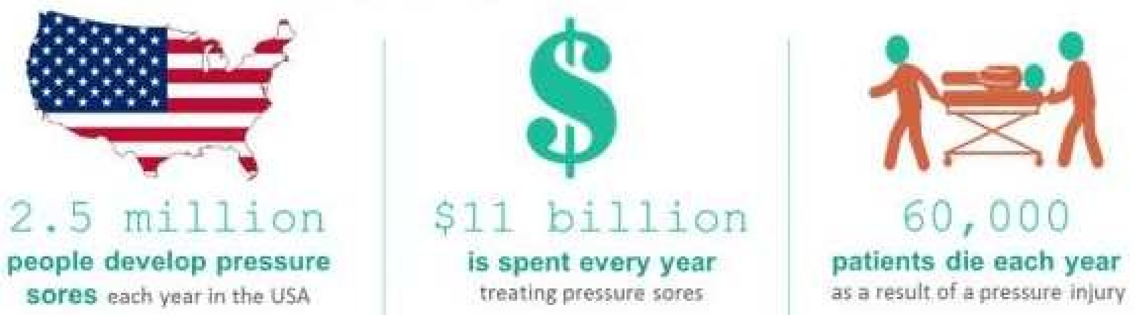

Figure 1. Level of morbidity and annual societal cost of pressure ulcers (US statistics)

Normally, the average pressure in capillaries is around 32mmHg, and ischemic necrosis develops if the pressure is double that (70-80mmHg) for more than 2 hours. Doubled pressure acts on the tissue and accelerates the progression of necrosis, especially when accompanied by shear and friction forces. Pathogenetically, pressure ulcers can occur anywhere on the body, and although studies vary, they are most common in the hip (28.3%), heel (23.6%), and sciatic (17.2%).[2]

1) NPIAP-EPUAP-PPPIA. Prevention and Treatment of Pressure Ulcers/Injuries: Clinical Practice Guideline. The International Guideline 2019

2) Robert K, Juan LR, Jeffery E. Pressure Sores. Neligan 4th edition. Elsevier. 2016 Vol. 4;350-380.

3) William VP, Benjo AD. The National Cost of Hospital-Acquired Pressure Injuries in the US. Int Wound J. 2019;16(3):634-640.

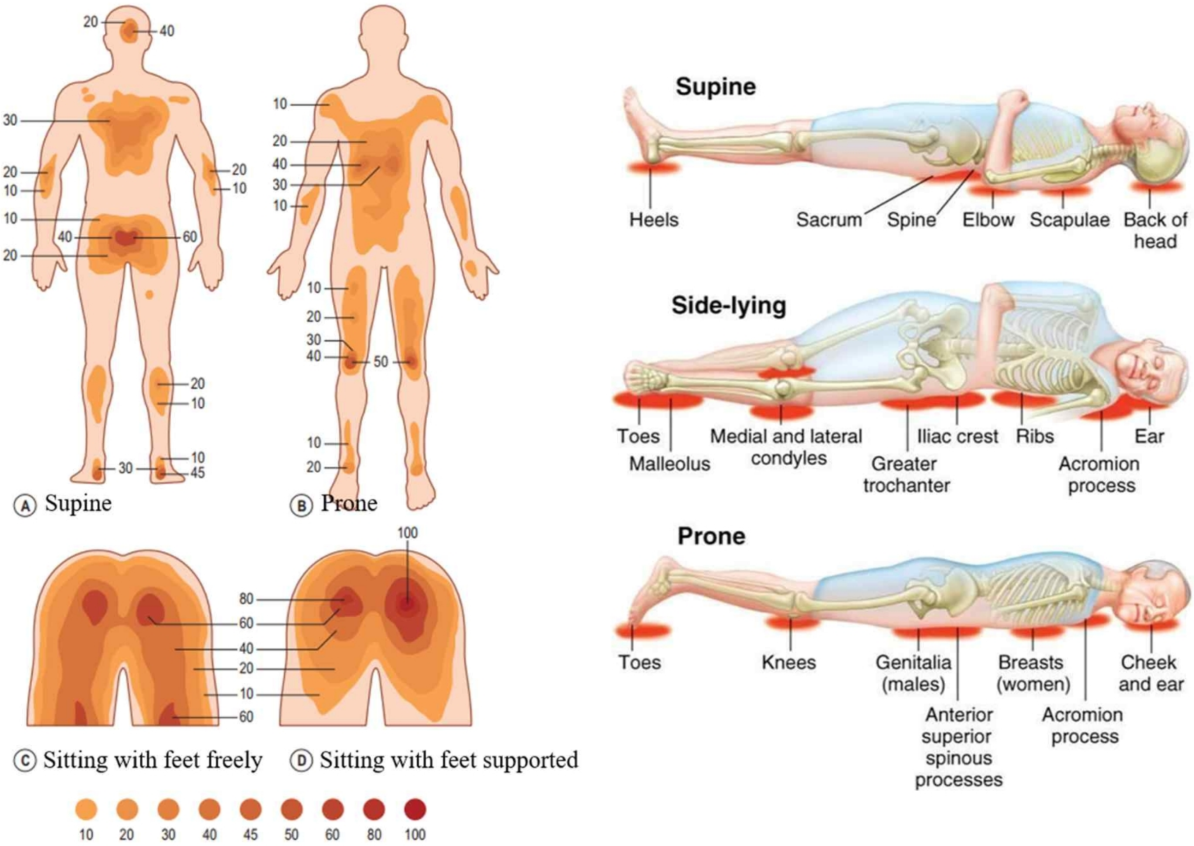

Figure 2. Soft tissue loading pressure and pressure ulcer predisposition based on posture.

Pressure ulcers can be classified in a variety of ways, but the most commonly used is the staging system published by the National Pressure Ulcer Advisory Panel (NPUAP). The **NPUAP classifies pressure ulcers into** six stages based on depth, including two unclassified pressure ulcers.[1] **NPUAP stage 2** shows **skin** damage, and stage 3 and above shows skin tissue necrosis and requires a limbectomy.

Pressure ulcers are a classic example of a chronic wound with a sustained inflammatory response, and while **recovery** time varies depending on the extent and depth of the tissue lost, **1-3 months for mild cases** and at **least 6 months for moderate or more severe cases.**
~1 year or more. Recovery, however, is not guaranteed, but can be achieved if the direct causative pressure is relieved and the indirect causative factors, such as internal medicine management and nutrition, and ongoing wound care, that have not been addressed, leading to sustained pressure, are addressed.

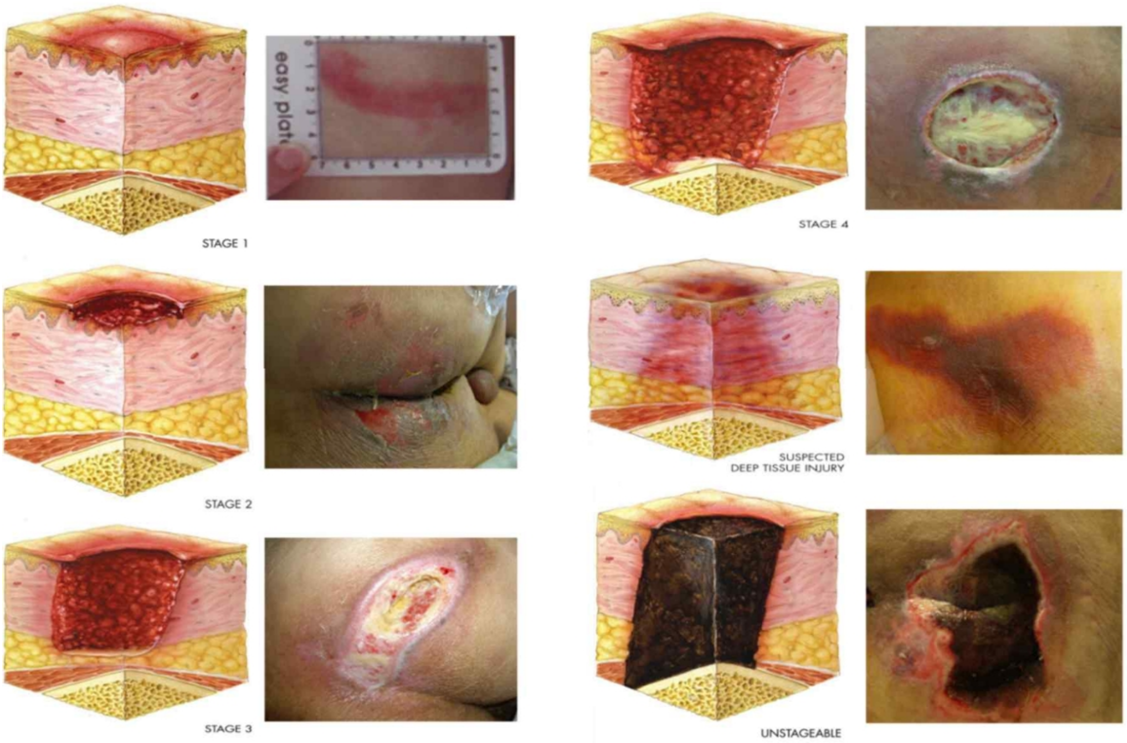

Figure 3. NPUAP Pressure Ulcer Classification

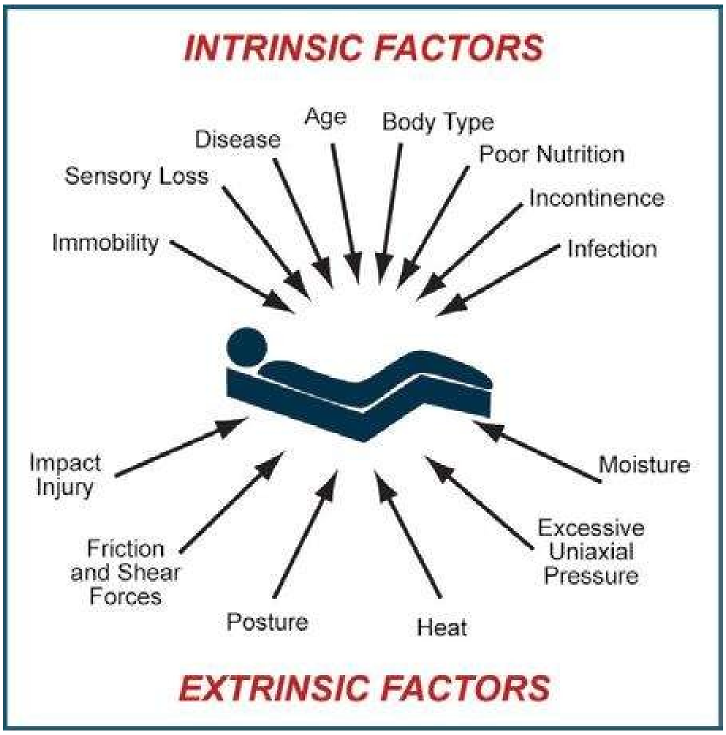

Figure 4. Multifactorial causes of pressure ulcers

This **multifactorial, high societal cost of pressure** ulcers is a **global issue, and** medical experts from the United States, Europe, and the Pacific Rim (National Pressure Injury Advisory Panel - European Pressure Ulcer Advisory Panel - Pan Pacific Pressure Injury Alliance) have published three international guidelines on pressure ulcers since 2009 (first edition 2009, revised 2014, 2019). The guidelines state that the **best treatment for pressure ulcers is prevention** and **emphasize the importance of positioning**. Depending on the study, up to 95% of pressure ulcers are preventable.[1]

However, once skin necrosis has occurred, it can be accompanied by infection, so limbectomy should be performed first. After limbectomy, the defect can be surgically repaired with flaps, but if general anesthesia cannot be performed, **conservative treatment is the** only option. In this case, it is **very important to control inflammation and promote the wound healing process**. To date, many wound coverings have been developed, and wound healing can be facilitated by choosing the right antiseptic for the condition of the wound. However, each treatment has its own limitations and there is no single method for the conservative treatment of pressure ulcers.

Photobiomodulation therapy, which involves irradiating the affected area with low-powered light to induce healing, has also been reported to help heal wounds by promoting oxidative modulation and growth factors. In addition, it is said to have anti-inflammatory, analgesic, and sterilizing effects, and there are also studies that have verified the effectiveness of photobiomodulation therapy for pressure ulcers.[4,5] However, the exact mechanism of photobiomodulation therapy is still unclear.

A systematic review of five studies reported in 2020 by Francislene FCP et al. found that **658 nm wavelengths produced significant results in pressure ulcer healing**, **but** 808 nm or 990 nm did not show significant differences. Energy densities of 1 $^{J/cm2}$ or 4 J/cm2 were used in most studies, and treatments were administered 3-5 times per week × 4-6 weeks. Treatment effects were analyzed by quantitative analysis of wound size and depth, degree of re-epithelialization, growth factor secretion, and cytokines. However, when the studies were analyzed for bias, very few were complete. In addition, there is no analysis of the healing process of chronic wounds, only the final outcome.[5]

---

4) Chen C, How WH, Chan ESY, Yeh ML, Lo HLD. Phototherapy for Treating Pressure Ulcers. Cochrane Database Syst Rev. 2014;11(7):CD009224.

5) Francislene FCP, Jorge VCF, Hellen R, et al., Effect of Photobiomodulation on Reparing Pressure Ulcers in

Adult and Elderly Patients: A systematic Review. Photochem Photobiol. 2020;96(1):191-199.

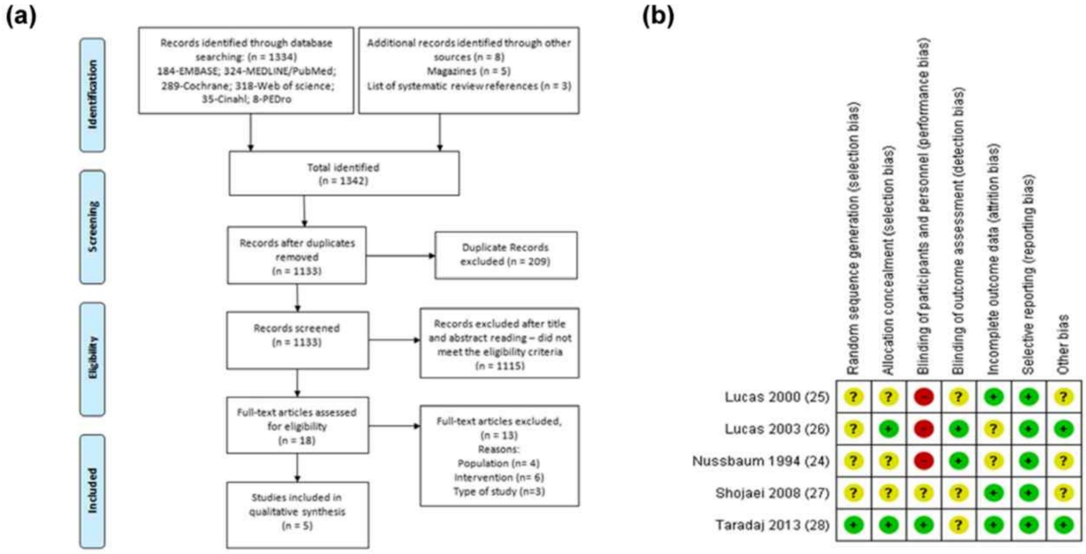

Figure 5. (a) Flowchart of study selection for systematic analysis. (b) Bias survey table of the five selected studies.

The BELLALUX Lite, which will be used in this clinical trial, was certified by IECEE-CB (the world's first international system for mutual recognition of product safety test reports and certifications for electrical and electronic equipment, devices and components) in July 2019 and is a **class 2 medical combination stimulator**. This product is an improved and lighter version of its predecessor, BELLALUX (model name: RED&AMBER- MD1), which emits four wavelengths, BLUE (460 nm) and NIR (850 nm), in addition to RED (630 nm) and AMBER (595 nm) wavelengths.

A preclinical trial using rats to verify the efficacy of the systemic model 'BELLALUX' in treating pressure ulcers was **conducted at our center in 2019**. The results showed that the Stimulation Index (SI) was 0.7 ~ 1.3 compared to the control group in the skin sensitization test of local lymph nodes, which is lower than the SI of 1.6, which is considered a skin sensitizer, **proving safety**.

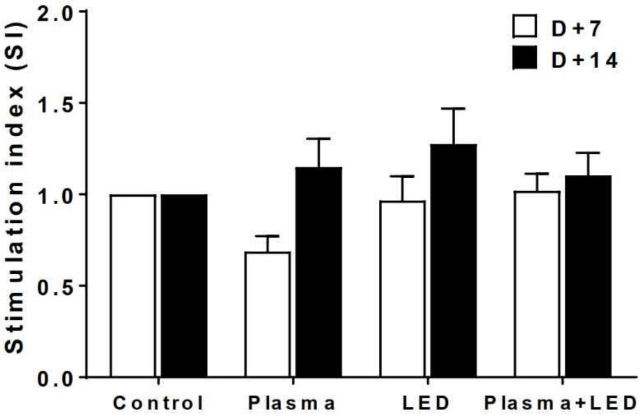

Figure 6. Preclinical test results for safety evaluation (Photobiology Laboratory, College of Medicine, Dankook University)

The efficacy evaluation was based on the recovery from infectious wounds, and it w a s found that the wound size was reduced by a significant difference in the groups that were irradiated with plasma and light respectively compared to the control group, and the recovery was faster in the experimental group that was irradiated with plasma and light simultaneously.

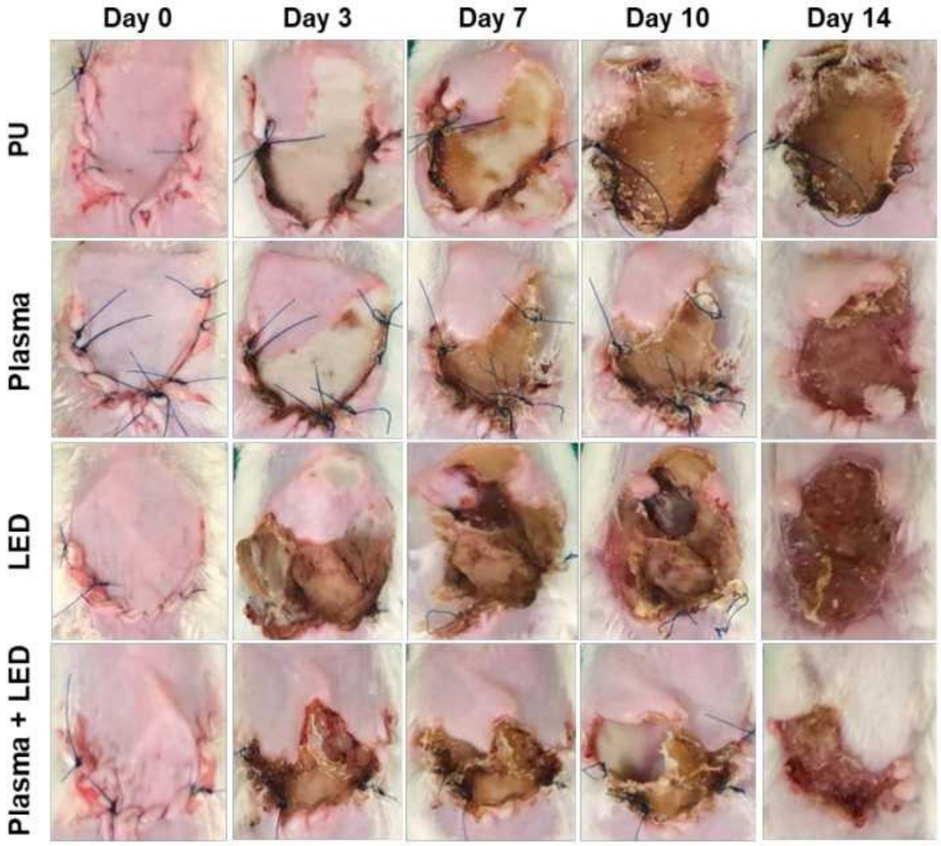

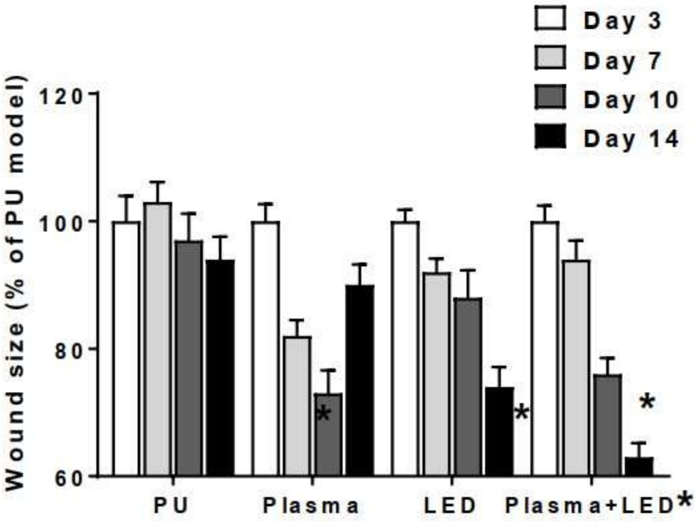

Figure 7. Preclinical test results for efficacy evaluation (Photobiology Laboratory, Dankook

University School of Medicine)

Therefore, there is a **need to evaluate the effectiveness and safety of the medical device clinically, and this clinical trial is planned**. In the case of the plasma used in the preclinical trial, the sterilizing power is generated by the ozone generated, which is effective for infectious wounds such as the above experiment, but it is not suitable for clinical trials because the generation of such ozone may be potentially dangerous for humans. In the **case of this investigational device (BELLALUX Lite), which is a successor to the model used in preclinical trials, the BLUE wavelength of 460 nm has also been added to provide enhanced sterilization**. The sterilizing power of BLUE wavelength has been studied in our laboratory as follows.

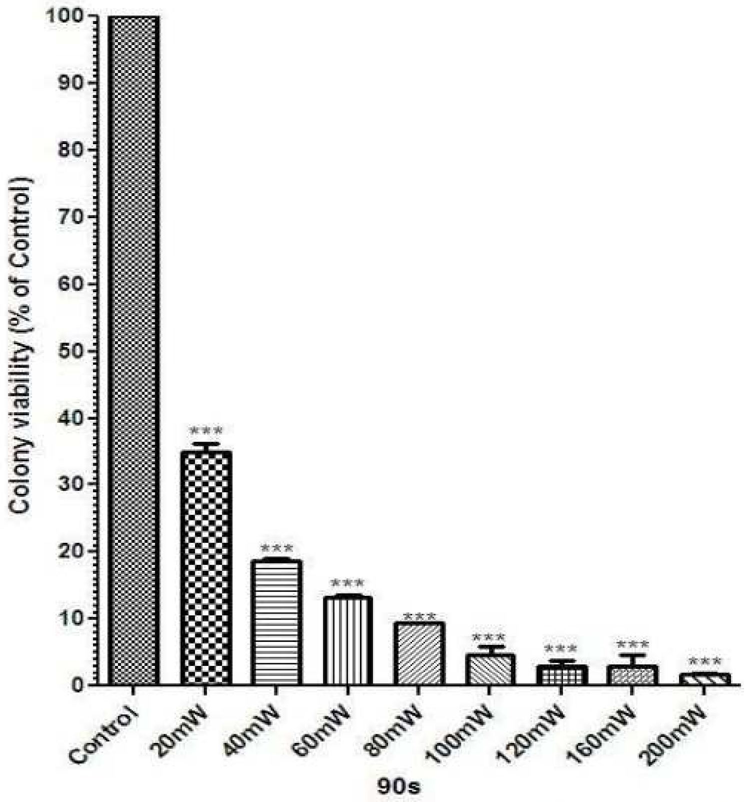

Fig. 6 90s, 20mW, 40mW, 60mW, 80mW, 100mW, 120mW, 160mW, 200mW로 조사한 *Propionibacterium acnes* 균의 viability

Figure 8. Survival rate of the anaerobic bacterium Propionibacterium acnes in response to 408 nm wavelength.

Therefore, this device can be expected to have sufficient bactericidal power (BLUE wavelength) and wound healing power (RED+AMBER wavelength) for pressure ulcers, which are typical of chronic wounds, and clinical trials with this device are warranted.

In addition, the trial is minimal risk as the experimental group will receive the additional benefit of the LED therapy device while the control group will receive their usual conservative care (antimicrobial disinfection).

6 Overview of Investigational Medical Devices

6.1 Product Overview

6.1.1 Development History

Ltd. developed the product by confirming that it is effective in treating pressure ulcers through preclinical trials as a class 2 medical combination stimulator item that combines low-power light irradiators and infrared irradiators.

6.1.2 Howworks

This product is a class 2 medical combination stimulator that combines a low-power light irradiator and an infrared irradiator, and when electric energy is applied, it emits energy in the form of light through a visible light LED lamp with a wavelength of visible light [RED (630nm), AMBER (595nm), BLUE (460nm)] and a near-infrared lamp with a wavelength of near-infrared light (NIR, 850nm), and this light is absorbed by cells and activates cell function, so it is a device developed for use in treating skin diseases and treating bedsores. This product is composed of an irradiation part and a main body part. The irradiation part is attached with a lamp and emits visible and infrared light when supplied with power, and functions to adjust the angle and direction of the irradiation part through the irradiation part connection, and the main part supports the irradiation part and enables height adjustment of the product.

6.1.3 Appearance and structure (appearance)

6.1.3.1 Appearance

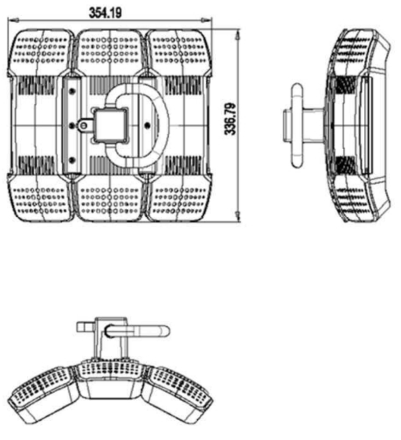

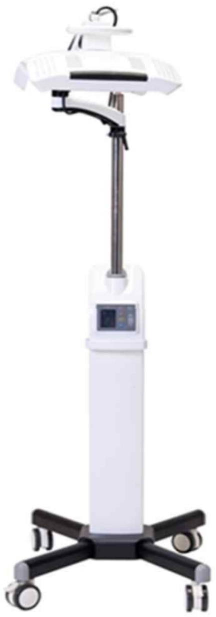

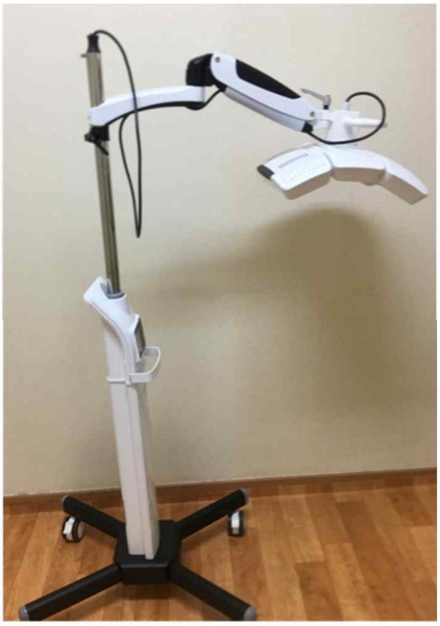

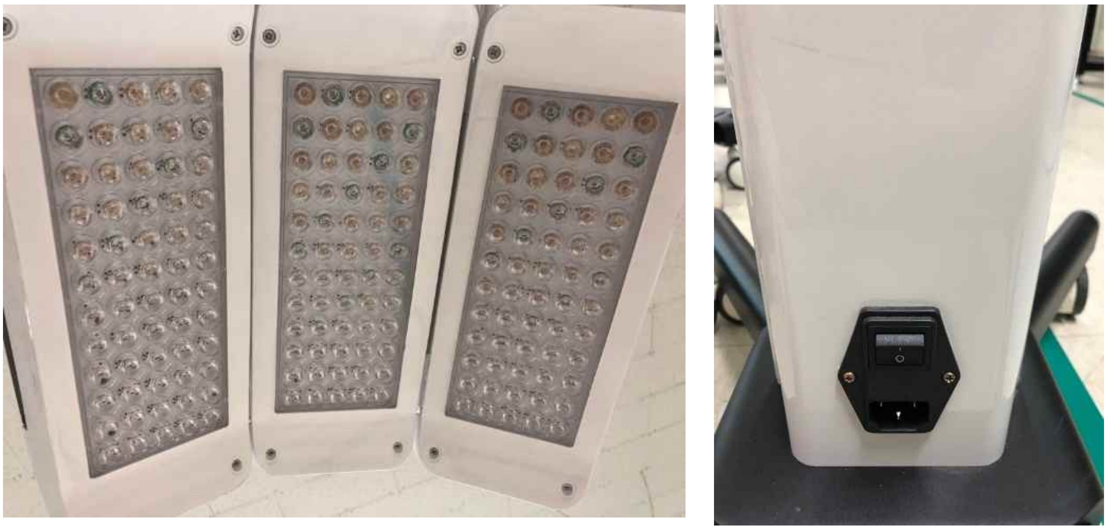

Figure 9. 'BELLALUX Lite' and product appearance

6.1.3.2 Appearance Description

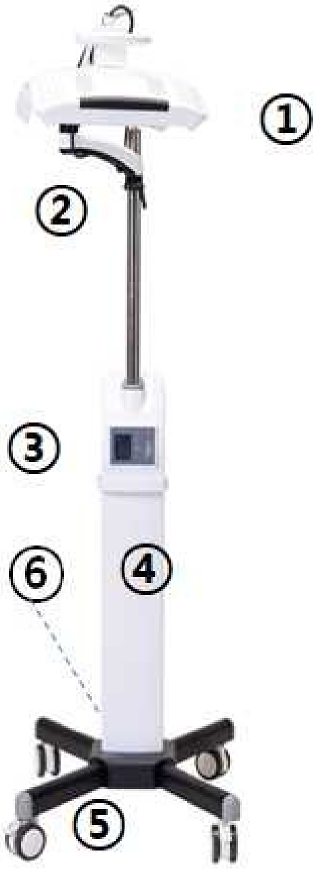

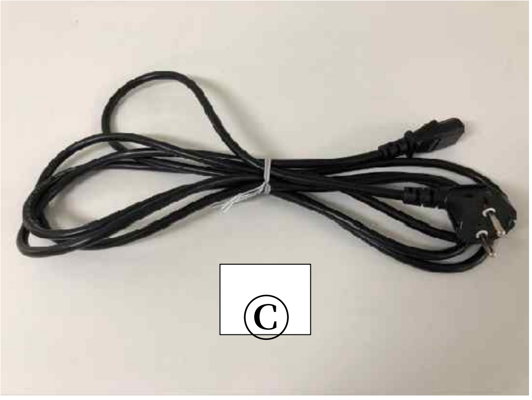

Num ber	Name	功能					
1.	Investigations	Light Source - LED Light Output [Output in mW/cm2 at 15 cm].					
		Wavelength / Number of LEDs	Step 1	Step 2	Step 3	Step 4	Step 5
		Blue (460 nm) /45 pcs	0.80	2.20	3.60	5.10	8.40
		Amber (595 nm) /45 pieces	2.00	4.50	7.00	9.00	12
		RED (630nm) / 45 pcs	3.60	8.80	13.80	19.00	30.00
		NIR (850 nm) / 45	5.60	12.40	21.00	28.00	40.00
2.	Adjustments	Adjust the height and orientation of the light source					
γ	LCD Screen & Buttons	LCD screen and operation buttons					
④	Body	The body of the product					
⑤And	Movable Pedestal	Move the device while supporting the base					
6	Power	Product power					
⑦ â	Power cord	Product power cord					

6.1.3.3 Button Description

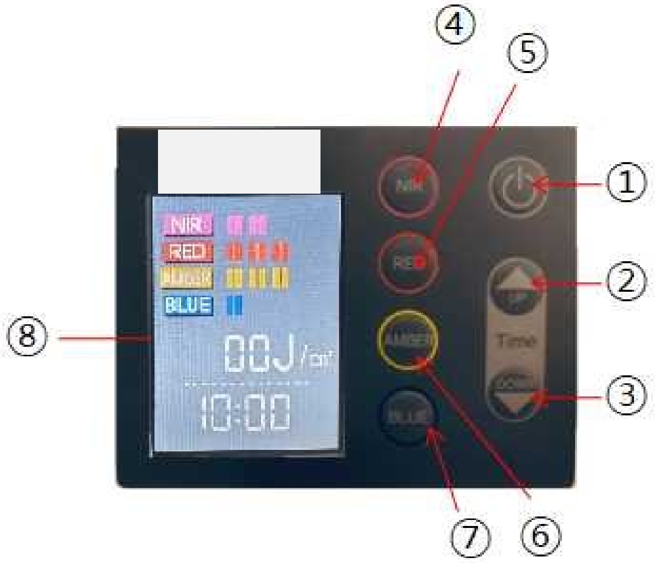

- External Actuation Button Descriptions

Number	Name	功能
1.	Operation buttons	Device On/Off switch
2.	Time adjustment up button	Can be incremented in 5 minute increments up to 25 minutes
γ	Time adjustment down button	Can be lowered in 5 minute increments up to a minimum of 5 minutes
④	NIR	Switch to adjust the NIR wavelength<br>Can be set from 0 to 5 levels
⑤And	RED	Switch to adjust the RED wavelength<br>Can be set from 0 to 5 levels
6	AMBER	Switch to adjust the AMBER wavelength<br>Can be set from 0 to 5 levels
⑦ â	BLUE	Switch to adjust the BLUE wavelength<br>Can be set from 0 to 5 levels
⑧ â	LCD Screen	LCD screen Displays the current status

6.1.4 Shape and Structure (Attributes)

6.1.4.1 How it works

This product is used by irradiating the visible and infrared light energy from the LED (Light Emitting Diode) to the affected areas of skin diseases and pressure ulcers of the human body when electrical energy is applied to the lamp made using the LED (Light Emitting Diode) through the product power supply.

6.1.4.2 Electrical ratings

- Rated voltage : 220V AC
- Rated frequency : 60Hz
- Power consumption: 200W

6.1.4.3 Classification by type and degree of protection against electrical shock

- Class 1 device, no mounting

6.1.4.4 Safeguards

- Fuse (2A): Automatically cuts off the power to the device when the power is short-circuited or there is an abnormality in the device.

6.1.4.5 Embedded software

- Model Name: Bellalux Lite FW
- Name: Lite Light Source Control Program
- Software safety rating: A
- Medical Device Cybersecurity Safety Rating: Medium
- Algorithms, Structure, and Key Features

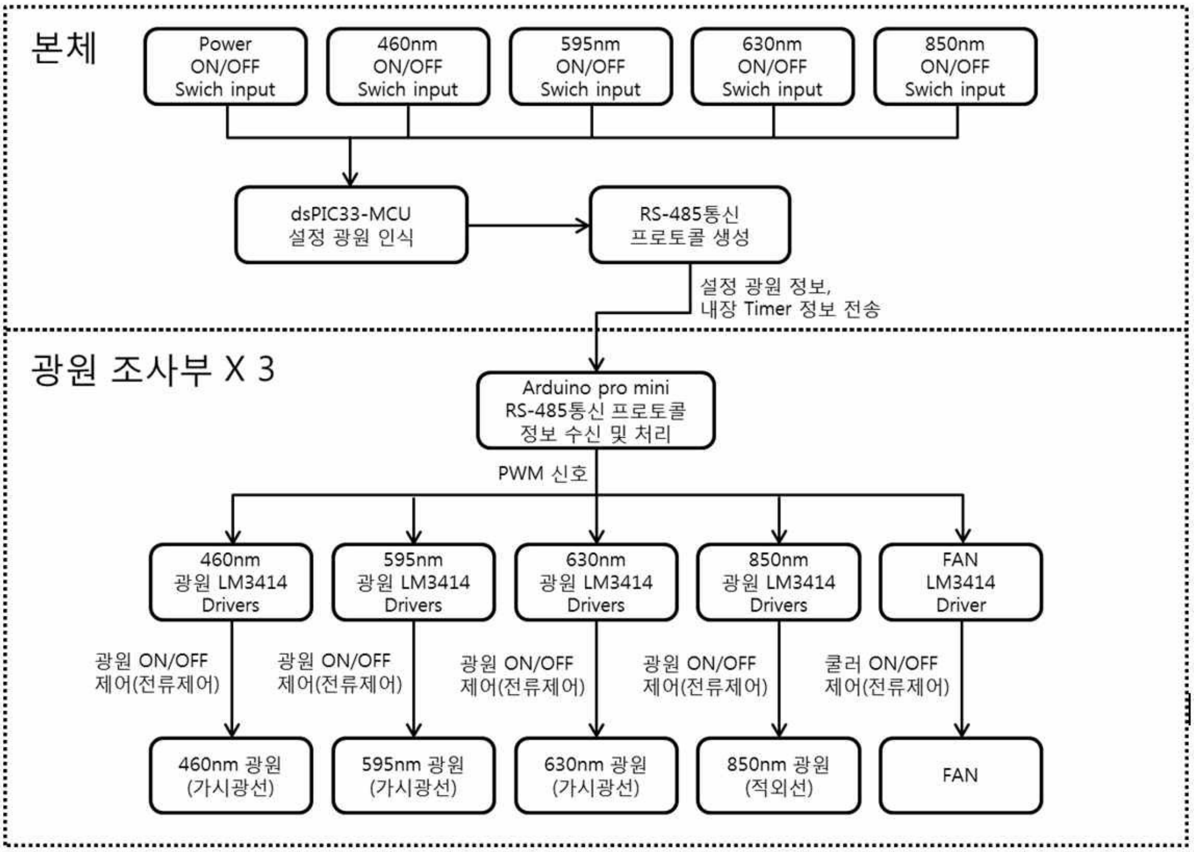

Figure 10. Schematic of the 'BELLALUX Lite' algorithm

- Electrical Schematic

본체 전원부

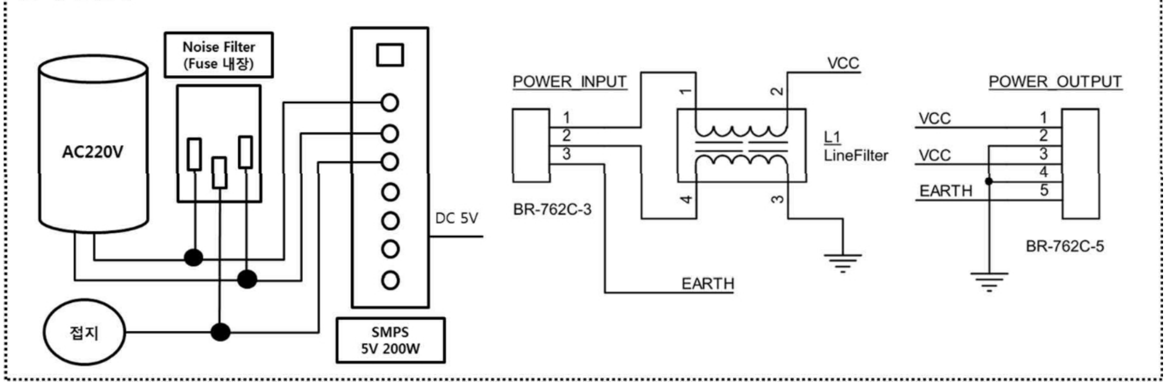

본체 스위치부

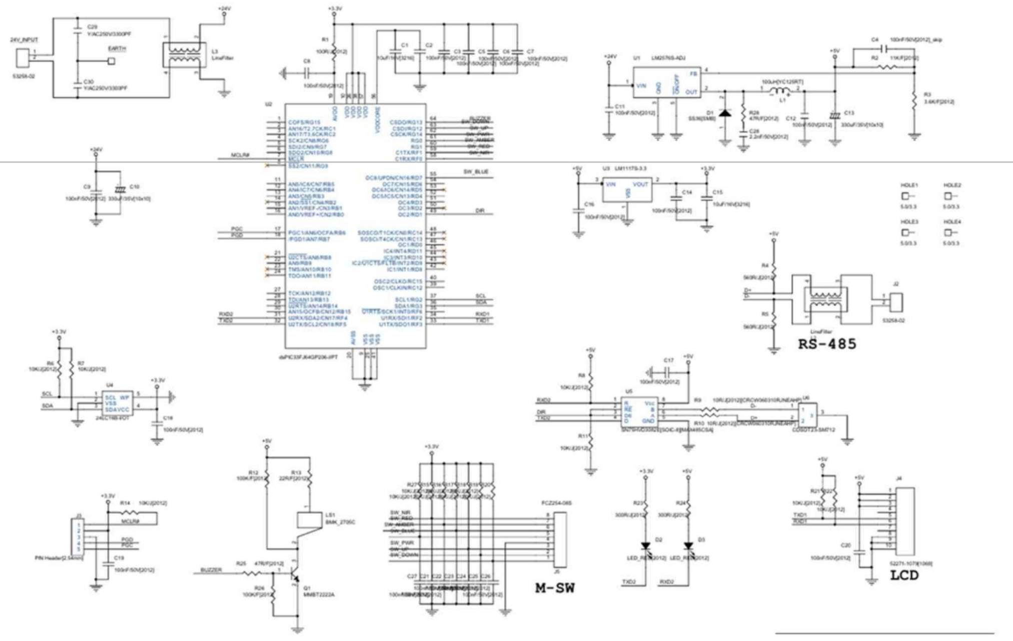

본체 광원부1

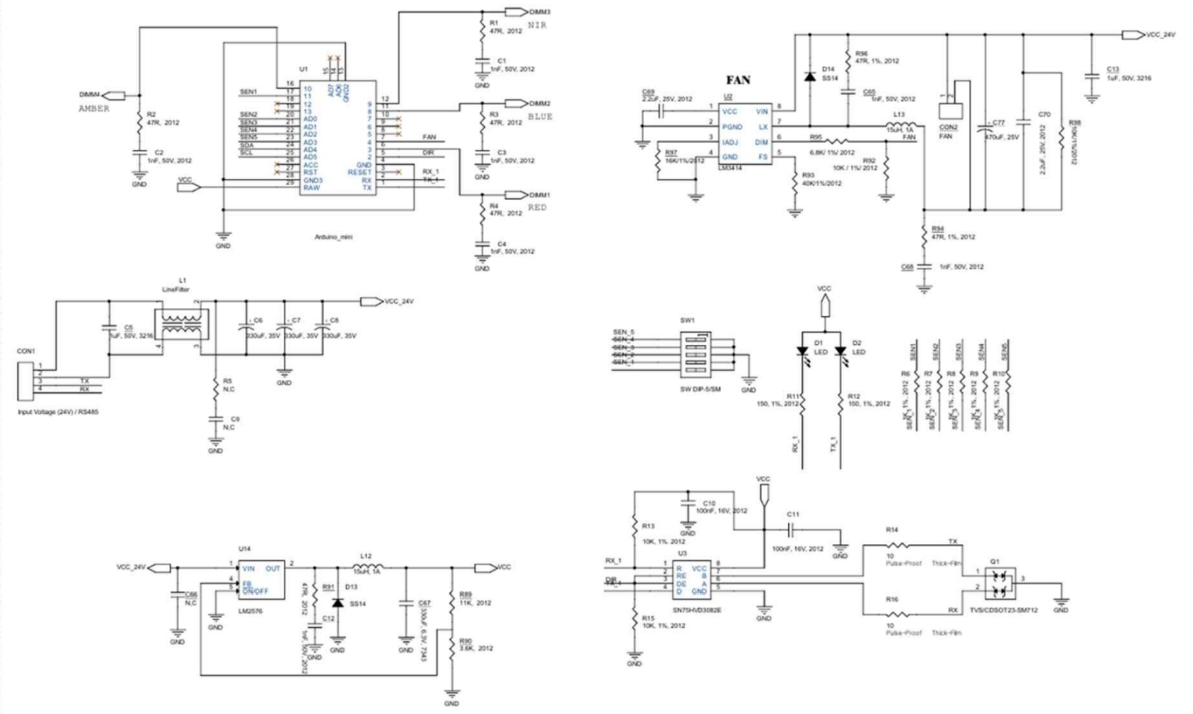

본체 광원부2

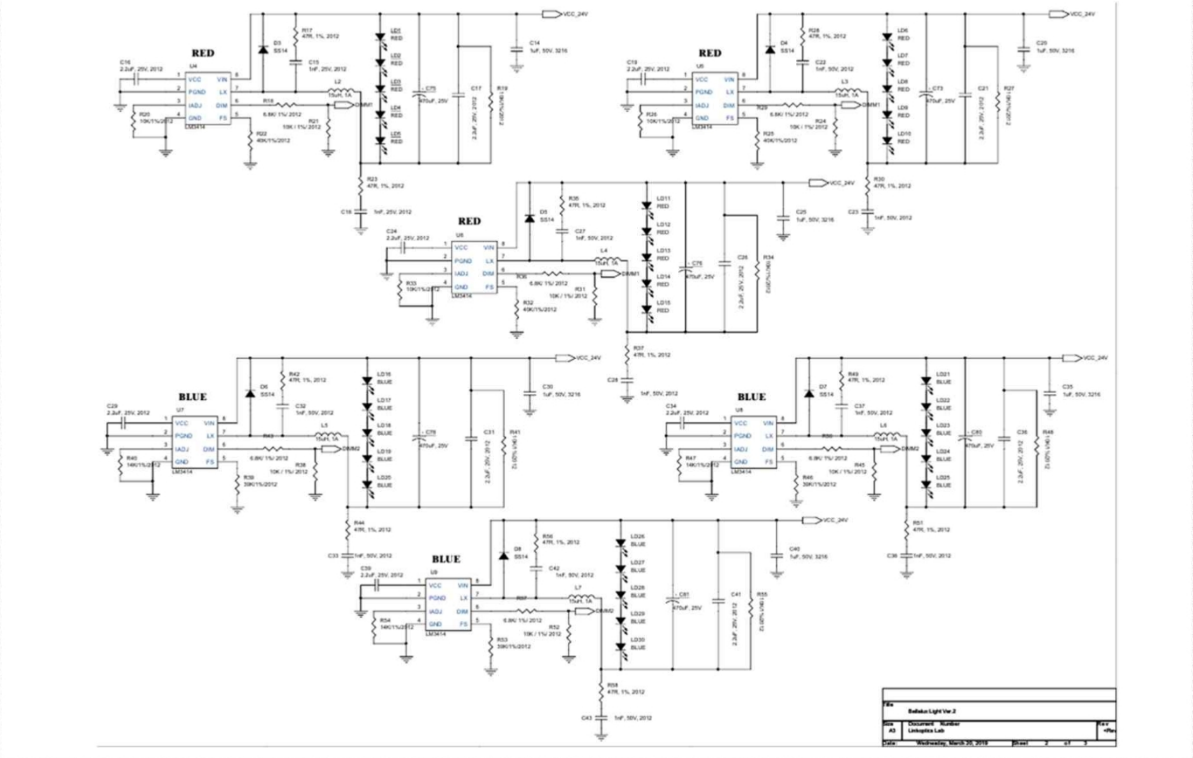

Figure 11. 'BELLALUX Lite' electrical schematic

6.1.5 Commodities

Serial Number	Part name		Part Number	Specification or attribute	Quantity	Remarks
1	Investigations	LED	MD-032M -PART-01	595 nm<br>IEC62471<br>cb (kr-keti4002)	15	
2		LED	MD-032M -PART-02	460 nm<br>IEC62471<br>cb (kr-keti4002)	15	
3		LED	MD-032M -PART-03	630nm<br>IEC62471<br>cb (kr-keti4002)	15	
4		LED	MD-032M -PART-04	850 nm<br>IEC62471<br>cb (kr-keti4002)	15	
5		PCB	MD-032M -PART-05	FR-4<br>V-0	3	
6		Investigations Appearance	MD-032M -PART-06	head-l(t,b)	3	
7	控制部	Switch PCB	MD-032M -PART-07	FR-4<br>V-0	1	
8		Switch	MD-032M -PART-08	5 Button	1	
9	Display	LCD	MD-032M -PART-09	2.8" Module	1	
10	Exterior	Body Appearance	MD-032M -PART-10	1) Plastic: ABS, HB, UL (E67171)<br>2) Light radiation: PP, V-0, UL (E119841)	1	
11	Power	SMPS	MD-032M -PART-11	Input:100-240VAC, 3.6-1.0A<br>Output: 36VDC, 5.7A<br>CB(DK-49337-A1-UL)	1	
12		Noise Filter	MD-032M -PART-12	250A~,2A, 50/60Hz<br>KC (SH03003-5007B)	1	
13		Power cord	MD-032M -PART-13	250v 10a 3m kc<br>(hh01080-16011a)	1	
14	Software		-.	V1.00		

6.1.6 Performance

6.1.6.1 Radiant Power (15 cm from the irradiator) [Output unit (mW/cm2)].

Wavelength	Step 1	Step 2	Step 3	Step 4	Step 5
**Blue**	0.80	2.20	3.60	5.10	8.40
**Amber**	2.00	4.50	7.00	9.00	12
**RED**	3.60	8.80	13.80	19.00	30.00
**NIR**	5.60	12.40	21.00	28.00	40.00

* Must be within ±20% of each criterion.

6.1.6.2 Output time: up to 25 minutes, adjustable in 5 minute increments

6.1.6.3 Light wavelength : BLUE(460nm±10nm), AMBER(595nm±10nm), RED(630nm±10nm), NIR(850nm)
±10 nm)

6.1.6.4 Irradiation area: 15cm away from the LED light source

6.1.6.5 Safety device: blown fuse cuts off power in case of overcurrent

6.2 Purpose of Use and Prior Research

6.2.1 Purpose of use (indications)

It is **used to treat skin diseases, treat pressure ulcers, and relieve pain by irradiating the affected area with visible and infrared light.**

6.2.2 Research

6.2.2.1 Treatment of skin diseases: In a 2013 clinical trial of 40 patients at the dermatology departments of Seoul National University Hospital and Chonnam National University Hospital, LED phototherapy with a wavelength of 595 nm showed improvements in skin elasticity, melanin pigmentation, and skin erythema.[6]

6.2.2.2 Pressure Ulcer Treatment: Following a preclinical trial in 2019, this exploratory clinical trial aims to provide evidence for the treatment of pressure ulcers.

6) Moon GR, Lee JB, et al, The Effectiveness of Light Emitting Diodes with 592 nm Yellow Light for Korean

Photoaged Skin. Korean J Dermatol 2015;53(9):677-683.

7 Inclusion and exclusion criteria for persons who are subject to an investigational medical device or who are included in the control group and participate in a clinical trial, including the number of persons and their rationale.

Inclusion and exclusion criteria were selected based on a 2014 Cochrane review article by Chen C. et al. and a systematic review of five articles reported in 2020 by Francislene FCP et al.

7.1 Subject's selection criteria

(1) Patients with stage 2 pressure ulcers (based on NPUAP guidelines) in the hip area.

(2) 13 years of age or older

7.2 Subject's Exclusion Criteria

(1) Pregnant and nursing women

(2) Prior hip surgery

(3) Recurrent pressure ulcers

(4) People with osteitis

(5) Isolated with resistant strains

(6) Unable to be in the supine position for more than 30 minutes

(7) People who need to take immunosuppressants or steroids for a medical condition.

(8) Have a light sensitivity (such as photosensitivity) or are taking medications related to light.

(9) Any other person who, in the opinion of the investigator, is unable to properly conduct the study.

7.3 Number of subjects and rationale

7.3.1 Number of subjects

Aim for **16 subjects per** endpoint **arm**, and account for a 15% dropout rate.
38 subjects, **19 per control and experimental group, for a total of 38 subjects.**

7.3.2 Rationale

This study is an **exploratory trial** and is intended to provide a **basis for statistical validation of a confirmatory trial**. A systematic review of the effectiveness of photobiological therapy in the treatment of pressure ulcers by Francislene FCP et al. published in 2020 found that the five reviewed trials each had between 6 and 40 patients per arm, with an average of 15.8 patients per arm. We selected 16 subjects per arm as the minimum standard for this trial, with a predicted dropout rate of 15%. We will **target 19 subjects per group for the final evaluation**.

The dropout rate was **selected based on the fact that out** of 250 patients treated by a single professor (Dr. Namkyu Lim, Department of Plastic Surgery) over a **recent 2-year period,** 45 patients died within the study period, resulting in a mortality rate of **18%.** As the mortality rate is relatively high in patients with moderate and severe pressure ulcers**, we** predicted that the **mortality rate would be lower in the subjects included in this study and** set a dropout rate of 15%.

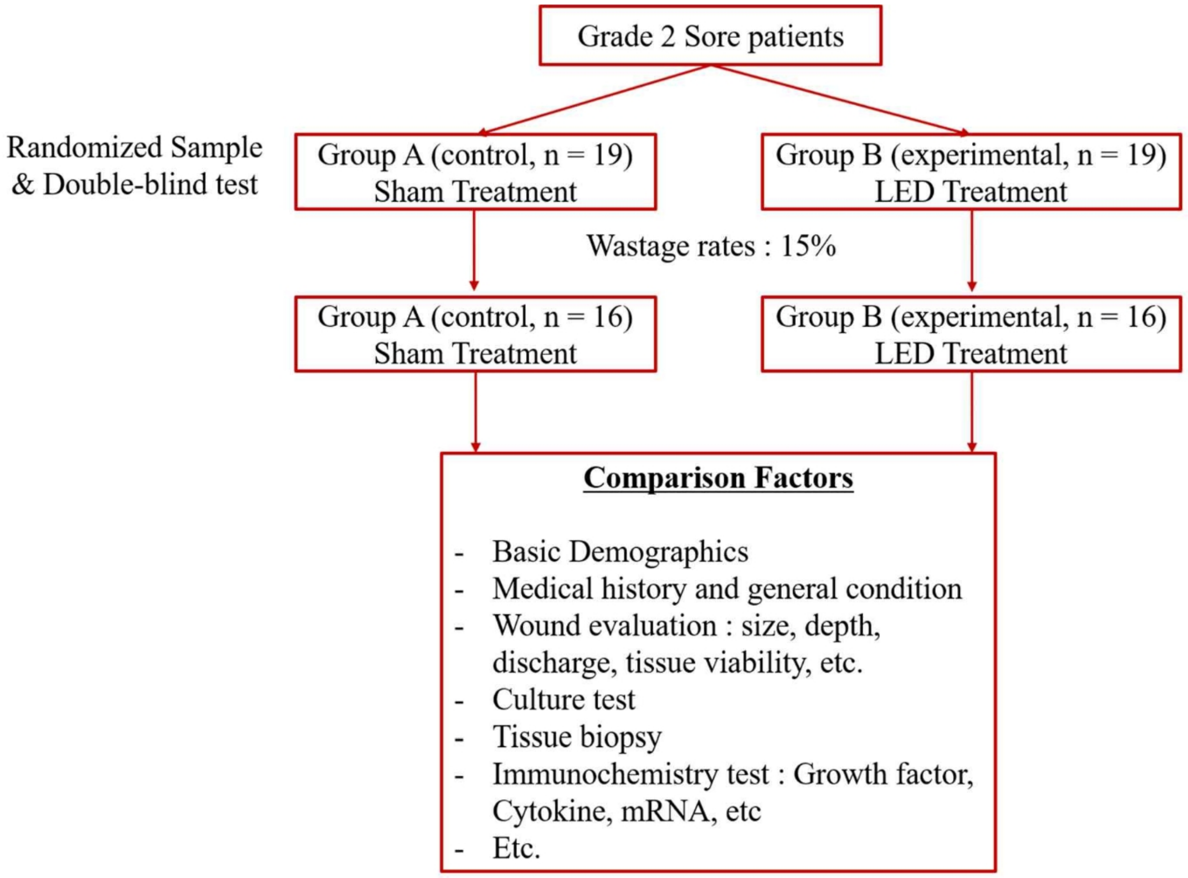

Figure 12. Block diagram of this study

8 Study duration

The duration of the study will be **30 months from the date of Institutional Review Board (IRB) plan approval**. The expected duration of each phase is as follows

1) **Report IRB clearance**: 2 months

2) **Subject recruitment period**: 13 months

Inclusion Criteria: Stage 2 pressure ulcers are currently seen at an average of 10 new patients per month at the center. Of these, an average of 5 patients per month meet the exclusion criteria. Therefore, if we exclude those who do not consent to the study, we **expect to have an average of 3 study participants per month**. We estimate that it will take 13 months to recruit 39 subjects.

3) **Subject study duration**: 12 months

Pressure ulcers are chronic wounds that take a long time to fully heal when treated conservatively. **Mild cases** require **1-3 months** and **moderate cases require at least 6 months to 1 year. In** addition, there **is a wound maturation period up to 6 months after healing, so follow-up is necessary even after the wound has healed**.

However, healing of the wound is not always achieved, and is dependent on the management of unresolved indirect causes, such as internal medicine, nutrition, and ongoing wound care, in addition to relief of the direct causative pressure. **Sufficient clinical time may be required for** this.

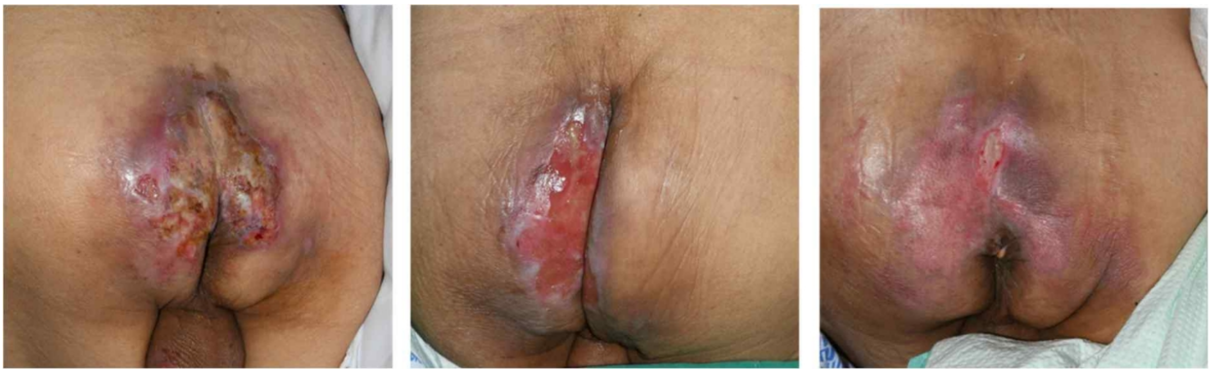

Figure 13. A stage 2 pressure ulcer in the hip area that healed over 3 months with conservative treatment.

4) **Clinical result analysis (statistical processing period) and report preparation**: 3 months

However, the timeframe may change if circumstances arise that may affect the progress of the trial.

	2020		2021				2022			
	3/4	4/4	1/4	2/4	3/4	4/4	1/4	2/4	3/4	4/4
IRB approval										
Recruiting subjects										
Clinical trials										
Analyze results and Build reports										

Figure 14. A schematic of this study

9 Clinical Trial Methods

9.1 Designing a clinical trial

For subjects who signed the informed consent form of their own volition, after conducting the necessary examinations and tests according to the protocol, reviewing the inclusion and exclusion criteria, and deeming them suitable for this study, educating them about the study schedule and methods. The subjects will be **irradiated with 4 wavelengths of LEDs at maximum power (5 levels, ^{90} mW/cm2) for maximum time (25 minutes) at a distance of 15 cm from the pressure ulcer in the buttock area**. **LED irradiation is performed 3 times per week, with a medical examination and wound assessment at the end of each week's treatment. This is done for a total of 4 weeks**.

9.2 Clinical Trial Methods

9.2.1 Prepare subjects

- The principal investigator selects appropriate subjects who meet the subject selection criteria and who voluntarily agree to participate in the trial.
- Explain to subjects the purpose and methods of the study, the safety and effectiveness of the investigational device, and the methods of investigation.
- Completed informed consent forms for clinical research.
- Describe any side effects that may occur after the trial.

- Determine whether adverse events occurred periodically during the follow-up period.

When applying a medical device, the following procedures are implemented

1) Before applying the device, disinfect the area with a sterile cotton pad soaked in saline solution.

2) Medical Device Applications (25 minutes)

*Medical device application details

<Subjects will be irradiated for a maximum time (25 minutes) with 4

wavelengths of LEDs at 5 levels of maximum power [level 1 (12

mW/cm2), level 2 (27.9 mW/cm2), level 3 (44.6 mW/cm2), level 4 (61

mW/cm2), level 5 (90 mW/cm2)] at a distance of 15 cm from the

pressure ulcer on the hip area. LED irradiation is performed 3 times per

week>.

3) After applying the device, disinfect the affected area with a disinfectant swab soaked in saline solution.

4) Apply an antibacterial ointment (Silmazin, Bactroban, Repigel, etc.).

5) Dress the wound using a medform.

After one week of treatment, we will perform an otologic examination and examination of the affected area. This will be done for a total of 4 weeks.

Efficacy will be assessed using wound size and degree of re-epithelialization as primary measures and immunochemical markers on biopsy as secondary measures. Safety will be assessed by physical examination and vital signs, as well as adverse event investigation.

Antibiotic therapy and wound disinfection, which are routinely performed in the conservative treatment of pressure ulcers, will be administered as standard, with the addition of light therapy for those participating in the trial. The light is a combination of four wavelengths and is predicted to be beneficial for bactericidal (BLUE wavelength) and tissue regeneration (AMBER and RED wavelengths).

9.2.2 Setting up a test/control group

Patients were randomized into two groups, one with the investigational device and the other with a sham device. The purpose of the study is to prove the **superiority** of the device in treating pressure ulcers.

This medical device product is a class 2 medical combination stimulator, which is a light emitting diode (LED) device that combines a low power light irradiator (output light with an energy density of 20J/cm2 or less than 2W/cm2) and an infrared irradiator. The irradiation part is approximately 336 × 354 × 105 mm in size, and is designed to be easily applied to the desired location by adjusting the angle and direction at the head connection part, and the height of the product at the body part.

This is a prospective, exploratory, double-blind, randomized, parallel-group, prospective study with a control device (Sham device), which is identical in appearance to the study device and will be applied to control patients. The irradiated area of the control device is made to look similar to the irradiated area of the test device using LEDs of 12 mW/cm2 or less. Patients are randomly assigned to either the test or control device with a 50% chance of being assigned to the test device upon consent.

9.2.3 Randomization

Subjects who meet the inclusion and exclusion criteria and agree to participate in the study will be assigned a subject identification code once they are finally selected. The order in which subjects are assigned to the treatment and control groups according to the subject identification number will be determined by the block randomization method using the statistical program "R" to assign randomization numbers of sufficient size, taking into account the predetermined block size. Once the randomization table is created, it is independently managed by a third party independent randomization manager.

9.2.4 Double Blindness (Blinding)

1) Blinding subjects: subjects, investigators

2) Blind Method

- To maintain blinding, blinding numbers will be managed by a third party independent randomization administrator, and investigational medical devices will be repackaged and labeled with blinding numbers as directed by the independent randomization administrator and sent to the study site.
- Subject blinding: The test and control devices are identical in appearance so that subjects do not know which group they are in, and the medical device manager does not tell subjects which device they have been assigned.
- Investigator blinding method: Investigational medical devices are labeled with a blinding number and sent to the study site under the direction of a third party independent randomization administrator, who verifies the labeling to ensure that subjects use the assigned device and does not inform the investigator.

3) Unblind

- Two sets of emergency code breakers are provided for the blinded study, one set retained by the study site and one set sent to the medical device manager.

- The medical device manager seals each subject's blinding code in a separate envelope and delivers it to the principal investigator.
- The blind decoding table is enclosed in a cover sheet that can be easily peeled off and removed. In the event of an emergency, the cover can be removed to identify the medical device being used on the subject.
- The cover sheet should only be removed in an emergency. If the cover is removed by the Principal Investigator, the Principal Investigator must record the date and time of the removal of the blinding code and the specific reason for the removal and document this in the "Closure" section of the case record.
- In addition, the Principal Investigator must immediately notify the Monitor and the Granting Organization of this unblinding.

9.3 How to use medical devices

9.3.1 Before you start

- Before using the product, determine whether the area around it is subject to intense electricity, magnetic fields, or exposure to heat or humidity, and avoid the area if applicable.
- The power cord must be plugged into a grounded AC 220 V, 60 Hz outlet.
- Set up your device on a stable, level surface.
- Verified that the device is working properly.

9.3.2 Application and duration of use

- **Site of application**: pressure ulcer on subject's hip area
- **Runtime**: 4 wavelengths of LEDs (460 nm : BLUE, 595 nm : AMBER, 630 nm : RED, 850 nm : NIR)

  irradiated for the maximum time (25 minutes) at full power (5 steps, 90 mW/cm^2).
- **Sham device**: 25 minutes, just like the experimental group. For details on how to use the sham device, see

  As described in 9.3.6.

9.3.3 How to operate or use

1) Before using the product, subjects and investigators wear protective eyewear to protect their eyes.

2) Position the light source at least 15 cm from the affected area.

3) Activate the product by pressing the switch on the back of the base.

4) button to select the desired wavelength.

5) Adjust the time and press the Run button to proceed. Press the Run button again to stop the device.

6) The device will automatically stop working after the selected time.

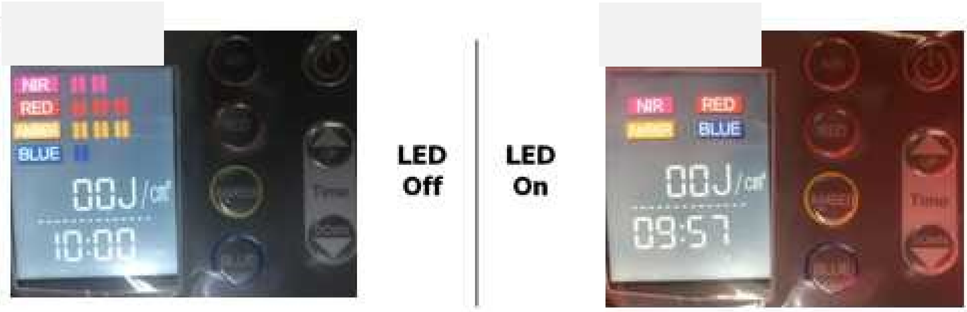

순서	설명
파장 및 세기 선택	파장 선택 및 광량 세기 조절<br>- 최대4개 파장 선택 가능<br>- 5단계 제어- 버튼을 1번 누르면 제일 약한 세기가 선택<br>3번 누르면 중간 세기가 선택<br>5번 누르면 제일 강한 세기가 선택<br>6번 누르면 파장 선택이 없어집니다
Time	UP/DOWN 버튼으로 시간 조절<br>(5분 단위) 최대 25분-예시) 5분→10분→15분→20분→25분
On/ Off	작동 버튼를 이용하여 ON/OFF 동작

Figure 15. 'BELLALUX Lite' control buttons and operation guide

9.3.4 How to store and manage after use

1) Unplug the power cord when you're done and take care of it.

2) Store the device in a designated location.

3) Store away from water or moisture.

4) Stored under stable conditions, such as inclination, vibration, and shock (including during transportation).

5) Moves the device so that the head is in front of the device when it is moved.

6) To clean the appliance, do not spray the spray cleaner directly on the appliance, but spray it on a dry cloth and wipe only the surface of the appliance.

7) Always power off your device before wiping it.

8) Not stored together with chemicals or where gases are generated.

9) Store out of direct sunlight.

9.3.5 Cautions for use

1) Do not operate the device except by authorized personnel and medical personnel.

2) Install in a location that is not adversely affected by air pressure, temperature, humidity, wind, sunlight, air containing salts, ions, etc.

3) To avoid the risk of electrical shock, this device must only be connected to a supply with a protective earth ground.

4) Mutual interference from nearby high-frequency generating electronic medical devices may cause inoperability.

5) Keep a distance of at least 15 centimeters between you and the irradiator.

6) During light irradiation, the subject wears safety glasses for eye protection.

7) Do not move the device during device motion.

8) Immediately stops the operation of the device when an abnormality is detected in the subject and takes appropriate action to ensure the subject is safe.

9) Prevents the subject from touching the device while it is in motion.

10) Not blocking the fan holes in the instrument's light irradiator.

11) It can be affected by conducted and radiated noise, and to minimize interference, other devices

and the
Use from a distance of at least 1 meter.

12) When cleaning the device, wipe only the surface of the device and do not use a spray sanitizer.

9.3.6 About control (SHAM) devices and how to use them

The control device is a device with the same geometry as the test device, with the light irradiation part consisting of LEDs of 12 mW/cm2 or less.

to look similar to the light beam irradiated by the test device, and other appearance and operation

The method is the same as for the tester.

9.3.6.1 Control (sham) device appearance

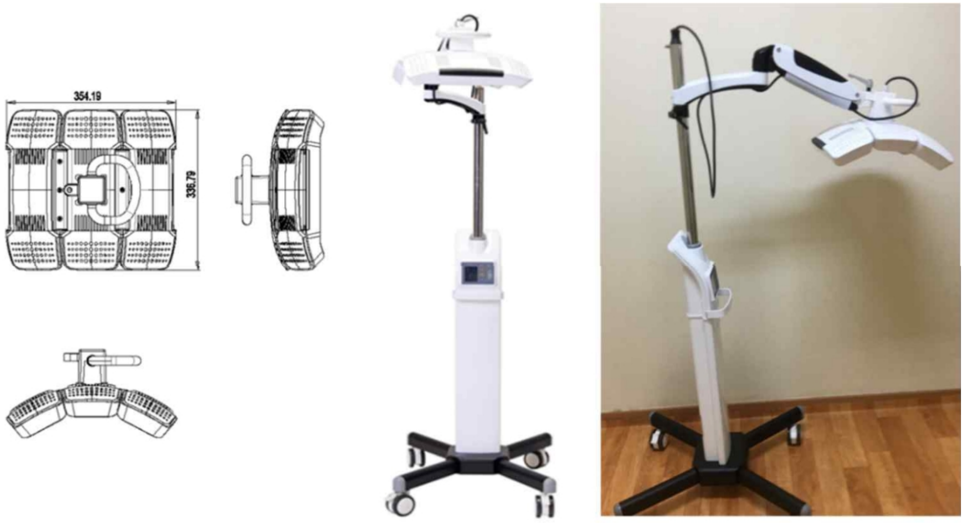

Figure 16. Sham device appearance

9.3.6.2 Controller performance

- Output: 12 mW/cm2
- Time: 25 minutes

9.3.7 Combination Therapy

In principle, the simultaneous use of other medical devices or medicines that may affect the efficacy, safety, and product performance of the investigational medical device during the clinical trial period is prohibited; however, if the use of other medical devices or medicines is unavoidable due to the treatment of subjects and ethical circumstances, the details of the use

should be recorded. In the **case of pressure ulcers, continuous wound disinfection** is **essential for conservative treatment of chronic wounds**. If disinfection is excluded, the wound may deteriorate and cause harm to the patient.

Therefore, **all subjects participating in the study (both control and experimental)** will **continue to be disinfected daily with a 1% Silver Sulfadiazine + Foam formulation based on national guidelines for pressure ulcers published in 2019**.

10 Observation items · Clinical examination items and observation methods

10.1 Clinical trial timeline

Visit	スク Lining	Therapist				Monitoring	
	Visit 1	Visit 2-4 (Week 1)	Visit 5-7 (Week 2)	Visit 8-10 (Week 3)	Visit 11-13 (Week 4)	Visit 14	Visit 15
Viewpoint (± 2 days)	D0	D1,D3,D5	D8,D10,D12	D15,D17,D19	D22,D24,D26	D29	D210
Clinical trial description	○						
Obtaining informed consent and screening numbers Give arcs	○						
Conformance determination (selection /Exclusion Criteria)	○						
Demographics	○						
Medical history (past medical history, surgical history, Drug Dosing)	○	○	○	○	○	○	
Pregnancy test (fertility for women)	○						
Medical exams and Vital signs	○	○	○	○	○	○	○
Annulus and Braden scale side 正	○	○	○	○	○	○	○
Measuring pain scores	○	○	○	○	○	○	○

**Laboratory tests1 (blood tests, urinalysis f)**	○					○	
**Laboratory tests2 (pelvic X-ray)**	○						
**Medical Device Applications**		○	○	○	○	○	
**Laboratory tests3 (fungal culture, tissue biopsy)**		○<br>(Proceed with D1 only)				○	○
**Recheck selection criteria/exclusi on criteria**		○<br>(D1 Advance					
**Suitability determination and subject identification numbers Grant**		○<br>(Proceed with D1 only					
**Investigating adverse events**		○	○	○	○	○	

10.2 Observations and clinical test items

- **Patient consent, screening number assignment, and demographic** survey: Before entering the study, the purpose and content of the study will be explained in detail to the subject, written consent will be obtained, a screening number will be assigned in the order of receipt of written consent, and demographic information will be collected. The following information will be recorded: whether or not written consent was given and the date of consent, subject's initials, gender, date of birth, address, and contact information.

- **Determine eligibility and assign a subject** identification **number**: If all of the subject inclusion criteria are yes and all of the subject exclusion criteria are no, the subject is determined to be eligible and assigned a subject identification code.

- **Medical history**: A detailed investigation and recording of the subject's medical history through a questionnaire and check of past medical records during the screening visit. This includes past history of diabetes, hypertension, surgery, medications, etc.

- **Medical examination**: A medical examination is performed at each visit, and any significant findings are recorded in the Medical Examination section of the case record,

and if significant medical findings that meet the definition of an adverse event are noted after study initiation, they are recorded in the Adverse Event case record. However, undesirable medical events that occurred prior to study initiation should be recorded in addition to the current medical history section.

- **Vital signs**: Checking the subject's vital signs at each visit, including temperature, blood pressure (systolic, diastolic), and pulse rate.
- **Wound assessment**: The investigator will assess the size (width×length×depth), color, presence of necrotic tissue, discharge, odor, microvascular bleeding, and granulation tissue formation of the pressure ulcer at each treatment visit.
- **Erythema: Asymptomatic (0 points), mild (1 point), moderate (2 points), and severe (3 points).**
- **Urticaria: Rate as asymptomatic (0 points), mild (1 point), moderate (2 points), or severe (3 points).**
- **Blisters**: **asymptomatic (0 points), mild (1 point), moderate (2 points), and severe (3 points).**
- **Measure the Braden Scale**, an indicator of pressure ulcer risk, with six items (sensory perception, skin moisture, activity level, positioning, nutrition, friction, and shear) to determine a score (1-4 points for each item, for a maximum of 24 points).
- **Numeric Rating Scale (NRS): A tool for assessing a patient's pain.**

Express it on a scale of 1 to 10. This is measured by asking the patient to rate their pain from 0 for no pain to 10 for unbearable pain.

- **Other inspections**

  - **Pregnancy** test: A pregnancy test is performed on women of childbearing age at the screening visit to confirm that the subject meets the inclusion and exclusion criteria, and the pregnancy is recorded in the case record.

  - **Laboratory test 1**: Laboratory tests are performed to confirm that subjects meet the selection and exclusion criteria, and the test items are as follows. If the test was performed within 2 weeks, it can be replaced.

    ✓ **Blood tests**: CBC, ESR, CRP, biochemistry, etc. are performed at screening, Visit 14, and normal test values and clinical significance are recorded in the case notes.

    CBC - WBC, Hb, Platelet

    Electrolyte - Sodium(Na), Potassium(K)

    Chloride(CL), BUN, Creatine, AST, ALT, Protein

    Albumin, Total Bilirubin, Glucose

    ESR, CRP

    ✓ **Urinalysis**: performed at screening, Visit14, with normal values and clinical significance documented in the case record.

    Protein, Glucose, Ketone, Occult Blood, Bacteria, etc.

  - **Laboratory test 2**: A pelvic X-ray will be taken to confirm the presence of osteitis in order to select subjects and confirm that they meet the exclusion criteria.

  - **Laboratory tests3** : Bacteriologic identification and histologic examination will be performed immediately before study treatment (D1), immediately after study treatment (D29), and at long-term follow-up after study treatment (D210) to observe the change process.

    ✓ **Bacterial identification test**: Bacterial identification test performed on a tissue sample. Test results take about 7 days.

    ✓ **Histologic examination**: The process of tissue changes in the wound is identified through histologic examination and immunochemical examination (IL-1, 4, 6, 10,

13, TNF-a, TGF-b, MMP-1, 2, TIMP, Colla1, 3, etc.
Understand the recovery process.

✓ **This test is performed using a 3- to 4-mm punch biopsy and is only performed on individuals who have given their consent**.

✓ **The test is necessary for the treatment of pressure ulcers, which are invasive or chronic infectious wounds,** and the test does not contribute to **delayed wound healing or harm to the patient**. The following example shows the wound healing process of a patient with a pressure ulcer who had a bacterial culture and biopsy performed, demonstrating that the test was not harmful to the patient's wound healing.

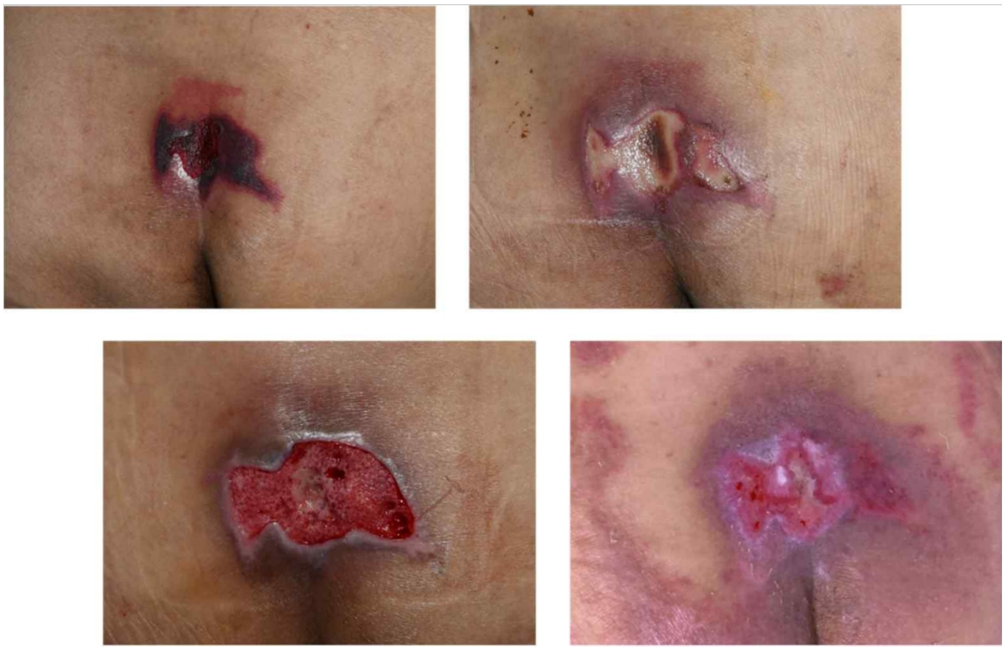

Figure 17. Example of spontaneous wound healing in a pressure ulcer patient with bacterial culture and histology.

- **Confirmation of adverse events**: Information on adverse events is taught to the subjects to report voluntarily from time to time, and the investigator confirms the occurrence of adverse events through questioning. In the event of an adverse event, the date of onset and disappearance, the extent and consequences of the adverse event, the measures taken in relation to the irradiation and the causal relationship with the irradiation, the name of the suspected drug or treatment other than irradiation, and whether and how the adverse event was treated are recorded in detail in the case record sheet.

- **Expected adverse events in this study (**based on a 2014 Cochrane review by Chen C. et al)

  ■ Possible side effects include increased exudate, bacterial growth, wound bleeding, and tissue necrosis due to stimulation of the inflammatory response.

- A 2014 Cochrane review by Chen C. et al. found that photobiologic therapy can also cause side effects such as pain, microbleeds, and redness.

10.3 Observational Test Method

10.3.1 Screening Visit (Visit 1, -D14 to D1): Subjects selected to participate in this study will receive an explanation of the study and be assessed in the following order.

Explain the test process and obtain written consent from subjects before allowing them to participate in the test.

Subjects are corrected on their screening numbers in order.

③ Investigate the subject's demographics and medical history.

④ For women of childbearing age, perform a pregnancy test.

Perform a physical exam and vital signs.

⑥ Measurement of erythema, Braden scale, and NRS.

⑦ Blood tests, urinalysis, and pelvic X-ray.

10.3.2 Visit 2 (D1 ± 2 days)

Recheck the selection criteria and exclusion criteria before irradiation, then judge the suitability and assign the subject number.

Medical examination and measurement of vital signs

Measures area assessment, Braden scale, and NRS

④ Phototherapy performed.

⑤ Performed bacterial identification and histologic examination

Checked medical history and medications.

⑦ Check for adverse events.

10.3.3 Visit 3 (D3 ± 2 days) to Visit 13 (D26 ± 2 days)

Medical examination and measurement of vital signs.

Measurement of erythema, Braden scale, and NRS

③ Performed phototherapy.

④ Checked medical history, medications.

⑤ Check for adverse events.

10.3.4 Visit 14 (D29 ± 2 days)

Medical examination and measurement of vital signs.

Measures area assessment, Braden Scale, and NRS

Performed blood tests, urinalysis, mycology tests, and biopsies.

④ Checked medical history, medications.

⑤ Check for adverse events.

10.3.5 Visit 15 (D210 ± 2 days)

Medical examination and measurement of vital signs.

Measurement of erythema, Braden scale, and NRS.

Performed mycobacterial culture and histologic examination.

11 Possible side effects and precautions for use

11.1 Possible side effects

- Possible side effects include increased exudate, bacterial growth, wound bleeding, and tissue necrosis due to stimulation of the inflammatory response.
- A 2014 Cochrane review by Chen C. et al. found that photobiologic therapy can also cause side effects such as pain, microbleeds, and redness.
- If you have an adverse event, record the name of the event and the severity of the symptoms in the case log.

11.2 Cautions for use

- Do not shine the light directly into your eyes. Shining the light into your own eyes, shining it into the eyes of others, looking at it through a magnifying glass, or looking at its reflection in a mirror can cause temporary impairment of your eyes, and prolonged exposure can be harmful to your eyes.
- People with light-sensitive bodies or those taking related medications may be harmed by this product, so please consult a professional before using it.
- When using this product, you must follow the instructions in the user manual.

12 Stop and drop criteria

12.1 Stop by

- If an adverse event, adverse reaction, etc. is observed during the course of the trial and it is deemed unreasonable to continue the trial, the principal investigator must request the IRB to stop the trial, and the trial may be stopped based on the IRB's decision.
- If the sponsor wishes to stop the clinical trial due to reasons such as the safety of the investigational medical device, the sponsor may request the Institutional Review Board to stop the clinical trial and stop the clinical trial in accordance with the decision of the Institutional Review Board.

12.2 Elimination Criteria

- Record whether all subjects in the study have completed the study, and if irradiation or observation has been stopped, record the reason. For subjects in an ongoing clinical trial, the study may be discontinued in the following cases
  - ■ Violation of inclusion and exclusion criteria
  - ■ Use of prohibited medications during the treatment period: steroids, immunosuppressants
  - ■ If a subject has a serious adverse event, or if the subject requests to discontinue the study due to an adverse event, or if the investigator believes that the adverse event warrants discontinuation of the study.
  - ■ Subjects with systemic disease that was not detected by pre-study testing.

- The subject or the subject's legal representative due to unsatisfactory treatment effectiveness during the clinical trial.

If a proxy asks to stop the test

- Violation of the protocol by the investigator or subject.
- Subject withdraws consent to participate in a clinical trial
- If a subject can't be tracked
- If you're having trouble irradiating your subjects
- The development of any other condition requiring immediate medical attention that, in the opinion of the investigator, makes it inappropriate for you to proceed with the study.

12.3 Handling stops and dropouts

- If a subject drops out or is withdrawn during the study, this is documented in the case record and followed up with questionnaires or phone calls for possible adverse events.
- Dropouts will be included in the statistical processing of safety and efficacy assessments unless there is good reason or evidence to exclude them, and analyses will be conducted using the Last Observation Carrying Forward (LOCF) method, which replaces missing values with the value immediately prior to the dropout.

13 Evaluation criteria, methods, and interpretation of validity (based on statistical methods)

13.1 Primary efficacy measures: wound size and degree of re-epithelialization

- Wound size and degree of re-epithelialization in the control and experimental groups at 0 and 4 weeks were plotted against time, respectively, and differences between the two groups were analyzed using a paired t-test.
- Wound size and degree of re-epithelialization will be measured by the investigator on the day of the study visit by physically examining the pressure ulcer size (width × length × depth: measured in cm using a ruler). Photographs of the treated area will be taken, including a 1 cm X 1 cm grid at the time of measurement.

*How to take clinical photographs: At the time of each visit, photographs should be taken at a 90° angle and at a distance of 15 cm, using the camera in use in the clinic. For consistent evaluation, ensure that the photographs are taken under the

same conditions (angle, same camera, etc.).

13.2 Secondary validity measure: immunochemical markers on tissue examination

- Pro-inflammatory cytokine (IL-6) / Anti-inflammatory cytokine (IL-10) ratio was obtained and the progression to the proliferative phase was analyzed by comparing the control and experimental groups after 0 and 4 weeks.
- Immunochemical markers are measured in a laboratory by a researcher through RNA sequencing, with final confirmation by the Principal Investigator.

14 Criteria for evaluating safety, including adverse events · Methods of evaluation and reporting

14.1 Definition of an adverse event, adverse reaction

14.1.1 Anomalies

- **"Adverse Event (AE)"** means any unintended condition, symptom, or disease that occurs in a subject during a clinical trial, including abnormalities in laboratory test results, that is not necessarily causally related to the investigational medical device.

14.1.2 Medical Device Adverse Events

- **"Adverse Device Effect (ADE)"** means any harmful, unintended reaction caused by an investigational medical device that cannot be causally linked to the investigational medical device.

14.1.3 Serious adverse events, medical device adverse events

- **"Serious AE, ADE" means an** adverse event or medical device event caused by a medical device used in a clinical trial that meets any of the following criteria

Death or danger to life

You need to be hospitalized or have an extended hospital stay.

Resulted in permanent or significant disability and impairment.

The fetus has malformations or abnormalities.

14.1.4 Unexpected Medical Device Adverse Events

- "**Unexpected Adverse Device Effect"** means a difference in the appearance or severity of a medical device **adverse event in** light of available information about the medical device, such as a clinical investigator's data sheet or medical device supplement.

14.2 Evaluating adverse events

- If an adverse event occurs, it should be reported using the following severity scale

14.3 Causality with clinical devices

- The relevance of the adverse event to the investigational device is assessed by the investigator by the following criteria and described in the investigator's opinion.

A. Definitely related.

(1) There is a discrepancy in the temporal sequence of investigational use and adverse event occurrence.

The adverse event is most likely explained by the use of the investigational clinical device rather than any other reason.

③ The adverse event disappears with discontinuation.

④ Reuse (only performed if reuse is available) If the result is positive.

The adverse event is consistent with information already known about the clinical device or its class of medical devices.

B. Probably related

There is evidence that the clinical device was used.

The temporal sequence of the use of the clinical device and the onset of the adverse event is reasonable.

The adverse event is more likely to be explained by the use of the clinical device than by other causes.

U

c. Probably related

There is evidence that the clinical device was used.

The temporal sequence of the use of the clinical device and the onset of the adverse event is reasonable.

The adverse event is believed to be attributable to the use of the investigational clinical device to the same extent as other possible causes.

④ The adverse event resolves with discontinuation of use of the clinical device (if implemented).

d. Probably not related

There is evidence that the clinical device was used.

The clinical device discontinuation result is negative or ambiguous.

Clinical device reuse results are negative or ambiguous.

d. Definitely not related

No clinical devices were used on the subject.

The temporal sequence between the use of the clinical device and the discovery of the adverse event is not plausible, and there is an apparent circle of causation for the adverse event.

BAR. Unevaluable (Unknown)

Information is insufficient or conflicting to make a judgment, and cannot be supplemented or verified.

14.4 Evaluation Criteria

- Subject's complaints of adverse events that occurred during or after use of the medical device: whether there were symptoms of irritation in the hip area (burning, hives, inflammation, itching, pain, etc.)
- Medical examination: presence of skin lesions (erythema, edema, etc.) in the hip area
- Follow-up period: Follow-up will be conducted until 6 months after the end of the study.
- Mild

  Evaluate the extent of the medical findings and their causal relationship to the clinical device if they do not interfere with the subject's normal daily life, cause minimal discomfort and are easily tolerated by the subject, the treatment is testimonial and complete recovery does not affect the validity of the medical device.
- Severe (moderate)

  If it causes discomfort that significantly interferes with the subject's normal daily life, is reversible with continued treatment, and does not affect the validity of the investigational medical device, the degree of medical diagnosis and the causal relationship to the

investigational medical device will be evaluated.

- severe

Evaluate the validity of the medical opinion and its causal relationship to the investigational clinical device if it prevents the subject from performing normal activities of daily living, is incurable, and if the investigational clinical device affects validity.

14.5 Evaluation Methods

-Perform analyses on adverse events reported by subjects or identified by secondary review to assess the severity and type of adverse events and the incidence of adverse events in the study and control groups.

14.6 Reporting adverse events

1) Severity of adverse events: Allows you to indicate mild, moderate, severe, etc. in the adverse event report card.
2) Causality with an investigational medical device: Determine causality based on the evaluation criteria and display it in the adverse event record table.
3) Treatment: Allows you to display in the record table if there is a change in the clinical trial method, such as a change in the method of use, a decrease in the number of uses, or a discontinuation of use due to an adverse event.
4) Treatment course: Allows you to describe the disappearance or worsening of an adverse event due to treatment after the event occurred.
5) Investigator's view: Allows you to record the investigator's view of an adverse event.

14.7 Reporting method: This information is reported in accordance with the regulations on medical device clinical trials and the standard work instructions prescribed by the medical device clinical trial center.

1) An adverse event is any unintended symptom, manifestation, or disease that occurs in a subject during a clinical trial, and should be recorded in the case report based on the medical findings and severity of the adverse event and the evaluation of the causal relationship with the investigational medical device. Therefore, the criteria for evaluating the causal relationship between an adverse event and an investigational medical device should be presented.

2) Safety criteria refers to the criteria for assessing the severity of predicted adverse events and adverse events as they should be assessed and recorded in the case record.

3) Safety evaluation methods are statistical analysis methods and evaluators for the comparative evaluation of adverse events, adverse medical events, and the frequency of adverse events associated with investigational medical devices between test and control groups.

Presented quasi-.

4) Adverse Event Reporting: The Principal Investigator must promptly notify the Sponsor of all serious adverse events and make a detailed, documented follow-up report in accordance with the protocol. In the case of a fatal or life-threatening event, a further report must be made to the sponsor within 7 days of the date the sponsor receives or learns of the event, in which case detailed information about the adverse event must be provided within 8 days of the initial report. In the case of other serious and unexpected adverse medical events, the sponsor must report each to the head of the Ministry of Food and Drug Safety within 15 days of the date the sponsor receives or learns of the event.

15 Informed consent form (attached)

16 Victim compensation protocols

In the event of any damage or harm related to this clinical trial, **the clinical trial sponsor (Link Optics Co., Ltd.)** will take **full legal responsibility and compensate** for the **damage,** and side effects and worsening of the disease will be treated with known treatment methods. However, **other injury-related or disease-related expenses such as lost wages will not be compensated. The** study physician and research staff will provide you with additional information about all other possible financial compensation.

temporary pain or damage that can be easily treated, and the site determines that treatment is necessary (coverage is limited to the cost of the necessary treatment).

You need to be hospitalized or have your hospital stay extended.

Causes persistent or significant disfigurement or diminished donation.

④ Causes birth defects or abnormalities

Causes death or threatens life.

16.1 Reward requirements

Subject compensation under this Compensation Agreement is subject to the following requirements

Physical injury caused by the investigational clinical device.

The subject's condition has worsened as a result of the clinical trial.

③ The investigator has complied with all aspects of the protocol approved by the Korea Food and Drug Administration.

④ was not caused by the tester's manifest negligence or dereliction of duty; and

The subject has complied with all instructions given by the principal investigator or study staff.

⑥ The subject has taken steps to minimize the incidence of damages resulting from the bodily injury.

16.2. Reasons for Exclusion

Damage due to insufficient effectiveness, efficacy expected from an investigational clinical medical device

Skin wrinkles and skin elasticity do not improve with the use of this clinical device.

Damage caused by the subject's negligence

If it hasn't been used on a given body part for a given amount of time.

: Failure to seek counseling and medical attention in the event of an adverse event.

Failure to comply with subject precautions.

< Note to subjects

1) People with photosensitivity should not use it.

2) Use caution when combining medications and foods that increase photosensitivity as they may cause damage, such as burns, to the area where the device is used.

- Drugs known to cause photosensitization : Quinolones (nalidixic acid, ofloxacin, sparfloxacin, etc.), tetracyclines (minocycline hydrochloride, doxycycline hydrochloride, etc.), sulfa drugs, griseofulvin, tar preparations, ciclosporins (etsiagit, cyclobenzazid, fenfluthizide, etc.), porphyrins (NAPP, etc.), phenothiazines (prochlorperazine, chlorpromazine, etc.), methylene blue, etc.

- Foods that increase photosensitivity

: Foods containing furocoumarin (celery, lime, carrots, parsley, figs, mustard, etc.)

3) Not for use by pregnant or lactating women.

4) Do not expose the product to excessive humidity, overheating, or overcooling.

5) Do not use the product when it is wet or leaking water.

6) The product will overheat and should not be used continuously for more than 30 minutes.

7) If the light irradiator is heavily contaminated, the product efficiency will be reduced, so decontaminate it with a dry towel before use.

8) When the product is in operation, the patient should not look at the illuminated light source with their eyes.

9) It should not be used on open wounds.

10) To prevent electrical shock, store away from contact with magnets or energized objects.
11) You may not modify, disassemble, or repair the product in any way.
12) If you feel uncomfortable while using the product, stop using it.

16.3 Reward criteria

If there is a pre-agreed compensation amount or measure between the parties for an anticipated medical device adverse event, we will compensate according to that standard.

In other cases, compensation shall be provided in accordance with the compensation method agreed upon between the parties, taking into account the extent, nature, duration, and similar cases of physical damage.

③ If the parties do not agree on the preceding paragraph, compensation shall be provided in accordance with the court's judgment and the confirmation of the corresponding decision.

16.4 Compensation Process

Subjects who suffer bodily injury under this Compensation Policy should contact the principal investigator of the clinical trial or the clinical trial center for necessary medical treatment.

Subjects whose physical injuries are not cured despite the actions of the principal investigator or the institution may request compensation from the sponsoring organization.

③ After receiving the above compensation request, the sponsoring organization shall promptly investigate whether the compensation is applicable and the compensation standard, and notify the subject about it.

④ The subject must notify the sponsoring organization within five (5) business days from the date of receipt of the above notification of any objections to the above notification.

⑤ If the Subject fails to notify us of any objection after receiving the notice in paragraph (3), the Parties understand that we have agreed to compensation in accordance with the above notice.

⑥ If the subject notifies an objection in accordance with the provisions of Paragraph (4), the sponsoring organization shall recommend multiple objective experts to the subject to determine whether the subject is eligible for the above compensation and the compensation standard, and the subject shall nominate one such expert within three (3) business days from the date of the above recommendation. (If the subject does not nominate, the sponsor shall select one at

random.)

16.5 Coverage

(1) This Compensation Agreement applies to subjects participating in all clinical trials sponsored by the Sponsoring Organization.

unless there is a different arrangement between them.

Any agreement that a subject enters into with any other third party in connection with a clinical trial that is not approved by the Sponsor for compensation for the clinical trial is not enforceable against the Sponsor.

We take care to ensure that subjects do not suffer any disadvantages as a result of this study, and we pledge to be responsible under the Victim Compensation Policy if any problems arise as a result of this study.

17 Post-trial care for subjects

Patients who drop out of the clinical trial or do not respond shall be instructed to receive other appropriate treatment, and patients whose clinical trial is terminated shall follow the treatment procedures of the hospital for subsequent treatment, and the subsequent treatment costs shall be supported by the subject. However, in the event of an adverse event, after confirming the existence or absence of a causal relationship with the clinical medical device used in the clinical trial, if the adverse event is caused by the clinical medical device used in this clinical trial, the sponsor shall pay for the treatment until the adverse event disappears.

18 Research subject risks and benefits

For mild pressure ulcers, conservative treatment is performed and includes antibiotic therapy and wound disinfection, as well as repositioning to relieve pressure. There are many wound disinfection agents available, but each has its own limitations, and there is no clinically established method of disinfection. If you decide not to participate in this study, you will receive usual conservative care, and you will be able to decline phototherapy even during the study, and you will be able to receive usual conservative care immediately afterwards. Therefore, we will strive for recovery of your condition regardless of whether you participate in the study, and we will try to minimize harm to you.

The expected side effects of the study medical device are increased exudate in the hip area, bacterial growth, wound hemorrhage, tissue necrosis, and pain, microbleeds, and redness with photobiologic therapy.

Side effects may occur. Examples of treating adverse events as soon as they occur

In the event of any injury or damage related to this study, the sponsor (LinkOptics Co., Ltd.) will be solely responsible and will compensate for the damage. However, other injury-related or disease-related expenses, such as lost wages, will not be covered.

The expected benefit of participating in this study is that the study will pay for blood tests, urine tests, radiology tests, biopsies, and bacterial identification tests related to the study while you are participating in the study. You will be responsible for any hospitalization and laboratory fees not related to the conduct of this study.

19 Measures to protect the safety of human subjects

19.1 Clinical trial site

The head of the organization conducting the clinical trial shall ensure that the clinical trial is properly conducted by equipping the clinical laboratory, facilities, and specialized personnel necessary for the conduct of the clinical trial and taking necessary measures in case of emergency.

19.2 Institutional Review Board (IRB)

IRBs should be organized in accordance with national laws, regulations, and practices and should protect the rights, safety, and welfare of human subjects. It should also scrutinize the validity of the reasons for enrolling subjects from vulnerable populations in clinical trials. In fulfilling its duties, the IRB must take necessary measures, such as ordering the principal investigator to stop part or all of the trial, if it finds that the subjects' consent to participate in the trial was not properly obtained, if the trial is not conducted according to the protocol, or if serious adverse events or adverse reactions occur.

19.3 Investigator

1) Investigator means the principal investigator, study staff, and study coordinator. The investigator shall conduct the clinical trial in accordance with the protocol agreed with the sponsor and approved by the IRB and the Minister of Food and Drug Safety.
2) During and after the study, the investigator must ensure that the subject receives appropriate medical care for any adverse events that occur in the study, including clinically significant abnormalities in laboratory tests, and must inform the subject of any co-

morbidities that the investigator becomes aware of that require medical attention.

3) The investigator accurately analyzes and understands the study plan and actively responds to subject concerns.

19.4 Sponsor

1) A person with responsibility for the planning, management, and financing of a clinical study, usually the manufacturer (including the importer) of a medical device in the case of a medical device clinical trial.

2) Ensure that the subjects, methods, formats and contents of case reports are followed in accordance with the procedures in the protocol.

3) The sponsor's inspection plan and procedures should be based on the importance of the study, the number of subjects, the type and complexity of the study, the degree of potential risk to subjects, and any known problems in conducting the study.

19.5 Monitoring

1) Monitoring refers to the activity of overseeing the progress of a clinical trial and reviewing and verifying that the trial is conducted and recorded in accordance with the protocol, standard work instructions, good clinical practice, and applicable regulations.

2) Monitoring of clinical trials is accomplished through periodic visits and phone calls by a clinical trial monitor to study sites. During these visits, the monitor will check original patient records, investigational medical device management records, and data storage (study files).

3) Trial monitors keep an eye on the progress of the study and discuss any problems with the investigator.

19.6 Changes to protocols

1) After the protocol has been approved by the IRB and the Commissioner of Food and Drug Safety, any changes to the protocol due to extensive or increased risk, changes in subject selection criteria, or additional safety information must be approved by the IRB and the Commissioner of Food and Drug Safety.

2) When revising the protocol, the date of revision, the reason for revision, and the contents of the revision shall be recorded and kept.

3) Investigators must comply with the IRB and the

The study must not be conducted differently from the protocol until the Commissioner of Food and Drug Safety approves the change. If a protocol change is made prior to IRB approval to eliminate an immediate risk to subjects, the change must be submitted to the IRB, the sponsor, and the Commissioner of Food and Drug Safety as soon as possible. And send a document approved by the IRB chair or secretary to the sponsor.

4) Minor modifications or clarifications that do not affect the study do not require approval and are administrative changes.

19.7 Informed Consent

1) Informed consent refers to the process by which a subject is provided with all information relevant to the study in an informed consent document before deciding whether or not to participate in the study, and confirms that he or she is voluntarily participating in the study by signing and dating the document.
2) If the subject or their representative is unable to read the consent form, subject manual, and other documented information, an impartial party must be present throughout the consent process.
3) Before obtaining consent, the investigator must give the subject or their representative sufficient time and opportunity to ask questions about the details of the study and to decide whether to participate in the thawing study, and must answer all study-related questions to the subject's or their representative's satisfaction.

19.8 Confidentiality of subject records

1) That records that could identify subjects will be kept confidential, and that subjects' identities will remain confidential if the results of the trial are published.
2) The sponsor, monitors, and inspectors involved in this study may have access to the subject's records for the purpose of monitoring and inspecting the study and managing events. By signing this protocol, the investigator acknowledges that the sponsor or monitors and inspectors may review or copy the subject's charts and case notes to verify the subject's records in accordance with local laws, regulations, and ethical standards. Such information must be kept confidential.
3) All documents related to the trial, such as case notes, are recorded and identified with a

subject identification code (usually the subject's initials) rather than the subject's name.

19.9 Retention of records

Ensure that all materials and records related to the conduct of clinical trials are well preserved and secured. After completion of the clinical trial results report, clinical trial-related documents shall be preserved for 10 years from the end of the clinical trial.

19.10 Processing specimens

- All specimens are coded and de-identified.
- Mycobacterial identification specimens: Performing tests and processing specimens in the Department of Diagnostic Laboratories
- Histology specimens: In the event of residual specimens after immunochemical testing at the Translational Clinical Medicine Center in Room 328 of the School of Medicine, they will be disposed of at the medical waste location in the laboratory.

20 What else you need to run a clinical trial safely and scientifically

20.1 Case notes

1) This study uses paper case notes to collect data. When we say subject's documentation, we mean the subject's records from their doctor that are kept at the site. Most often, the source document is a hospital or physician's chart, and all information recorded in the subject's case record must be consistent with the source document.
2) It is the responsibility of the Principal Investigator to record, review, and sign the case notes.
3) After completing the case notes, the Principal Investigator signs each case note to certify that the information in the case note is true. This means that the Principal Investigator has final responsibility for the data related to the study in the case notes.

20.2 Monitoring

1) The Sponsor or an organization entrusted by the Sponsor with monitoring duties will conduct monitoring to protect the rights and welfare of subjects and to verify the quality and reliability of the study data. The sponsor must select the monitor(s) and have documentation of the names and qualifications of the monitor(s). In addition, the monitor

shall ensure that the investigational medical device, protocol, consent form, subject manual and other written information provided to subjects are in accordance with the applicable laws and regulations, the Sponsor's Standard Operating Procedures, and the applicable regulations.

Have sufficient scientific or clinical knowledge to monitor anima, etc.

2) The monitor verifies that the clinical trial-related data is accurate, complete, and verifiable when compared with the supporting documents, and that the clinical trial is conducted in accordance with the approved protocol and relevant regulations, including Article 24 of the Medical Device Enforcement Rules (Standards for Conducting Clinical Trials, etc.).

3) The Principal Investigator and study personnel shall make available to the monitoring personnel case documentation as defined in Good Clinical Practice for Medical Devices (Source documents: hospital or personal charts, records of laboratory results, appointment notes, etc.

20.3 Recording and archiving materials

1) Ensure that there is a designated and secure storage area for all materials and records related to the conduct of clinical trials.

2) After the completion of the outcome report, an archivist is assigned to retain trial-related documents for three years from the end of the trial.

20.4 Submit and publish reports

1) The principal investigator, in consultation with the sponsor or site, will write a report on the content of this study.

2) Publications resulting from clinical trials should specify the participation of the principal investigator and study staff, and agreements regarding the sequence of authorship should be made before the manuscript is written.

3) All summaries, manuscripts, or presentations related to the trial must be provided to and reviewed by the sponsor or site prior to publication or presentation.

4) Delete all materials deemed confidential by the site. In this case, the results obtained in this clinical trial are not considered confidential.

20.5 Contracts

- The sponsor shall enter into a clinical trial agreement with the director of the clinical trial center, which shall include matters related to the financing of the clinical trial, delegation

and division of duties, and obligations of the sponsor and the director of the clinical trial center.
